# Multiple strategies for heat adaptation to prevent chalkiness in the rice endosperm

**DOI:** 10.1093/jxb/ery427

**Published:** 2018-12-03

**Authors:** Hiroshi Wada, Yuto Hatakeyama, Yayoi Onda, Hiroshi Nonami, Taiken Nakashima, Rosa Erra-Balsells, Satoshi Morita, Kenzo Hiraoka, Fukuyo Tanaka, Hiroshi Nakano

**Affiliations:** 1 Kyushu Okinawa Agricultural Research Center, National Agriculture and Food Research Organization, Chikugo, Fukuoka, Japan; 2 Graduate School of Agriculture, Ehime University, Matsuyama, Ehime, Japan; 3 Faculty of Agriculture, Hokkaido University, Sapporo, Hokkaido, Japan; 4 Department of Organic Chemistry, University of Buenos Aires, Buenos Aires, Argentina; 5 Clean Energy Research Center, The University of Yamanashi, Kofu, Yamanashi, Japan; 6 Central Region Agricultural Research Center, National Agriculture and Food Research Organization, Tsukuba, Ibaraki, Japan

**Keywords:** Chalkiness, high temperature, *Oryza sativa*, protein body, protein storage vacuole, redox, starch, vacuole

## Abstract

Heat-induced chalkiness of rice grains is a major concern for rice production, particularly with respect to climate change. Although the formation of chalkiness in the endosperm is suppressed by nitrogen, little is known about the cell-specific dynamics of this process. Here, using picolitre pressure-probe electrospray-ionization mass spectrometry together with transmission electron microscopy and turgor measurements, we examine heat-induced chalkiness in single endosperm cells of intact rice seeds produced under controlled environmental conditions. Exposure to heat stress decreased turgor pressure and increased the cytosolic accumulation of sugars, glutathione, and amino acids, particularly cysteine. Heat stress also led to a significant enlargement of the protein storage vacuoles but with little accumulation of storage proteins. Crucially, this heat-induced partial arrest of amyloplast development led to formation of chalkiness. Whilst increased nitrogen availability also resulted in increased accumulation of amino acids, there was no decrease in turgor pressure. The heat-induced accumulation of cysteine and glutathione was much less marked in the presence of nitrogen, and storage proteins were produced without chalkiness. These data provide important information on the cell dynamics of heat acclimation that underpin the formation of chalkiness in the rice endosperm. We conclude that rice seeds employ multiple strategies to mitigate the adverse effects of heat stress in a manner that is dependent on nitrogen availability, and that the regulation of protein synthesis may play a crucial role in optimizing organelle compartmentation during heat adaption.

## Teaser

This study revealed the cellular dynamics for heat-induced chalky formation and nitrogen-enhanced heat adaptive mechanism by conducting a newly developed on-site cell-specific analysis coupled with time-course analysis via microscopy.

## Introduction

Grain chalkiness is a critical trait that determines the quality of rice (Hoshikawa, 1989). An increase in the occurrence of chalky rice induced by several environmental stresses, such as high temperature during the grain-filling stage (Tashiro and Wardlaw, 1991), is commonly being observed under the influence of global warming (Jagadish *et al.*, 2015; Morita *et al.*, 2016). In part of the rice endosperm, air spaces are formed among the loosely packed starch granules and cause significant random reflection of light, thereby giving them a chalky appearance (Tashiro and Wardlaw, 1991). One of the major forms of chalky rice, called ‘white-back kernel’, which is frequently induced by exposure to high temperatures at the early ripening stage, exhibits chalkiness in the outer endosperm regions longitudinally aligned along the dorsal side of the kernels, where a greater abundance of protein bodies (PBs) are distributed than in the ventral side (Ellis *et al.*, 1987; Hoshikawa, 1989). White-back kernel is known to be decreased by supplying nitrogen (N) prior to the onset of heat stress (Wakamatsu *et al.*, 2008). Since the formation of chalkiness only occurs in a part of the endosperm, this phenomenon is presumably a cell-specific event. However, our current understanding of heat-induced rice chalkiness relies on analyses at the tissue level, and hence the underlying mechanisms in cellular metabolism that lead to the formation of air spaces remains unclear. In addition, the roles of N during the heat response have not been spatially addressed at the metabolite level.

Storage proteins are major components that accumulate to form 5–8% of the rice endosperm (Hoshikawa, 1989). These proteins are typically stored into two types of PBs, called PBI and PBII (Herman and Larkins, 1999). PBIs are small spherical granules of 1–2 μm in diameter with concentric rings of various electron densities that originate from the rough endoplasmic reticulum (rER), and they accumulate prolamins synthesized on the ER membrane (Bechtel and Juliano, 1980; Saito *et al.*, 2012). In contrast, PBIIs are granules with irregular shape that originate from protein storage vacuoles (PSVs). The diameter of mature PBIIs typically range between 2–4 μm, i.e. larger than PBIs, and they store glutelin and globulin synthesized on the rER (Tanaka *et al.*, 1980; Herman and Larkins, 1999).

Numerous studies have examined mutants that exhibit abnormal PB formation in the endosperm (Takemoto *et al.*, 2002; Onda *et al.*, 2009, 2011; Wang *et al.*, 2009; Nagamine *et al.*, 2011; Ren *et al.*, 2014) and interestingly these mutations exhibit chalky phenotypes that are sometimes described as ‘floury endosperm’ (Wang *et al.*, 2010; Fukuda *et al.*, 2011; Li *et al.*, 2014), implying that there may be a close relationship between starch and storage protein biosynthesis. Although changes in starch metabolism have mostly been studied in the context of the formation of chalkiness (Zakaria *et al.*, 2002; Yamakawa *et al.*, 2007; Zhang *et al.*, 2011; Wada *et al.*, 2014; Xi *et al.*, 2014), the activities of several enzymes involved in starch metabolism are known to be affected by reducing or oxidizing conditions (Kötting *et al.*, 2010). Regulation of protein synthesis is likely to be a key factor in modulating the activity those enzymes, as well as in the development of disulfide-rich PBs in the cells. Given the consistency of the spatial localization of PBs (Ellis *et al.*, 1987; Hoshikawa, 1989) and the chalky zone in the dorsal outer-endosperm of white-back rice induced under heat conditions, we hypothesized that cytosolic protein synthesis might be disturbed under heat conditions prior to formation of chalkiness. To test this hypothesis, some appropriate cell-specific analytical method was required.

Recently, the scope of single-cell metabolomics has been expanded due to technical improvements in mass analysers (i.e., Orbitrap mass spectrometer) and applied in plant research (Gholipour *et al.*, 2013; Fujii *et al.*, 2015). A cell pressure-probe, originally invented by Steudle’s research group (Hüsken *et al.*, 1978) and long-used to measure the cellular water status in plants, has been used as a picolitre pipette (quartz capillary) to establish a new type of *in situ* analytical method by combining it with an Orbitrap mass spectrometer by Nonami’s research group (Gholipour *et al.*, 2013). More recently, both the resolution and sensitivity of this analytical method have been improved by introducing an internal electrode in the capillary holder and using a mixture of an ionic solution and silicone oil in the quartz capillary (Nakashima *et al.*, 2016). This method, termed ‘picolitre pressure-probe electrospray-ionization mass spectrometry’ (picoPPESI-MS), referred to as ‘internal electrode capillary PPESI-MS’ (IEC-PPESI-MS) in Nakashima *et al.* (2016), can be performed in intact plants. This type of cell metabolomics appears to provide a robust and powerful method for performing cell-specific analysis. However, most of the cell metabolomics techniques, including this method, have been confined to laboratory use at room temperature. To the best of our knowledge, no attempt has been made to adapt such cell-specific analytical methods to provide a technique that allows investigation of metabolic responses to environmental stimuli, such as temperature responses in developing crop plants.

To address this issue, we have established a new method of cell metabolomics by combining picoPPESI-MS with environmentally controlled conditions that can be used to directly conduct an ‘on-site cell-specific analysis’ in the endosperm cells growing under heat conditions. Under temperature equilibrium conditions, this analytical method allows on-site real-time metabolites profiling to be performed in the target endosperm cells growing at a set temperature without any pre-treatments (Nakashima *et al.*, 2016).

In addition to SEM, TEM has long been used to conduct ultrastructural analysis in plant cells. However, using TEM to observe hard tissue, such as that found in mature rice kernels, is technically difficult, and it seems that this technique has not often been used in studies on formation of chalkiness in rice. Recently, however, Masumura’s group has established a new fixation technique suitable for TEM observation in hard kernels (Saito *et al.*, 2010, 2012). More recently, Hatakeyama *et al.* (2018) have used this fixation technique to identify the source of the air spaces that are formed along the chalky ring in dry wind-treated kernels. In this current study, we used our newly developed on-site cell-specific analysis combined with time-course TEM observations to test our hypothesis regarding the heat-induced formation of rice chalkiness, in order to identify the exact source of the air spaces formed in the cells. We found that heat decreased the rate of protein synthesis in the cells as a seed survival strategy, and consequently numerous enlarged PSVs were preserved among the loosely packed starch granules in the cytosol, which resulted in the chalky appearance. In addition, we found that N supply to the cells promoted protein synthesis and sustained the development of PBs and amyloplasts even at high temperature, thus avoiding chalkiness.

## Materials and methods

### Plant material

A growth-chamber experiment was conducted at the Kyushu Okinawa Agricultural Research Center, Chikugo, Japan in 2016, as described in previous work (Wada *et al.*, 2014). Seedlings of *Oryza sativa* L. ‘Koshihikari’ at 2 weeks old were transplanted into 3.82-l plastic pots (0.16 m diameter, 0.2 m height) containing a lowland paddy soil (Typic Endoaquepts) in June 2016. Plants were given a basal dressing of 3.5 g pot^-1^ [commercial fertilizer, 20-10-12 (N-P_2_O_5_-K_2_O)] at sowing and grown outdoors in pots (10 plants per pot, 51 pots in total) without addition of top-dressing until 4 d after heading (DAH), and tillers were periodically removed to restrict each plant to its main culm. At flowering, the pots were transferred to an environmentally controlled walk-in growth chamber with a photoperiod of 13/11 h day/night at 26/22 °C, 70/80% relative humidity, and 750 μmol photons m^−2^ s^−1^ photosynthetically active radiation (PAR) set at the plant canopy using light-emitting plasma lamps (STA 41-02, Stray Light Optical Technologies, Inc., IN, USA).

Four treatments were applied, with 11–14 pots per treatment: no heat (26 °C); N application plus no heat (N+26 °C); high temperature (34 °C); and N application plus high temperature (N+34 °C) . For the N treatment, 0.45 g pot^−1^ of urea was applied at 4 DAH. For the high-temperature treatment, pots were transferred to another growth chamber set at 34 °C and 70% relative humidity for 6 h each day (09.00−15.00) and 28 °C and 80% relative humidity for 18 h (15.00−09.00) on 5 DAH. The PAR and the photoperiod were the same as the first growth chamber (750 μmol m^−2^ s^−1^ and 13/11 h day/night, respectively), and plants were treated for 10 d. At 15 DAH, the pots were transferred back to the first chamber to grow until they reached the mature stage (40 DAH). In total there were 11 pots in the 26 °C treatment, 14 in the N+26 °C treatment, 13 pots in the 34 °C treatment, and 13 pots in the N+34 °C treatment.

### On-site cell metabolomics and turgor assays

An on-site cell metabolome analysis using picoPPESI-MS (Nakashima *et al.*, 2016) was carried out in the dorsal outer-endosperm (OE) cells of the superior kernels, i.e. attached to the primary and secondary pedicels on the first to third primary rachis branches, counted from the top of the panicle, where a high frequency of chalkiness was observed under the 34 °C treatment (see Results). Each of the two growth chambers (K260B029-S01, Tsubuku Corporation Ltd., Kurume, Japan) was attached to a measurement room in which the picoPPESI-MS system could be placed (see Supplementary Fig. S1 at *JXB* online). This arrangement allowed us to directly perform metabolite profiling of the target cells in real-time under each set environmental conditions without any significant disturbance to the plants. A pot containing plants at 11−12 DAH was placed at the center of a U-shaped vibration-free table [HOA-0808LA(Y), Herts Co. Ltd., Yokohama, Japan] in the measurement room. Because cell pressure-probe measurements are sensitive to temperature (Boyer, 1995), the probe system and plants were allowed to reach temperature equilibrium before assays were conducted (typically 30 min). Prior to an assay, a part of the hull (the palea) in the attached kernels was quickly and gently removed under humid conditions. The kernels selected for the assays had a growth score of 0.9 on a scale of 0–1 (Wada *et al.*, 2014). A 1-mm diameter biopsy punch was used to remove 0.031 cm^2^ of pericarp tissue in the dorsal side of the kernel prior to insertion of the capillary tip, so that possible contamination from the pericarp cell layers could be ruled out. The kernel was gently fixed on the sample holder using tape and magnets (Supplementary Fig. S1). With the aid of a motorized Piezo Manipulator (DC-3K, Märzhäuser Wetzlar, Germany), the tip of microcapillary, which was filled with a 0.01% (v/v) ionic liquid/silicone oil mixture (see Nakashima *et al.*, 2016), was inserted into the 2–3 OE cells below the sub-aleurone cell layer (typically between 50–150 μm below the nucellar epidermis). Cell sap was collected by depressurizing the microcapillary, and the probe tip (mounted on a 3D movement micro-manipulator) was immediately oriented toward the opening of an Orbitrap mass spectrometer (Q-Exactive, ThermoFisher Scientific Inc., MA, USA), which was charged at −4 kV using a high-voltage generator (AKTB-05k1PN/S, Touwa Keisoku Corp., Tokyo, Japan). The MS scan was performed in negative ion mode in duplicate with instrument settings of 200 ms as maximum injection time, inlet ion transfer-tube temperature of 250 °C, and resolution of 35 000. Assuming that the probe tip did not get blocked when it was inserted into the target cell, the entire process of picoPPESI-MS analysis could be completed within a few minutes. All manipulations were conducted under a digital microscope (KH-8700, HIROX Co. Ltd., Tokyo, Japan), and kernels attached to the sample holder were humidified throughout the process. The mass spectra reported here are representative of the repeated measurements from 4–5 kernels from at least three independent plants in each treatment. In addition to the picoPPESI-MS analysis, cell turgor in both the pericarp and the outer endosperm located on the same dorsal side of the kernels was independently assayed under humid conditions without removing the pericarp, as described previously (Wada *et al.*, 2011). Cell turgor values reported here represent the means of 14–29 cells from 7–8 kernels from at least three independent plants.

### Identification of cell metabolites

Exact monoisotopic *m/z* values for all the peaks on the mass spectra acquired were extracted using the Qual Browser application in the Thermo Xcalibur software (ThermoFisher Scientific). Metabolites were identified from the theoretical masses of candidate metabolites in the METLIN online metabolomics database (http://metlin.scripps.edu/index.php) allowing differences of <5 ppm. In addition to the single-cell analyses described above, MS/MS analysis was conducted on crude tissue extracts of kernels from the 34 °C treatment at 12 DAH. The kernels were frozen at –80 °C for >2 h and the pericarp tissue was gently removed. The dorsal endosperm tissue, corresponding to one-third of the whole endosperm, was removed using a razor blade, and was then freeze-dried. The tissue was then ground to a fine powder with a mixer mill (MM400, Retch, GmbH, Haan, Germany), and extracted with 50% (v/v) water/methanol. After centrifugation for 10 min at 10 000 *g* at 4 °C, the supernatant (i.e. crude tissue extract) was used for the MS/MS analysis. Collision-induced dissociation (CID) tandem MS analysis of the extract for putative metabolites was performed using the same Orbitrap MS that was coupled with the picoPPESI system, in negative ion mode. The MS scan was also performed in negative ion mode with the same instrument settings as described above, except that the resolution was 70 000. The observed MS/MS fragmentation patterns were compared against the METLIN database for GABA, proline, asparagine, asparatic acid, glutamine, glutamic acid, methionine, phenylalanine, malic acid, monodehydroascorbic acid, ascorbic acid, hexose (Hex), HexP, Hex_2_, Cl^−^ adduct ion of Hex, Cl^−^ adduct ion of Hex_2_, glutathione, and a cluster ion, cysteine (Cys)+Hex_2_. Putative Cys was also confirmed with simulated isotopic ratios using the Qual Browser application. The methanol used in the experiments was LC/MS grade and purchased from Wako Pure Chemical Industries, Ltd. (Osaka, Japan). Ultrapure water of 18.2 MΩ cm^−1^ was used throughout the experiments.

### Microscopy

Kernels for microscopic observation were sampled in the growth chambers, fixed, and embedded according to the methodology described previously by Saito *et al.* (2010, 2012) with slight modifications (Hatakeyama *et al.*, 2018). Transverse segments (1−2 mm thick) from the middle of the kernels at 12, 20, and 40 DAH were fixed with 4% (w/v) paraformaldehyde in 100 mM sodium phosphate (pH 7.2) for 3 h at room temperature and then washed in 100 mM phosphate buffer (pH 7.2). Fixed tissues were dehydrated through an ethanol series, and embedded in LR White resin in the ‘hard’ formulation (London Resin, Hampshire, UK) by polymerizing at 60 °C for 2 d. Semi-thin sections (~900 nm) for light microscopy were stained with 0.1% (w/v) Coomassie Brilliant Blue for 1 h followed by potassium iodide for 1 min, and ultra-thin sections (~80−100 nm) for electron microscopy were stained with lead citrate. After staining, the ultra-thin sections were observed with a TEM (JEM-1010, JEOL Ltd., Tokyo, Japan). Sections were cut with an ultramicrotome (Sorvall MT-5000, DuPont, Newtown, CT, USA) using a diamond knife. For the image analysis of the arrangement of organelles, the outline of all amyloplasts, PBs, and other areas (referred to as ‘gaps’) in the cells, as well as cells from light-microscope images taken from three plants, were traced using the ImageJ software (https://imagej.nih.gov/ij/), as described previously (Hatakeyama *et al.*, 2018). The areas of PSVs and both types of PBs on TEM images were similarly traced. By assuming that PSVs, PBs, and the cells were spherical, the ratio of volume (*V*=4/3π*r*^3^) to the area (*A*=π*r*^2^) was 4/3*r*, and this value was used to calculate *V* from *A*: *V*=4/3*rA*. The volume of the PSV matrix was calculated as the difference between the PSV and PBII volumes. The spatial ratio of each PB per cell was estimated. The volume of dorsal OE cells was calculated using the cell area. The number of cells in endosperm cross-sections in each treatment was counted.

### Protein extraction from rice kernels and SDS-PAGE

In each treatment, the dorsal side of the mature kernels, corresponding to the chalky zone of white-back kernels and one-third of the total kernel, was removed using a razor blade. The chalky cells in these samples in the 34 °C treatment corresponded to 69.8±13.3% of the sampled endosperm area (mean±SD, *n*=13). Total proteins from the corresponding tissues in white-back or perfect kernels in each treatment were extracted in 50 mM Tris-HCl, pH 6.8, 100 mM DTT, 8 M urea, 4% (w/v) SDS, and 10% (v/v) glycerol. Samples of extracted proteins were separated by SDS-PAGE with 10−20% acrylamide and stained with Coomassie Brilliant Blue. Three biological replicates were used.

### Kernel quality, weight, and dimensions

Spikelets in each portion of a panicle in each treatment were classified according to Matsuba (1991). The number of white-back kernels was evaluated visually for kernels >1.8 mm in thickness with 3–5 biological replicates, according to the standard evaluation method of The Ministry of Agriculture, Forestry and Fisheries of Japan (http://www.maff.go.jp/j/seisan/syoryu/kensa/pdf/genmai_kaisetsu.pdf): i.e. when the chalky portion of the dorsal side was longer than 2/3 of the kernel length and wider than 1/3 of the width, it was classified as being a white-back kernel. The fresh and dry weights of kernel samples in each treatment were determined for 3–7 biological replicates, as described previously (Wada *et al.*, 2011), and the kernel water content was calculated. The length, width, and thickness of the kernels were also measured using a digital caliper. The transversal area and the volume of the kernels were calculated as an ellipsoid. The values reported for water content and dimensions represent the means of 16–18 kernels and 18–28 kernels, respectively, collected from three independent plants.

### Nitrogen and starch content assays

The protein content of kernels pooled from every panicle in each treatment was estimated conventionally using a N/protein conversion factor of 5.95 (FAO, 2003) based on the N content as determined by the Kjeldahl method with two technical replicates. Protein contents are shown as the mean value of four biological replicates.

### Statistical analysis

Statistical analysis of all data was conducted using Tukey HSD tests in a general linear model (GLM) procedure in the JMP software (version 12.1.0, SAS Institute Inc., Cary, NC).

## Results

### Rice appearance

**Fig. 1.**
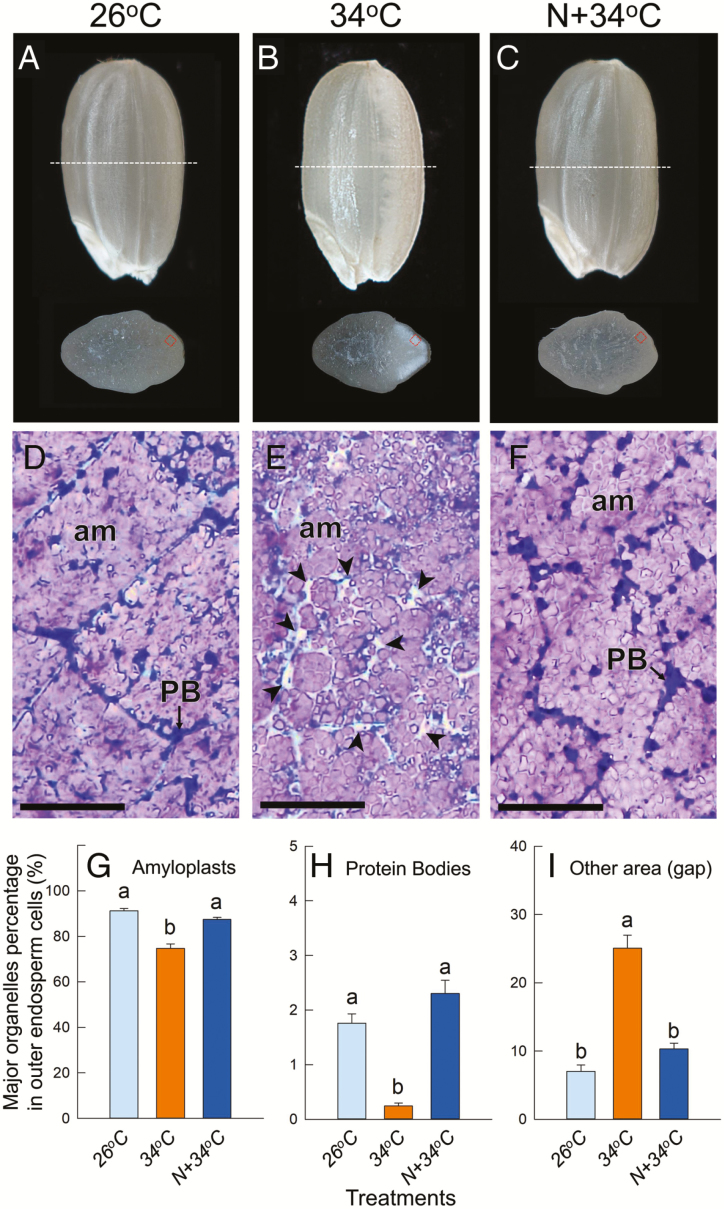
Light microscopic observations of the chalky zone of rice kernels. (A–C) Images of rice kernels and transverse sections under different heat and nitrogen treatments at 40 d after heading. (D–F) Light microscope images taken in the vicinity of the outer endosperm in the dorsal side of kernels, corresponding to the red squares on the transverse sections in (A–C). The sections were double-stained with Coomassie Brilliant Blue and iodine-potassium iodide. PB, protein body; am, amyloplast. Arrows in (E) indicate gap areas (air spaces). Scale bars are 50 μm. (G–I) Area-based percentages of (G) the major organelles, amyloplasts, (H) PBs, and (I) other areas (gaps/air spaces) in the outer-endosperm cells. The data are means (±SE) of 11–25 individual cells collected from at least three kernels from three individual plants in each treatment. Different letters indicate significant differences as determined using a Tukey–Kramer test: *P*<0.05.

In both the treatments with no heat (26 °C with or without N), there were no white-back kernels and the same proportion of perfect kernels was observed (Table 1; Fig. 1A). Under the heat treatment (34 °C) without N, the proportion of white-back kernels was 66.4%, resulting in a substantial decrease in perfect kernels (Table 1; Fig. 1B). In contrast, N application followed by heat (N+34 °C) ameliorated the effect on rice appearance and notably decreased the proportion of white-back kernels to only 7.6%, with an increase in perfect kernels, to 52% (Table 1; Fig. 1C). Partially ameliorated chalky kernels were classified as ‘other kernels’. There was no treatment effect on kernel dry weight between the perfect and the white-back kernels (Table 1), although the mean dry weight of all kernels pooled together declined in the 34 °C and N+34 °C treatments. Given that no differences in rice appearance were observed between the 26 °C and N+26 °C treatments, subsequent experiments were conducted using just the 26 °C, 34 °C, and N+34 °C treatments. In terms of dimensions, there was a slight decline in the width of white-back kernels in the 34 °C and N+34 °C treatments relative to perfect kernels at 26 °C, but no differences were observed for length and thickness (Supplementary Table S1). As a result, there were also no differences in the calculated transversal area and volume. In the white-back kernels in the 34 °C treatment, the chalky area corresponded to 17.4±0.8% (mean±SD, *n*=18) of the transverse section (Fig. 1B).

**Table 1.** Rice appearance and individual kernel weight

Treatment	Filled kernels (%)	Appearance (%)			Kernel dry weight (mg)			
		Perfectkernels	White-backkernels	Other kernels	Perfectkernels	White-backkernels	Other kernels	Mean
26 °C	97.9	100.0^a^	0.0^b^	0.0^a^	21.6	-	-	21.7^a^
N+26 °C	96.5	98.9^a^	0.0^b^	1.1^a^	21.8	-	22.7^a^	21.8^a^
34 °C	94.4	11.4^c^	66.4^a^	22.2^a^	19.9	20.2	20.2^b^	20.2^b^
N+34 °C	94.7	52.1^b^	7.6^b^	40.4^b^	20.2	20.7	20.0^b^	20.2^b^

Appearance: means measured from superior kernels attached to the upper position of a panicle, 3–5 panicles per treatment. Kernel dry weight: means of kernels collected from 3–7 panicles per treatment. Different letters indicate significant differences as determined using a Tukey–Kramer test: *P*<0.05.

### Microscopic observations in the chalky zone

**Fig. 2.**
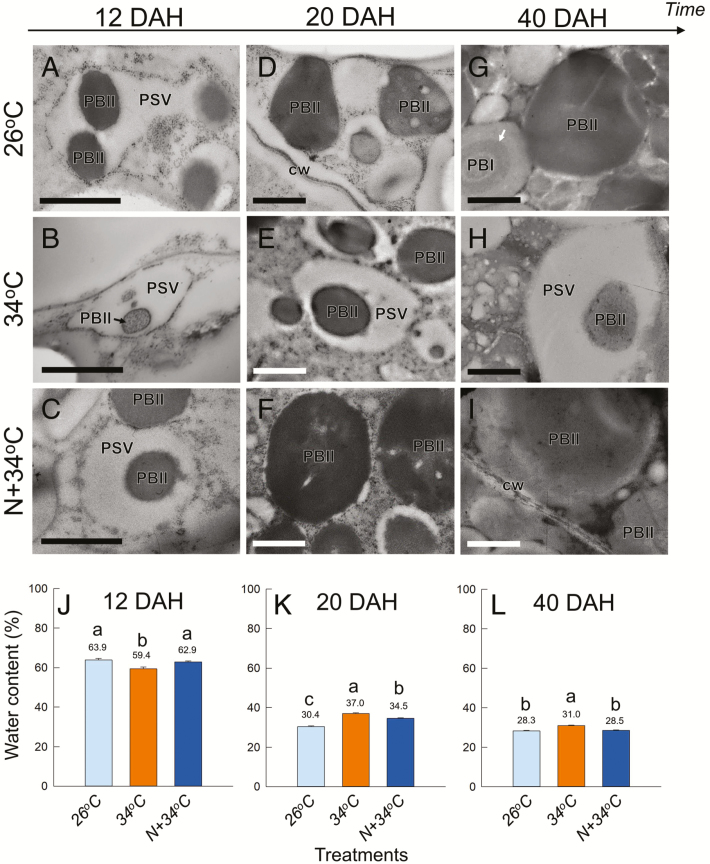
Changes in the ultrastructure of protein bodies and in water content of rice kernels. (A–I) TEM images of outer-endosperm cells under different heat and nitrogen treatments at 12–40 d after heading (DAH). PSV, protein-storage vacuole; PBI, protein body type I; PBII, protein body type II; cw, cell wall. The arrow in (G) indicates the Cys-rich 10-kDa layer formed in PBIs. Scale bars are 1 μm. (J–L) Water content of kernels in each treatment at the same three ripening stages. Data are means (±SE) of 16–18 kernels collected from three plants; the mean values are labelled above the bars. Different letters indicate significant differences as determined using a Tukey–Kramer test: *P*<0.05.

The OE cells located at the dorsal side of the grains, where the highest frequency of chalkiness was found at maturation, were observed using a light microscope and TEM. Grain sections collected from the 26 °C, 34 °C, and N+34 °C treatments were dual-stained and observed using a light microscopy, and starch granules and proteins were stained as reddish-purple and blue, respectively (Fig. 1D–F). Essentially, there were no treatment differences in the number of cells in the endosperm cross-sections, although the volume of OE cells located in the chalky zone decreased in both the heated treatments relative to 26 °C (Supplementary Table S2). In the 26 °C treatment, the OE cells were densely packed and generally had numerous, well-developed amyloplasts and mature PBs that were mostly located at the periphery of cytoplasmic compartments (Fig. 1D). In contrast, the size of amyloplasts observed in the 34 °C treatment was more heterogeneous and there were relatively large air spaces in the cytoplasmic compartments (Fig. 1E, arrowheads). The cell morphology in the N+34 °C treatment was similar to that in at 26 °C (Fig. 1F). Image analysis showed that there were significantly reduced areas of amyloplasts and PBs in the cytoplasmic compartments in the 34 °C treatment (Fig. 1G, H), resulting in air spaces representing over 25% of the chalky zone (Fig. 1I). In contrast, in the N+34 °C treatment the percentage area of amyloplasts and PBs was similar to the level in the 26 °C treatment, indicating that the heat-induced modifications of organelle compartmentation were ameliorated by additional N supply (Fig. 1G–I). Observations using TEM were then made in the same zone for each treatment at 12, 20, and 40 DAH (Fig. 2A–I). At 12 DAH, similarly developing PSVs were observed in all treatments (Fig. 2A–C). Numerous PSVs, a few PBs, mitochondria, and rERs were also observed in the cytosol. In contrast, at 20 and 40 DAH the ultrastructure of the cells differed between treatments. The majority of PSVs were filled with PBIIs at 20 DAH in the 26 °C treatment (Fig. 2D), whereas PSVs in the 34 °C treatment remained in the cytosol, leading to the formation of air spaces at maturation (Fig. 2E, H). In the chalky zone in the 34 °C treatment, a small number of cells located adjacent to the sub-aleurone layer, corresponding to 3.4% of the total chalky area (the red area in Supplementary Fig. S2A), retained some large lytic vacuoles in addition to PSVs at 20 DAH (Supplementary Fig. S2B). In contrast, normal PBII development was observed in the N+34 °C treatment with few or no PSVs at 40 DAH, similar to the 26 °C treatment (Fig. 2I). In each treatment, the water content of the kernels decreased through the grain-filling stage, although at maturation higher water content was observed in the 34 °C treatment (Fig. 2J–L).

### Assaying turgor in individual cells

**Fig. 3.**
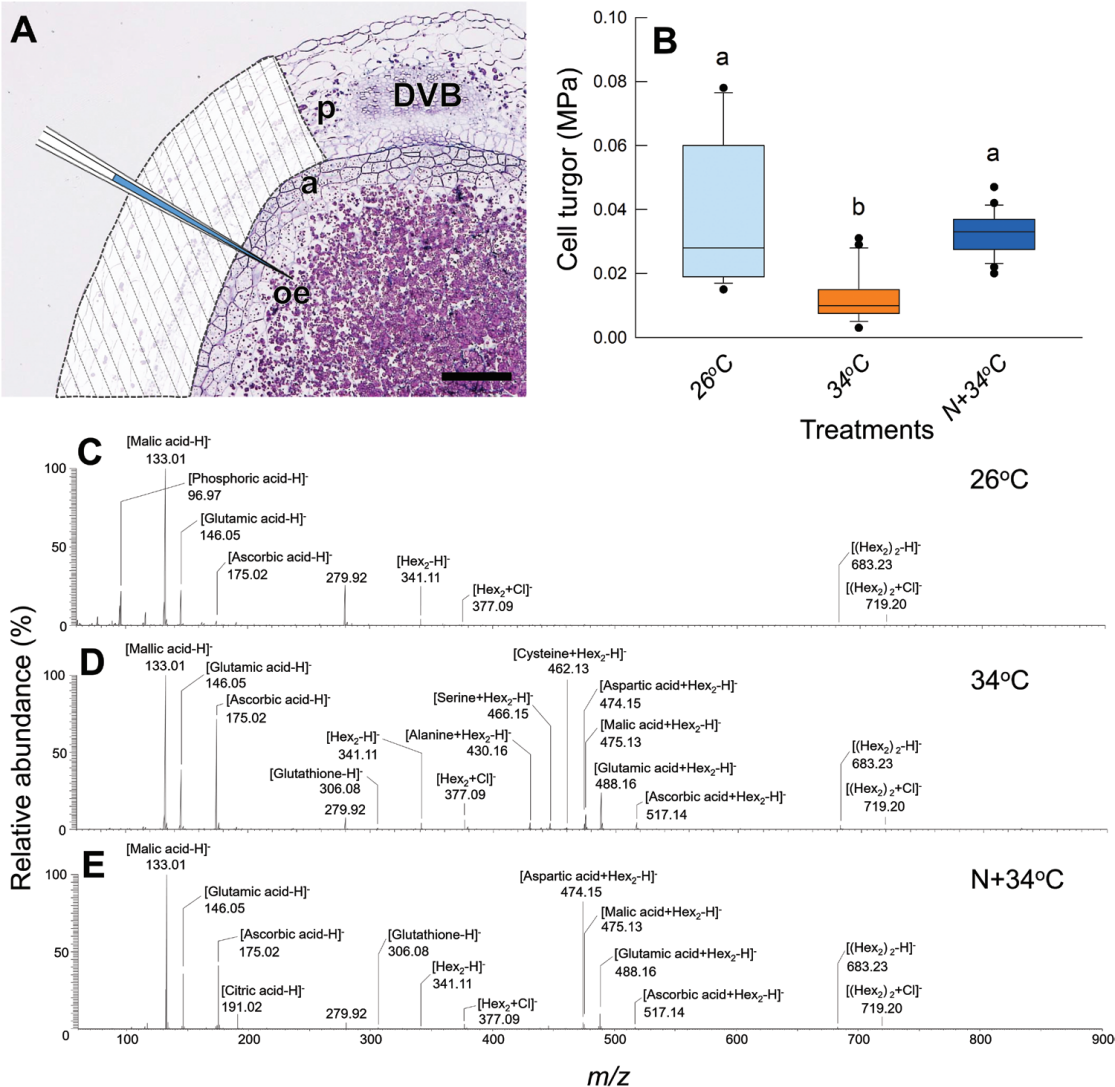
Cell turgor and metabolite profiling in the putative chalky zone of rice kernels. (A) Image of a transverse section of the dorsal side of a kernel at the early ripening stage (12 d after heading, DAH) including a diagram of the pressure probe used for extraction of sap from the outer endosperm cells. A part of the pericarp, indicated by the shaded area, was removed prior to the insertion of the probe tip (see Methods). DVB, dorsal vascular bundle; a, aleurone layer; oe, outer endosperm; p, pericarp. The scale bar is 100 µm. (B) Box-plot showing the cell turgor in the outer endosperm in each heat/nitrogen treatment assayed at 12 DAH. Data are from 14–29 cells from 7–8 kernels from at least three different plants in each treatment and show the mean, the upper and lower quartiles, the maximum and minimum values, and outliers (dots). Different letters indicate significant differences as determined using a Tukey–Kramer test: *P*<0.05. (C–E) Mass spectra for picoPPESI-MS in negative ion mode obtained from the outer-endosperm cells for the different treatments at 12 DAH. The data are representative of repeated experiments with 4–5 kernels in each treatment.

In individual cells at the early stage of kernel development (12 DAH), when PSVs were similarly localized in all the treatments (Fig. 2A–C), variation in the turgor of OE cells could be observed between treatments (Fig. 3B). Cells in the 34 °C treatment exhibited relatively low turgor compared to those in 26 °C treatment. Cell turgor in the N+34 °C treatment was higher than that in the 34 °C treatment, and was at the same level as the 26 °C treatment.

### Identification of metabolites in individual cells

In a preliminary experiment, it was observed that there were distinct tissue-to-tissue variations in metabolites between the pericarp (Supplementary Fig. S3) and OE cells (Fig. 3C–E). To avoid a possible contamination, a part of the pericarp tissue in the dorsal side of the kernel was removed prior to insertion of the capillary tip into the target endosperm cells (Fig. 3A) (see Methods). When the cell sap was directly analysed using picoPPESI-MS under controlled environments (Supplementary Fig. S1), numerous metabolites (mostly amino acids, sugars, organic acids, and secondary metabolites) were identified in negative ion mode with <5 ppm differences from theoretical molecular weight values (Fig. 3C–E, Supplementary Table S3, Supplementary Fig. S4). In the 26 °C treatment, the peaks of phosphoric, malic, and glutamic acids (as [M−H]^−^, M=molecular species), and some sugars (as [M−H]^−^ and/or [M+Cl]^−^) in the mass spectrum identified them as major ions. In the 34 °C treatment, the signal intensities of sugars and amino acids including glutamic acid were generally higher than in the 26 °C treatment. The signal intensities of ascorbic acid and glutathione, both of which are involved in the scavenging of reactive oxygen species (ROS), were higher in the 34 °C treatment than at 26 °C, as would be expected in a typical heat response (Fig. 3D; Supplementary Table S3). Greater accumulation of Cys, which forms disulfide bridges, was observed in the 34 °C treatment (>75% frequency), whereas no Cys-related signals were detected at 26 °C (Supplementary Table S3). In the N+34 °C treatment, the signal for Cys (as [Cys−H]^−^) was low and rarely observed (<20% frequency), consistent with signals for Cys-sugar cluster ions, such as [Cys+Hex−H]^−^ and [Cys+Hex_2_−H]^−^ (not detected and 0.09%, respectively). For methionine (Met), the signal intensities of [Met−H]^−^ and two Met-sugar cluster ions, [Met+Hex−H]^−^ and [Met+Hex_2_−H]^−^, were smaller than those of Cys, with no clear differences in the different treatments.

### Effects of heat and enhanced N on protein body development

**Fig. 4.**
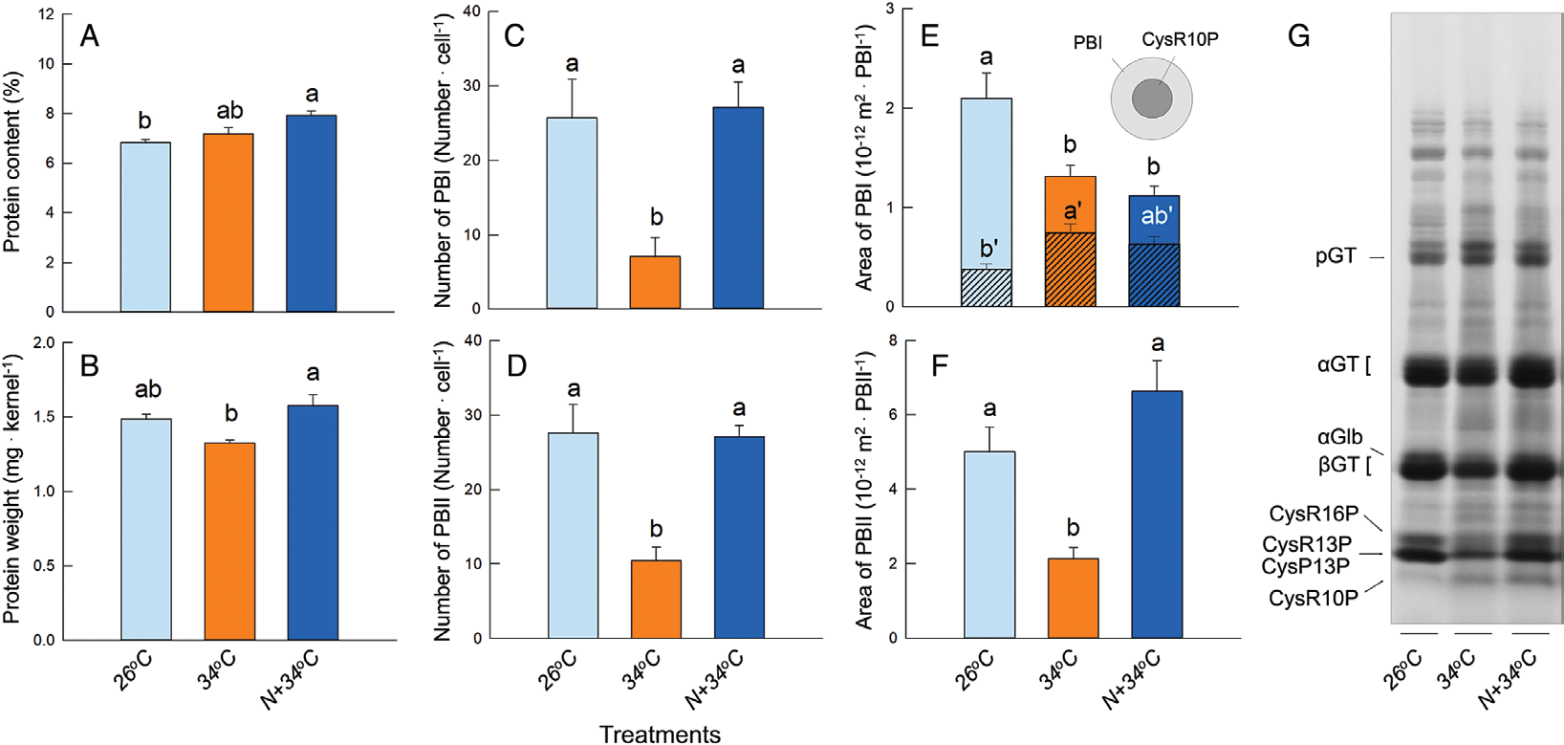
Results of image analysis of protein bodies in the chalky zone of rice kernels. (A) Protein content of kernels pooled in each treatment at 40 d after heading (DAH). (B) Protein weight of kernels in each treatment at 40 DAH. Data in (A, B) are means (±SE) for four plants. (C, D) The number of PBIs and PBIIs in the outer-endosperm cells. (E, F) Cross-section areas of PBIs and PBIIs in the same cells. Data in (C–F) are means (±SE) for 25–59 PBs from at least three kernels. The inset diagram in (E) shows the Cys-rich 10-kDa layer located at the center of the PBIs and corresponds to the hatched areas on the graph. Different letters in (A–F) indicate significant differences as determined using Tukey–Kramer tests (*P*<0.05): in (E) differences in the area of the Cys-10-kDa layer are indicated with ´. (G) SDS-PAGE analysis of one-third of dorsal side of the kernels, corresponding to the chalky zone. CysR10P, Cys-rich 10-kDa prolamin; CysP13P, Cys-poor 13-kDa prolamin; CysR13P, Cys-rich 13-kDa prolamin; CysR16P, Cys-rich 16-kDa prolamin; βGT, glutelin basic subunit; αGlb, α-globulin; αGT, glutelin acidic subunit; pGT, proglutelin.

**Fig. 5.**
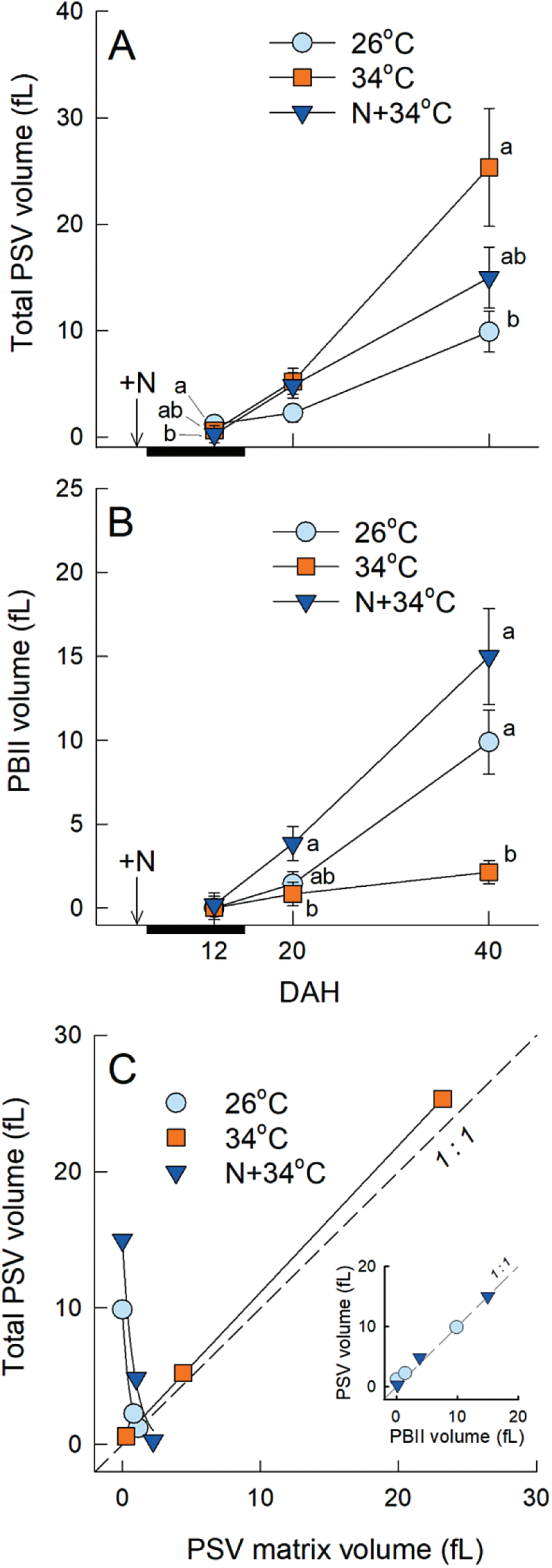
Changes in protein storage vacuoles (PSVs) and PBIIs during rice kernel development. (A) Time-course of changes in the estimated PSV volume in each heat/nitrogen treatment. (B) Time-course of changes in the estimated PBII volume in each treatment. The time of N application at 4 d after heading (DAH) is indicated and the period of 34 °C treatment is also indicated (black bar, 5–15 DAH). The data are means (±SE) from areas of 4–38 PSVs or PBIIs. Different letters indicate significant differences as determined using a Tukey–Kramer test: *P*<0.05. (C) PSV volume as a function of the corresponding PSV matrix volume. The regression line between the PSV matrix volume (*x*) and the total PSV volume (*y*) in the 34 °C treatment is *y*=1.08*x*+0.41, with *r*^2^=0.99 (*P*<0.01). The inset shows the relationship between PSV volume (*y*) and PBII volume (*x*) in the 26 °C and N+34 °C treatments: the regression line for 26 °C is *y*=0.89*x*, with *r*^2^=0.99 (*P*<0.01), and for N+34 °C it is *y*=0.98*x*+0.01, with *r*^2^=0.99 (*P*<0.05).

When exposed to heat, the protein content of the pooled kernels tended to increase (Fig. 4A) due to the reduction in final kernel weight (Table 1). However, the pooled protein weight per kernel in the N+34 °C treatment was similar to that at 26 °C (Fig. 4B). The number of PBs and their individual areas decreased considerably in the 34 °C treatment (Fig. 4C–F). Application of N increased the numbers of each PB and the area of PBIIs, but not the area of PBIs (Fig. 4C–F). The area of PBIIs in the 26 °C treatment was ~2.5-fold greater than that of PBIs localized in the cells (Fig. 4E, F). PBIs are composed of two types of layers, namely an inner layer of Cys-rich 10-kDa prolamins (CysR10P) and an outer layer containing a mixture of other prolamins (Fig. 2G). In the 34 °C treatment, the apparent area of CysR10P in the PBIs increased, although the outer layer was decreased considerably (hatched bars in Fig. 4E). In the N+34 °C treatment, the area of CysR10P in the PBIs was similar to that in the 34 °C treatment and tended to be higher than at 26 °C. Consequently, the area of CysR10P (number × area) in the chalky cells increased as a result of N application compared to that in the 34 °C treatment (Supplementary Fig. S5A). A similar observation was made for the spatial ratio of PBIs and PBIIs in the cells (Supplementary Fig. S5C, D). There was little effect on the accumulation of glutelin precursors (pro-glutelin) and acidic (α)-glutelin at 34 °C, but the contents of CysR16P, 13-kDa prolamins, α-globulin, and basic (β)-glutelin decreased in the 34 °C treatment (Fig. 4G). When N was supplied to the soil prior to 34 °C treatment, the contents of β-glutelin and CysR16P increased to the same level as the 26 °C treatment and greater than that of the 34 °C treatment. Accumulation of CysR10P was specifically observed in the 34 °C treatments. Analysis of the time-course of changes of the volumes of PSVs and PBIIs (which are stored in PSVs) in the chalky zone showed that the expansion of PSVs occurred progressively in the 34 °C treatment, reaching 26 fL at maturation, 2.5-fold larger than that in 26 °C treatment (Fig. 5A). The accumulation rate of PBIIs was much lower in the 34 °C treatment compared to 26 °C (Fig. 5B). In the 26 °C and N+34 °C treatments there were positive relationships between the total volumes of the PSVs and the PBIIs (inset in Fig. 5C). In contrast, in the 34 °C treatment the PSV volume was positively correlated with the PSV matrix volume (Fig. 5C).

## Discussion

Heat stress induces the formation of chalkiness in part of the rice endosperm (Hoshikawa, 1989; Tashiro and Wardlaw, 1991), whilst N supply reduces chalkiness even at high temperature (Wakamatsu *et al.*, 2008). The regulation of the rate of protein synthesis is predicted to be associated with the extent of formation of chalkiness through a disruption of both amyloplast and PB development, although the exact metabolic changes occurring under heat conditions in terms of the structural modifications have not been directly determined. In this study, we examined the cellular dynamics of heat-induced formation of chalkiness and the mitigating effects of N by employing a newly developed cell metabolomics system (Supplementary Fig. S1) and time-course TEM analysis, in conjunction with a previously developed fixation technique (Saito *et al.*, 2010, 2012). Our data provide compelling evidence that the formation of enlarged PSVs caused by a reduction in the rate of synthesis of storage proteins is responsible for the creation of air spaces in the chalky zone observed in mature kernels.

### Formation of chalkiness associated with the N level under heat conditions

**Fig. 6.**
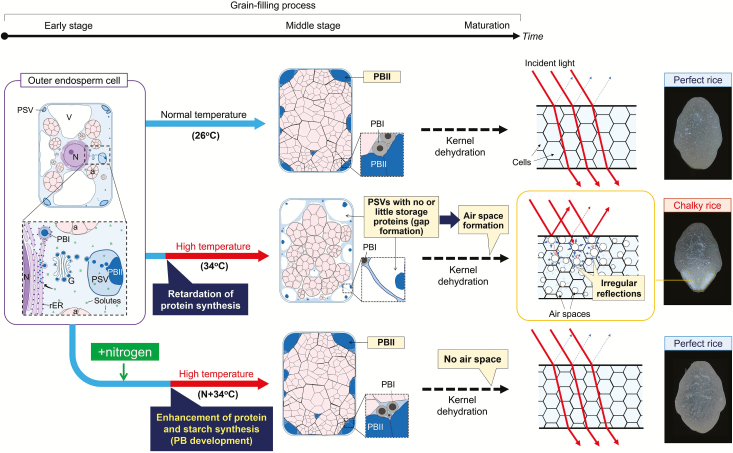
A diagram illustrating the proposed process of heat-induced formation of chalkiness and nitrogen-enhanced adaptation in rice kernels. Under normal conditions (26 °C treatment), as the packing density of amyloplasts increases in the developing outer-endosperm cells, there is a reduction in the volume of the cytosol, which is mainly occupied with vacuoles (V), resulting in adequate development of amyloplasts and protein bodies (PBs) that diminish the cytosolic space (Supplementary Fig. S2C, F, I). If the N level is low prior to high temperature (34 °C treatment), the osmotic adjustment in the cells is less and the rate of protein synthesis is reduced to preserve protein-storage vacuoles (PSVs) and vacuoles, with a partial reduction in starch metabolism (see Discussion), resulting in formation of air spaces during kernel dehydration. Due to the irregular reflection caused by the air spaces, light is scattered and does not pass through the kernel, which leads to loss transparency and hence a chalky appearance. When N supply is adequate prior to high temperature (N+34 °C treatment), the cells maintain the rate of protein synthesis by strong osmotic adjustment and maintain normal PB and amyloplast development, which reduces the presence of air spaces, thus resulting in a substantial reduction in chalky kernels.

The likely processes involved in the formation of chalkiness and the the mitigating effects of N that occur in heat-treated cells is shown in Fig. 6. The TEM image analysis suggested that the volume of PSVs increased dramatically, reaching up to 2.5-fold greater than PBIIs in the 26 °C treatment at maturation (Fig. 5A) but with ~16.5% reduction in starch accumulation (Figs 1G, 6). When N was supplied prior to the 34 °C treatment, the percentage area of amyloplasts and PBs in the cells recovered to a similar level as that in the 26 °C treatment (Fig. 1G, 1H), indicating that the synthesis of proteins, mainly starch biosynthesis-related enzymes and disulfide-rich storage proteins, was sustained in the cells (Figs 4, 5, Supplementary Fig. S5). As a result, the N-enhanced cellular adaptation to heat reduced the spatial ratio of the air spaces to be similar to that in the 26 °C treatment, 14.8% lower than that of the white-back kernels harvested in the 34 °C treatment (Fig. 1I). The data showed that ~70% [*∆*perfect kernel 40.7% (=52.1–11.4%) / *∆*white-back kernel 58.8% (=66.4–7.6%)] of what would have been white-back kernels instead formed perfect kernels without there being any reduction in weight (Table 1). Given that the activities of starch metabolism-related enzymes would have been under redox regulation (Kötting *et al.*, 2010), these data indicate that the amount of N applied was sufficient to ameliorate the cellular redox conditions under the heat stress and to sustain protein synthesis, leading to normal amyloplast and PB development to form perfect kernels.

In this study, there was no effect of heat on the total number of endosperm cells observed in the transverse sections, which was different to a previous report by Morita *et al.* (2005), probably due to differences in treatments and cultivars used. Although there were no differences in total kernel volume between treatments (Supplementary Table S1), a reduction in cell volume was observed locally in the dorsal zone that we targeted (Supplementary Table S2), consistent with previous work (Morita *et al.*, 2005). The numerous air spaces that formed in these cells would cause irregular reflection and scattering of light, thereby causing the chalky appearance (Fig. 6). It has recently been demonstrated that the formation of vacuole-like structures among inadequately accumulated amyloplasts in the endosperm cells of rice are the main cause of chalky ring formation in plants exposed to the hot, dry winds (Hatakeyama *et al.*, 2018). The loss of transparency similarly observed in the dorsal side of kernel in this study (Fig. 1B) could thus largely depend on the number and volume of vacuole-like structures, including PSVs, present in the heat-treated cells at maturation.

### Heat stress and PB morphology

Microscopic observations clearly showed that heat stress significantly affected PB development in the rice endosperms, and that this depended on the level of N (Fig. 2A–I). Assuming that there were no differences between treatments for the number of PSVs originally formed in the cells, the mean ratio of PSVs to the estimated air-space area (i.e. cytosol) in the 34 °C treatment was 24.5% (ranging between 6.5−46.4%), based on the number of PBIIs (Fig. 4D) and PSV volume (Fig. 5A) determined in individual cells. This indicated that the PSVs were the major organelle occupying the cytosol at maturation under heat conditions, apart from the amyloplasts. Hence, we propose that the numerous PSVs present in the cytosol with little accumulation of disulfide-rich PBs (Figs 2E, H and 5) may participate in the formation of air spaces under heat conditions, as well as being responsible for the partial decline in starch accumulation. Most mutants with abnormal PBII formation show floury endosperms with an opaque appearance (Takemoto *et al.*, 2002; Wang *et al.*, 2010; Fukuda *et al.*, 2011; Li *et al.*, 2014; Ren *et al.*, 2014), whereas in contrast a knockdown mutant of the vacuolar-processing enzyme *OsVPE1* produces translucent kernels (Wang *et al.*, 2009). OsVPE1 plays a role in the maturation of glutelin transported into PSVs as Cys protease in the final stage of PB development (Wang *et al.*, 2009). The microscopic observations provided by Wang *et al.* (2009) indicate that the mutant exhibits numerous round PBIIs with little or no PSV matrix in the developing endosperms prior to the formation of translucent kernels (see their fig. 2), which is in contrast to the PB morphology that we observed in the 34 °C treatment (Fig. 2E, H). Considering the significant differences in PB morphology, it is not surprising that VPE does not have a dramatic impact on the extent of chalkiness. Thus, the results of Wang *et al.* (2009) do not necessarily conflict with our results.

### On-site cell-specific analysis in rice endosperms

Cell metabolomics has been widely extended due to the introduction of the Orbitrap mass analyser, and this approach has been applied to a number of biological studies including plant cells (Gholipour *et al.*, 2013; Fujii *et al.*, 2015; Nakashima *et al.*, 2016). In our new on-site cell metabolomics system that can be conducted in controlled environments (Supplementary Fig. S1), both the growing plants and the analytical environment could be equilibrated at the same set temperature. Under these temperature equilibrium conditions, a probe tip was accurately inserted into the putative chalky zone in kernels still attached to the plants. The picolitre volumes of cellular fluids that were collected in the quartz capillary could immediately be subjected to metabolome analysis without any further treatment (see Methods). The use of this method at the early ripening stage allowed us to determine treatment effects in cell turgor and metabolites in the developing dorsal outer-endosperm (OE) cells (Fig. 3), where numerous PBs are known to be localized (Ellis *et al.*, 1987; Hoshikawa, 1989). We found that there were no obvious treatment differences in PB morphology at 12 DAH (Fig. 2A–C). This study also highlighted the importance of highly selective and precise cell-sap extraction (and subsequent MS analysis) that can be conducted using the picoPPESI-MS system. Although only limited data were obtained from pericarp cells at the stage examined (Supplementary Fig. S3), very clear treatment differences in cell metabolites could be observed in the dorsal OE cells, as discussed below.

### Osmotic adjustment under heat conditions

In general, osmotic adjustment occurs in growing cells at moderately low water potential (Morgan, 1977; Meyer and Boyer, 1981). During osmotic adjustment, turgor pressure can be maintained by accumulating osmotically active solutes, such as sugars and amino acids, into the cells (Morgan, 1977; Meyer and Boyer, 1981). As reported previously (Wada *et al.*, 2011, 2014), osmotic adjustment also occurs in the endosperm cells of rice growing under dry wind conditions prior to chalky ring formation. Hatakeyama *et al.* (2018) have proposed that the appearance of vacuole-like structures occurs as a consequence of osmotic adjustment in the inner endosperm under such wind-induced low water potential. In our current work, on-site cell metabolomics and cell turgor assays in the outer endosperm at the early ripening stage indicated notable treatment differences, including in redox metabolites, such as Cys, ascorbic acid, and glutathione, as well as differences in cell turgor as heat adaption responses (Fig. 3, Supplementary Table S3).

Cys stabilizes the tertiary structure of proteins through the formation of disulfide bonds, which is necessary for protein folding and the modulation of enzyme activity in cells. In rice endosperms, there is active synthesis of large amounts of disulfide-rich storage proteins, consuming cytosolic Cys under normal, no-stress conditions. However, this situation may be altered under certain heat conditions. A greater abundance of Cys-related signals was frequently observed in the heat-treated cells, but these signals were rarely observed in the other treatments (see [Cys−H]^−^, [Cys+Hex−H]^−^, and [Cys+Hex_2_−H]^−^ in Fig. 3 and Supplementary Table S3). In additional experiments using stable isotopes, we consistently observed similar patterns of solute accumulation in the same cells associated with changes in PB morphology (Y. Hatakeyama *et al.*, unpublished results). These data suggest that in the 34 °C treatment a relatively high concentration of Cys would have accumulated in the cytosol as an osmotically active solute under long-term heat conditions. It is notable that the substantial solute accumulation (including amino acids and sugars) preceded the inhibition of PB development, which was observed during late ripening stage in the 34 °C treatment, as discussed above (Fig. 2B, E, H).

In contrast, we suggest that during the N-enhanced adaptation process at high temperature, Cys would have contributed to vacuolar trafficking and protein processing without participating in osmotic adjustment. It also appeared that N application promoted amyloplast development under heat conditions, as well as PB formation (Fig. 1G). Hence, it should be noted that the effects of the N that we supplied were large enough to structurally stabilize the activity of a series of starch biosynthesis-related enzymes under heat conditions, together with maintaining PB development. The observed N-induced increase in Cys-rich 10-kDa prolamin in PBIs (Fig. 4E) and the results of SDS-PAGE analysis (Fig. 4G) strongly support this conclusion. There seem to be few studies of osmotic adjustment under heat conditions as compared to those examining water stress. In our study, direct determination of cell osmotic pressure using a freezing-point osmometer was not possible in the kernels at 11−12 DAH that we examined because of the interference of numerous starch granules contained in the sap. However, based on the strikingly different cell responses to heat that we detected in the examined cells (Fig. 3C–E, Supplementary Table S3) and the time-course of changes in PB morphology (Fig. 5) discussed above, it is a reasonable interpretation that N-treated cells would show strong osmotic adjustment and thus maintain high turgor to sustain the protein synthesis rate even at high temperature. In contrast, under low N levels, the cells would show less osmotic adjustment at low turgor and the protein synthesis rate would slow down, so that the energy requirement in the cytosol could be kept low.

### Endosperm N budget

It remains questionable as to how much N would have been synthesized into the storage proteins during the adaptation process. Analysis of our TEM images comparing the 34 °C and N+34 °C treatments indicated that the mean differences in the spatial ratios of PBIs and PBIIs in the cells were 0.52% and 3.86%, respectively (Supplementary Fig. S5C, D). Given the proportion of the chalky area in the transverse sections (17.4%), the kernel dimensions (mean transversal area and kernel length) (Supplementary Table S1), and the specific gravity of PBIs and PBIIs (1.27 and 1.29 g ml^−1^, respectively; Tanaka *et al.*, 1980), the weight difference between the chalky zones in the two treatments corresponds to 0.17 mg. Because the treatment difference in the protein content per kernel was 0.15 mg (Fig. 4B) and the difference in the occurrence of white-back kernels was 58.8% (Table 1), our estimation suggests that in the N+34 °C treatment ~65.8% of the increase in proteins resulting from N application were synthesized as storage proteins in the PBs to fill up the air spaces in the chalky zone. And, the remaining 34.2% of the proteins would have been used for synthesis of PBs in other areas and for other enzymes, mostly those related to starch synthesis. Thus, we suggest that the spatial contribution of the morphological changes in PBs to preventing air spaces may be greater than the effect of the size reduction in amyloplast development, at least in the white-back rice examined here, which is different to other types of chalky rice.

### Formation of chalkiness as a form of heat acclimation

There is a large body of evidence that reactive oxygen species, such as hydrogen peroxide, play an important role as second messengers in signal-transduction networks associated with developmental processes or in response to abiotic stress (Mittler *et al.*, 2004; Skopelitis *et al.*, 2006). Hydrogen peroxide might serve as a signal that induces programmed cell death and subsequent kernel desiccation (Onda *et al.*, 2009). It has been suggested that both membrane shrinkage and degeneration of PBs occurs before PB development is completed in barley endosperm cells (Ibl *et al.*, 2014). The synthesis of large amounts of disulfide-rich storage proteins during grain-filling (Fig. 4E, G) might be accompanied by the production of hydrogen peroxide in the ER, resulting in the peroxidation of membrane lipids under normal conditions (Sattler *et al.*, 2006; Onda *et al.*, 2009). In contrast, the behavior of PSVs (Figs 2H, 5) and the results of cell metabolomics (Fig. 3, Supplementary Table S3) obtained in our study both suggested that heat might have disturbed the cellular redox status to inhibit the peroxidation of membrane lipids. PSVs treated at 34 °C were found to be expanding over time, but with a reduction in PB accumulation (Fig. 5A, B). Importantly, the increase in PSV volume could be explained by an increase in the PSV matrix (Fig. 5C), indicating that substantial amounts of water had been entering PSVs as they matured to increase the vacuolar volume. Therefore, it is quite unlikely that tonoplast membrane lipids were degraded under heat conditions.

One plausible explanation is that a partial degradation of PBIIs in PSVs would occur through the activation of proteases or through an autophagy-like process, leading to an increase in the vacuolar osmotic pressure. This would promote water entry into the PSVs to sustain the vacuolar volume by maintenance of cell turgor, which would sustain kernel growth, as we observed (see filled kernels in Table 1 and kernel volume in Supplementary Table S1). The source of the water accumulated in the PSV matrix remains unknown. However, based on the increases in the contents of antioxidant metabolites, ascorbic acid, glutathione, and monodehydroascorbic acid detected at the cellular level (Supplementary Table S3), we speculate that the accumulation might be a consequence of an increased activity of ascorbate peroxidase catalysing the conversion of hydrogen peroxide into water. The fact that PB development was maintained during the N-enhanced adaptation process suggests that disulfide bond formation and tonoplast denaturation would both be facilitated by strong osmotic adjustment. Kernels in the 34 °C treatment exhibited higher water content than at 26 °C (Fig. 2J-L), consistent with previous studies (Ishimaru *et al.*, 2009). Given that water is a major constituent of both the PSV matrix and vacuoles in the gap spaces, it is not surprising that the chalky zone (or the whole chalky grain) exhibited relatively high moisture content under heat conditions, compared to the 26 °C treatment. Storing water in the endosperm along the dorsal vasculature may be an essential event to sustain embryo development in rice seeds exposed to the extremely high temperature, as heat-induced precocious germination is known in oilseed rape (Brunel-Muguet *et al.*, 2015). From the physiological point of view, we propose that the formation of chalkiness in rice can be regarded as a form of heat acclimation rather than being abnormal endosperm development, which it has long been considered to be (Tashiro and Wardlaw, 1991).

### Threshold of endosperm loss of transparency

Regarding the area threshold above which chalkiness appears, our data suggest that the transition corresponded to a range between 10.3% and 25.1% in the N+34 °C and 34 °C treatments, respectively (Fig. 1I). This compares with a mean value of 13.3% observed using SEM in another type of chalkiness, termed milky-white rice (Wada *et al.*, 2014). Taken together, a significant loss of transparency is likely to occur in a range of 10.3% to 13.3%, which would partially depend on the observation methods used. In the case of the leading rice cultivar ‘Koshihikari’ that we examined, it is notable that vacuoles were also observed in a small portion of the chalky zone, together with PSVs (Supplementary Fig. S2B). This indicates that some vacuoles would also have participated in the formation of air spaces, as has been observed in chalky ring formation induced by dry wind (Hatakeyama *et al.*, 2018). In addition, a clear varietal difference in the occurrence of white-back kernels has been reported when the same amount of N was applied under heat conditions (Wakamatsu *et al.*, 2008), although the underlying cellular mechanisms remain unexplained. Since the findings reported by Wakamatsu *et al.* (2008) were also obtained in ‘Koshihikari’, there may be some specific variety × site interactions in terms of the cellular heat responses in the kernels. Morphological differences, such as vacuolar and PB morphology, may also explain varietal differences; however, this requires further studies.

## Conclusions

In this study, we identified treatment differences in heat-induced cellular responses in metabolite composition and cell turgor in rice kernels, which subsequently induced spatial changes in cell structure, including PB morphology. Our data indicated that heat disrupted protein synthesis to inhibit PB formation, accompanied by a partial inhibition of amyloplast development, leading to the appearance of chalkiness. In contrast, cells in plants supplied with N maintained PB and amyloplast development, allowing them to suppress the formation of chalkiness even at high temperature. Hence, we conclude that preservation of large cytosolic PSVs and inadequate amyloplast accumulation are both responsible for the formation of air spaces that occurs in the dorsal outer-endosperm in heat-induced chalky grains. Our results also highlight the important role that regulation of the rate of protein synthesis plays in optimizing organelle compartmentation in rice endosperms under heat stress. It is now possible to trace metabolites in specific zones using stable isotopes in mass spectrometry (Wada *et al.*, 2017). The use of cell-specific analysis combined with the isotope feeding will further extend our understanding of heat adaptation mechanisms in rice endosperms. Finally, our on-site cell metabolomics approach using picoPPESI-MS has the potential to be applied across many cell-specific studies in environmental plant biology as a powerful analytical method.

## Supplementary data

Supplementary data are available at *JXB* online.

Fig. S1. Diagram of the on-site cell metabolomics system.

Fig. S2. Morphological changes in the dorsal outer-endosperm during kernel development.

Fig. S3. Mass spectra of cell metabolites detected using picoPPESI-MS in pericarp cells in at the early stage of kernel development.

Fig. S4. MS/MS analysis and isotope analysis performed using an Orbitrap mass spectrometer coupled with the picoPPESI system in negative ion mode.

Fig. S5. Areas and spatial ratios of PBIs and PBIIs in outer-endosperm cells in kernels at 40 DAH.

Table S1. Dimensions of representative kernel types in each treatment.

Table S2. Effects of temperature and N application on the number and size of endosperm cells.

Table S3. List of metabolites detected using picoPPESI-MS in outer-endosperm cells.

Supplementary Figures S1-S3, S5 and_Tables S1-S3

Supplementary Figures S4
