# Supplementary material for: Multiple strategies for heat adaptation to prevent chalkiness in the rice endosperm
Source: J Exp Bot. 2018 Dec 3;70(4):1299–311. doi: 10.1093/jxb/ery427 (PMC6382329; doi:10.1093/jxb/ery427)
Supplement: Supplementary Figures S1-S3, S5 and_Tables S1-S3 [file ery427_suppl_supplementary-figures-s1-s3_s5_tables-s1-s3.docx]

Supplementary Figures S1-S3 and S5+Tables S1-S3

Title: Multiple strategies for heat adaptation to prevent chalkiness in the rice endosperm

**Authors**

Hiroshi Wada^1,†,^*, Yuto Hatakeyama^1,†^, Yayoi Onda^2^, Hiroshi Nonami^2^, Taiken Nakashima^3^, Rosa Erra-Balsells^4^, Satoshi Morita^1^, Kenzo Hiraoka^5^, Fukuyo Tanaka^6^, and Hiroshi Nakano^1^

**Affiliations**

^1^Kyushu Okinawa Agricultural Research Center, National Agriculture and Food Research Organization, Chikugo, Fukuoka, Japan

^2^Graduate School of Agriculture, Ehime University, Matsuyama, Ehime, Japan

^3^Faculty of Agriculture, Hokkaido University, Sapporo, Hokkaido, Japan

^4^Department of Organic Chemistry, University of Buenos Aires, Buenos Aires, Argentina

^5^Clean Energy Research Center, The University of Yamanashi, Kofu, Yamanashi, Japan

^6^Central Region Agricultural Research Center, National Agriculture and Food Research Organization, Tsukuba, Ibaraki, Japan

^†^These authors contributed equally to this manuscript.

*Corresponding author: Hiroshi Wada (ORCID ID 0000-0003-0510-5744; Email: [hwada@affrc.go.jp](mailto:hwada@affrc.go.jp))

**
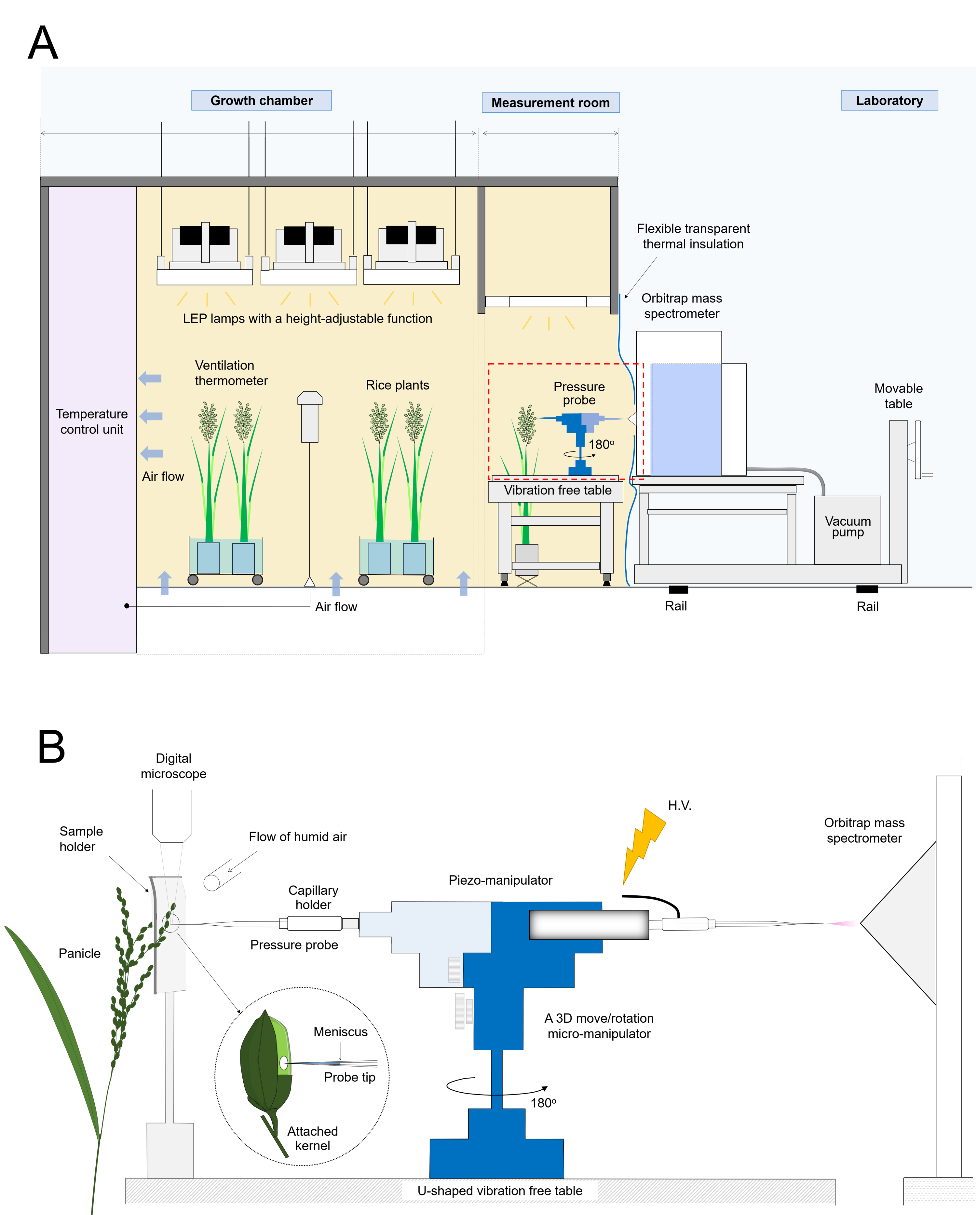
Fig. S1**

**Fig. S1** Diagram of the on-site cell metabolomics system. (A) The entire diagram of the system placed in the laboratory and (B) expanded figure of picoPPESI-MS shown in red rectangle in A. The system is composed of picolitre pressure-probe-electrospray-ionization mass spectrometry (picoPPESI-MS) and environmental control. The developing plants and the analytical environment both can be kept at the same environmental conditions using this system, as shown in light orange, isolated from the laboratory temperature by putting a flexible transparent thermal insulation (indicated in blue line) at the border. B, expanded diagram of the picoPPESI-MS system shown in red rectangle in A. This method allows to perform cell-specific metabolome analysis in intact plants being treated at a set temperature. In the dorsal outer-endosperm, a part of pericarp tissue was removed (see Methods). By utilizing a cell pressure probe, the probe tip was introduced into the target outer-endosperm cells (see Fig. 3A), where the formation of highest frequency of chalkiness is expected, and the cell sap discharged was collected. The Piezo-manipulator was immediately rotated 180° and then the metabolites in the cell sap were instantly analyzed in Orbitrap mass spectrometer by applying a high-voltage.

**Table S1**

| Table S1. Dimension of representative kernel types in each treatment. The kernel length, width, thickness, transversal area, and volume of the perfect kernels in 26°C and N+34°C treatments and white-back kernels in 34°C treatment are shown. | | | | | | |
| --- | --- | --- | --- | --- | --- | --- |
| Treatments and kernel type | Length | Width | Thickness |  | Transversal area | Volume |
|  | mm | mm | mm |  | 10^-6^ m^2^ | μL |
| Perfect kernels in 26°C | 5.21 | 3.08a | 2.15 |  | 5.09 | 9.54 |
| Chalky kernels in 34°C | 5.16 | 3.01b | 2.13 |  | 5.09 | 9.44 |
| Perfect kernels in N+34°C | 5.15 | 3.00b | 2.10 |  | 5.03 | 9.34 |
| Values in each parameter are means measured from 18-28 samples from at least 3 plants in each treatment. Means within a column followed by different letters indicate a significant difference (Tukey–Kramer test, *p* < 0.05). | | | | | | |

| Table S2. Effects of temperature and N application on the number and size of dorsal outer-endosperm cells. The number of cells in endosperm cross-sections, the cell area, and calculated volume of dorsal outer endosperm cells in the matured kernels in 26°C, 34°C, and N+34°C treatments. | | | | | |
| --- | --- | --- | --- | --- | --- |
| Treatments |  | Number of cells in  endosperm cross-sections | Outer-endosperm cells | | |
|  |  |  | Average cell area |  | Average cell volume |
|  |  |  | µm^2^ |  | pL |
| 26°C |  | 1151 | 7596a |  | 114.6a |
| 34°C |  | 1156 | 4418b |  | 66.6b |
| N+34°C |  | 1305 | 4085b |  | 61.6b |
| Values in the number and size of outer-endosperm cells are means measured from 4 and 21-28 cells in each treatment, respectively. Means within a column followed by different letters indicate a significant difference (Tukey–Kramer test, *p* < 0.05). | | | | | |

**Table S2**


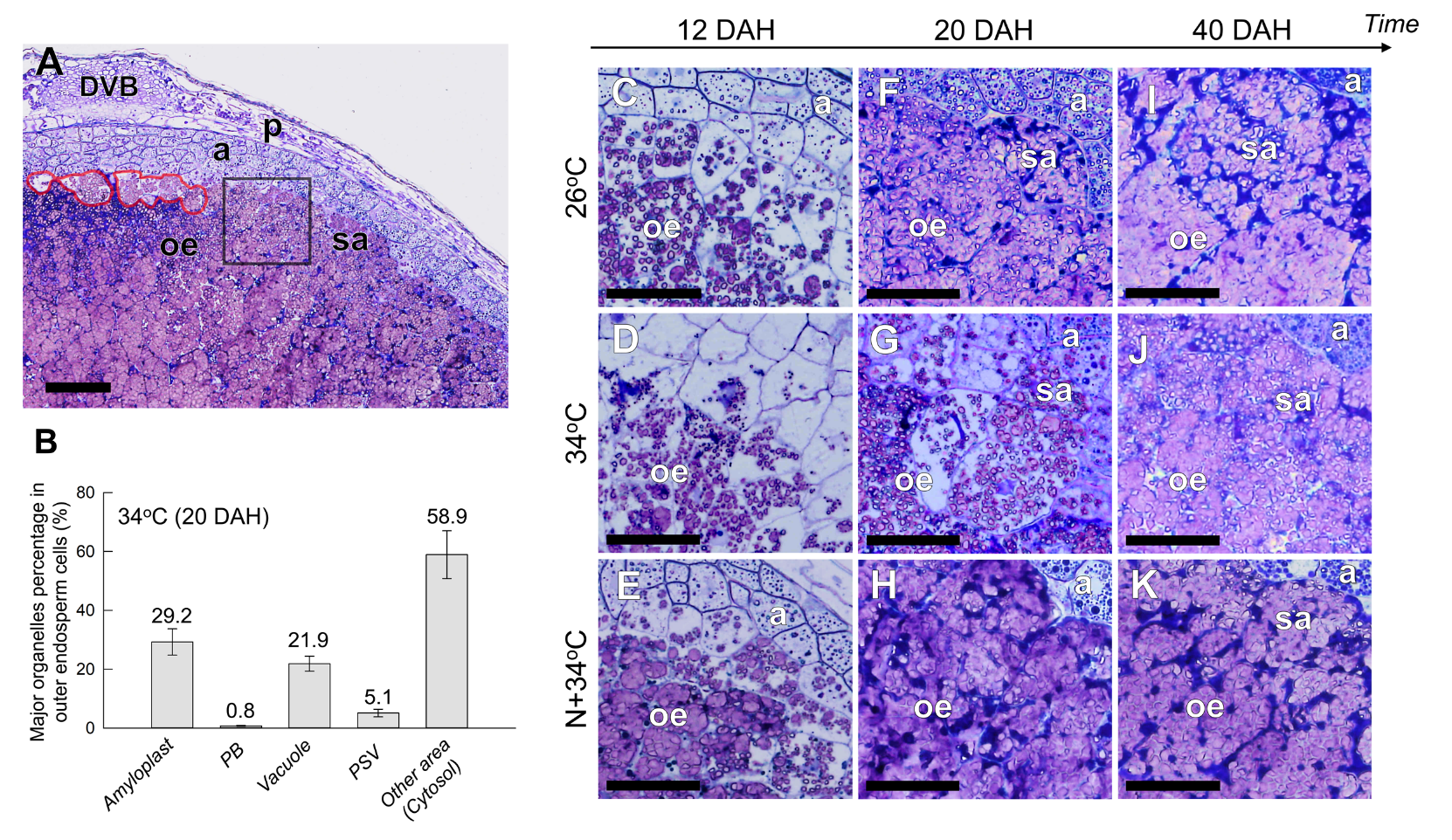
**Fig. S2**

**Fig. S2** Morphological changes in the dorsal outer-endosperm during kernel development. The transverse sections of 34°C-treated kernels collected at 20 DAH (A). Percentage of each organelle per cell in a part of putative chalky zone (traced in red in A) at 20 DAH was shown in B (see Discussion). The data in B indicate the means±SEs of 19 individual cells collected from 3 kernels from 3 individual plants. The black square in A corresponds to the area used for light microscopic observations shown in C-K. The transverse sections collected at 12 DAH (C-E), 20 DAH (F-H), and 40 DAH (I-K) in 26°C (C, F, I), 34°C (D, G, J), and N+34°C (E, H, K) treatments. DVB, dorsal vascular bundle; a, aleurone layer; oe, outer-endosperm; sa, subaleurone cell layer; p, pericarp. Bars in A and B-G indicate 100 and 50 μm, respectively.

**Fig. S3**


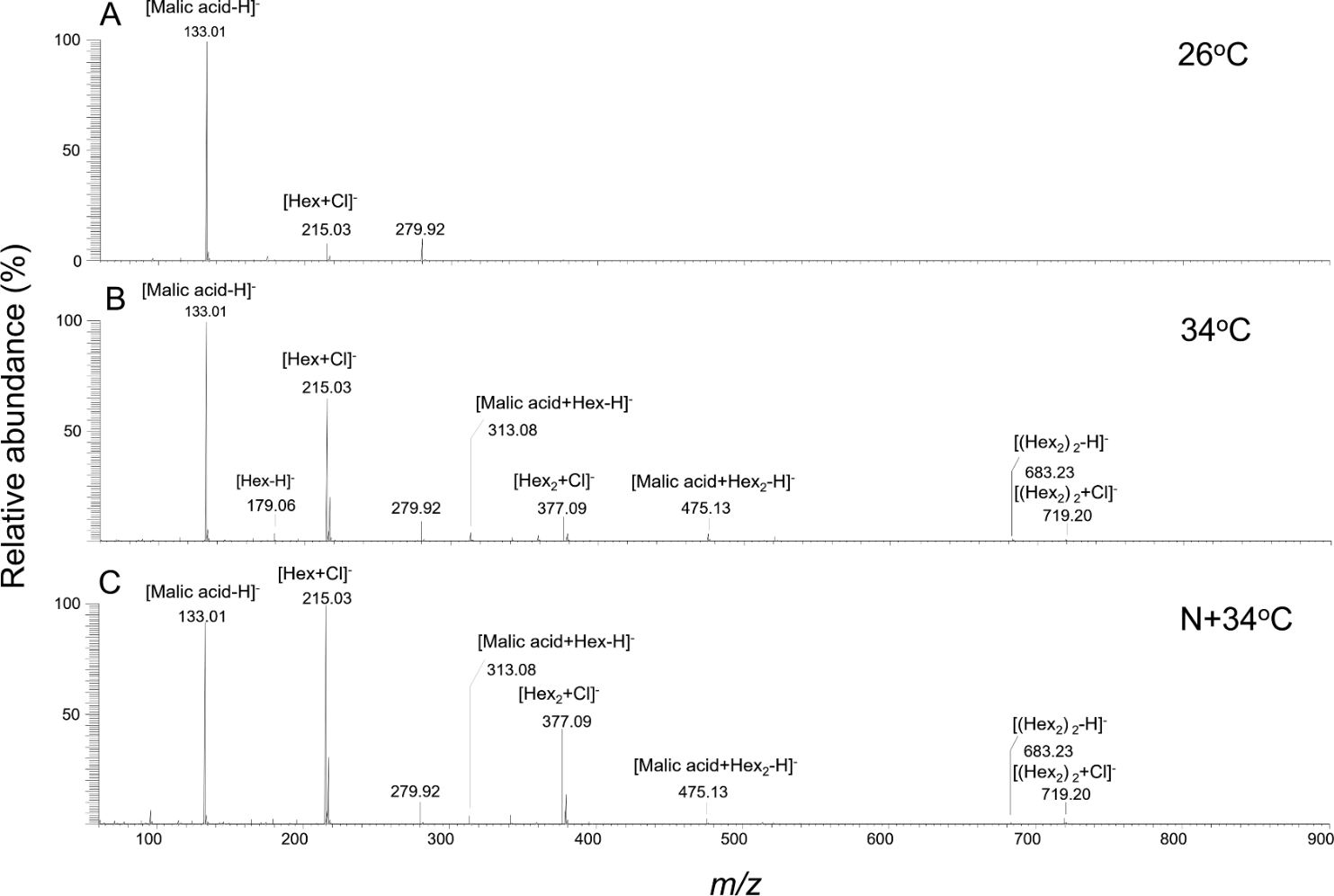


**Fig. S3** Mass spectrum of cell metabolites in the pericarps. PicoPPESI-MS negative ion mode mass spectra obtained from the pericarp cells in 26°C (A), 34°C (B), and N+34°C (C) at the same early ripening stage. Data are representative of similar experiments with 4-5 kernels in each treatment.

**Table S3**

| Table S3. List of metabolites detected in dorsal outer-endosperm cells using picoPPESI-MS in negative ion mode. | | | | | | | | | | | | | |
| --- | --- | --- | --- | --- | --- | --- | --- | --- | --- | --- | --- | --- | --- |
|  | Metabolites | Ion molecule formula | Ion type detected  [M=molecule] | Theoretical*  *m/z* | Relative abundance (%)† | | | *p* value |  | Frequency of detection‡ | | | MS/MS  shown in |
|  |  |  |  |  | 26°C | 34°C | N+34°C |  |  | 26°C | 34°C | N+34°C |  |
| Amino acids | Glycine | C2H5NO2 | [M−H]^-^ | 74.0247 | 0.15 | 0.35 | 0.12 | 0.415 |  | ++ | ++++ | +++ |  |
|  | Alanine | C3H7NO2 | [M−H]^-^ | 88.0404 | 2.41 | 3.47 | 0.78 | 0.268 |  | ++++ | ++++ | ++++ |  |
|  | GABA | C4H9NO2 | [M−H]^-^ | 102.0561 | 1.30 | 2.10 | 0.27 | 0.289 |  | ++++ | ++++ | ++++ | Figure S4-1 |
|  | Serine | C3H7NO3 | [M−H]^-^ | 104.0353 | 0.89 | 2.16 | 0.78 | 0.288 |  | ++++ | ++++ | ++++ |  |
|  | Proline | C5H9NO2 | [M−H]^-^ | 114.0561 | 0.05 | 0.03 | 0.07 | 0.798 |  | + | +++ | ++ | Figure S4-2 |
|  | Valine | C5H11NO2 | [M−H]^-^ | 116.0717 | 0.33 | 0.55 | 0.04 | 0.211 |  | +++ | ++ | + |  |
|  | Threonine | C4H9NO3 | [M−H]^-^ | 118.0510 | 0.49 | 0.77 | 0.21 | 0.260 |  | ++++ | ++++ | ++++ |  |
|  | Cysteine | C3H7NO2S | [M−H]^-^ | 120.0125 | ND | 0.22a | 0.03b | 0.009 |  | - | +++ | + |  |
|  | Leucine, Isoleucine | C6H13NO2 | [M−H]^-^ | 130.0874 | 0.48 | 0.17 | 0.03 | 0.314 |  | +++ | +++ | ++ |  |
|  | Asparagine | C4H8N2O3 | [M−H]^-^ | 131.0462 | 0.30 | 0.56 | 0.19 | 0.526 |  | ++++ | ++++ | ++ | Figure S4-3 |
|  | Aspartic acid | C4H7NO4 | [M−H]^-^ | 132.0302 | 16.36 | 28.88 | 15.44 | 0.544 |  | ++++ | ++++ | ++++ | Figure S4-4 |
|  | Glutamine | C5H10N2O3 | [M−H]^-^ | 145.0619 | 25.66 | 37.25 | 0.87 | 0.567 |  | ++++ | ++++ | ++++ | Figure S4-5 |
|  | Lysine | C6H14N2O2 | [M−H]^-^ | 145.0983 | 0.03 | 0.03 | ND | 0.536 |  | + | ++ | - |  |
|  | Glutamic acid | C5H9NO4 | [M−H]^-^ | 146.0459 | 37.90 | 58.81 | 29.05 | 0.584 |  | ++++ | ++++ | ++++ | Figure S4-6 |
|  | Methionine | C5H11NO2S | [M−H]^-^ | 148.0438 | 0.05 | 0.01 | ND | 0.273 |  | + | ++ | - | Figure S4-7 |
|  | Histidine | C6H9N3O2 | [M−H]^-^ | 154.0622 | 0.19 | 0.16 | 0.30 | 0.775 |  | +++ | ++++ | ++++ |  |
|  | Phenylalanine | C9H11NO2 | [M−H]^-^ | 164.0717 | 0.65 | 0.51 | 1.69 | 0.610 |  | ++++ | ++++ | ++++ | Figure S4-8 |
|  | Arginine | C6H14N4O2 | [M−H]^-^ | 173.1044 | 0.02 | 0.00 | ND | 0.668 |  | + | + | - |  |
|  | Tyrosine | C9H11NO3 | [M−H]^-^ | 180.0666 | 0.30 | 0.20 | 0.16 | 0.748 |  | ++++ | ++++ | ++ |  |
|  | Tryptophan | C11H12N2O2 | [M−H]^-^ | 203.0826 | 0.03 | 0.02 | ND | 0.749 |  | + | ++ | - |  |
|  | Serine | C3H7NO3 | [M+Cl]^-^ | 140.0120 | 0.34 | 0.12 | 0.35 | 0.765 |  | +++ | +++ | ++ |  |
|  | Threonine | C4H9NO3 | [M+Cl]^-^ | 154.0276 | 0.87 | 0.02 | 1.73 | 0.513 |  | +++ | ++ | ++ |  |
|  | Aspartic acid | C4H7NO4 | [M+Cl]^-^ | 168.0069 | 0.05 | ND | 0.05 | 0.516 |  | + | - | ++ |  |
|  | Glutamine | C5H10N2O3 | [M+Cl]^-^ | 181.0385 | 0.03 | ND | 0.01 | 0.161 |  | + | - | + |  |
|  | Glutamic acid | C5H9NO4 | [M+Cl]^-^ | 182.0226 | 0.07 | ND | ND | 0.422 |  | + | - | - |  |
|  | Tryptophan | C11H12N2O2 | [M+Cl]^-^ | 239.0593 | 2.56 | ND | 31.66 | 0.398 |  | ++++ | - | ++++ |  |
|  |  |  |  |  |  |  |  |  |  |  |  |  |  |
| Organic acid | Malic acid | C4H6O5 | [M−H]^-^ | 133.0142 | 100.00 | 84.11 | 100.00 | 0.204 |  | ++++ | ++++ | ++++ | Figure S4-9 |
|  | Monodehydroascorbic acid | C6H7O6 | [M−H]^-^ | 174.0170 | 0.33 | 2.25 | 1.48 | 0.294 |  | ++++ | ++++ | ++++ | Figure S4-10 |
|  | Ascorbic acid | C6H8O6 | [M−H]^-^ | 175.0247 | 20.39 | 59.64 | 20.95 | 0.182 |  | ++++ | ++++ | ++++ | Figure S4-11 |
|  |  |  |  |  |  |  |  |  |  |  |  |  |  |
| Carbohydrate | Hex | C6H12O6 | [M−H]^-^ | 179.0561 | 2.51 | 1.31 | 1.14 | 0.440 |  | ++++ | ++++ | ++++ | Figure S4-12 |
|  | HexP | C6H13O9P | [M−H]^-^ | 259.0224 | 1.73 | 3.34 | 0.42 | 0.211 |  | ++++ | ++++ | ++++ | Figure S4-13 |
|  | Hex_2_ | C12H22O11 | [M−H]^-^ | 341.1089 | 15.27 | 12.16 | 2.21 | 0.228 |  | ++++ | ++++ | ++++ | Figure S4-14 |
|  | (Hex_2_)_2_ | C24H44O22 | [M−H]^-^ | 683.2251 | 4.43 | 4.16 | 1.29 | 0.526 |  | ++++ | ++++ | ++++ |  |
|  | Hex | C6H12O6 | [M+Cl]^-^ | 215.0328 | 19.16 | 1.93 | 2.70 | 0.385 |  | ++++ | ++++ | ++++ | Figure S4-15 |
|  | Hex_2_ | C12H22O11 | [M+Cl]^-^ | 377.0856 | 10.01 | 15.63 | 5.07 | 0.534 |  | ++++ | ++++ | ++ | Figure S4-16 |
|  | (Hex_2_)_2_ | C24H44O22 | [M+Cl]^-^ | 719.2018 | 1.52 | 1.64 | 0.36 | 0.583 |  | +++ | ++++ | ++ |  |
|  |  |  |  |  |  |  |  |  |  |  |  |  |  |
| Peptide | Glutathione | C10H17N3O6S | [M−H]^-^ | 306.0766 | 0.12b | 2.4a | 0.62ab | 0.027 |  | ++ | ++++ | ++++ | Figure S4-17 |

| (*Continued from previous page*.) | | | |  |  |  |  | |  | |  |  |  |  |  |  |
| --- | --- | --- | --- | --- | --- | --- | --- | --- | --- | --- | --- | --- | --- | --- | --- | --- |
|  | | Cluster ions | Ion cluster molecular formula | Ion type detected  [M=cluster] | Theoretical*  *m/z* | Relative abundance (%)† | | | | | *p* value |  | Frequency of detection‡ | | | MS/MS  shown in |
|  |  |  |  |  |  | 26°C | | 34°C | | N+34°C |  |  | 26°C | 34°C | N+34°C |  |
| Cluster ions | | Glycine+Hex | C8H17NO8 | [M−H]^-^ | 254.0881 | 0.08 | | 0.01 | | 0.01 | 0.194 |  | ++ | ++ | + |  |
|  | | Alanine+Hex | C9H20NO8 | [M−H]^-^ | 268.1038 | 0.81 | | 0.19 | | 0.21 | 0.303 |  | +++ | ++++ | ++ |  |
|  | | GABA+Hex | C10H21NO8 | [M−H]^-^ | 282.1195 | 0.07 | | ND | | ND | 0.259 |  | ++ | - | - |  |
|  | | Serine+Hex | C9H19NO9 | [M−H]^-^ | 284.0987 | 0.65 | | 0.09 | | 0.20 | 0.336 |  | ++ | +++ | ++ |  |
|  | | Proline+Hex | C11H21NO8 | [M−H]^-^ | 294.1194 | 0.05 | | ND | | ND | 0.409 |  | + | - | - |  |
|  | | Valine+Hex | C11H23NO8 | [M−H]^-^ | 296.1351 | 0.11 | | ND | | ND | 0.103 |  | ++ | - | - |  |
|  | | Threonine+Hex | C10H21NO9 | [M−H]^-^ | 298.1143 | 0.28 | | 0.01 | | 0.07 | 0.145 |  | ++ | ++ | ++ |  |
|  | | Cysteine+Hex | C9H19NO8S | [M−H]^-^ | 300.0759 | ND | | 0.28 | | ND | 0.435 |  | - | + | - |  |
|  | | Leucine, Isoleucine+Hex | C12H23NO8 | [M−H]^-^ | 310.1507 | 0.13 | | 0.00 | | ND | 0.198 |  | ++ | + | - |  |
|  | | Asparagine+Hex | C10H20N2O9 | [M−H]^-^ | 311.1096 | 0.07 | | 0.00 | | 0.03 | 0.384 |  | ++ | - | + |  |
|  | | Aspartic acid+Hex | C10H19NO10 | [M−H]^-^ | 312.0936 | 0.61 | | 0.10 | | 0.13 | 0.204 |  | ++ | +++ | ++ |  |
|  | | Glutamine+Hex | C5H11N2O3 | [M−H]^-^ | 325.1252 | 0.60 | | ND | | 0.13 | 0.246 |  | ++ | - | ++ |  |
|  | | Glutamic acid+Hex | C11H21NO10 | [M−H]^-^ | 326.1093 | 2.32 | | 0.48 | | 0.23 | 0.160 |  | +++ | ++++ | ++ |  |
|  | | Methionine+Hex | C11H23NO8S | [M−H]^-^ | 328.1072 | ND | | 0.02 | | 0.01 | 0.693 |  | - | ++ | - |  |
|  | | Phenylalanine+Hex | C15H23NO8 | [M−H]^-^ | 344.1351 | 0.06 | | 0.00 | | 0.02 | 0.405 |  | ++ | - | - |  |
|  | | Tyrosine+Hex | C15H23NO9 | [M−H]^-^ | 360.1300 | 0.06 | | ND | | ND | 0.321 |  | + | - | - |  |
|  | | Malic acid+Hex | C10H18O11 | [M−H]^-^ | 313.0776 | 1.23 | | 0.05 | | 2.52 | 0.461 |  | ++++ | +++ | +++ |  |
|  | | Ascorbic acid+Hex | C12H20O12 | [M−H]^-^ | 355.0881 | 0.09 | | 0.02 | | 0.03 | 0.089 |  | +++ | +++ | + |  |
|  | |  |  |  |  |  | |  | |  |  |  |  |  |  |  |
|  | | Glycine+Hex_2_ | C14H27NO13 | [M−H]^-^ | 416.1410 | 0.15 | | 0.28 | | 0.07 | 0.257 |  | ++ | ++++ | ++ |  |
|  | | Alanine+Hex_2_ | C15H29NO13 | [M−H]^-^ | 430.1567 | 1.53 | | 3.11 | | 1.25 | 0.388 |  | ++ | ++++ | +++ |  |
|  | | GABA+Hex_2_ | C16H31NO13 | [M−H]^-^ | 444.1724 | 0.12 | | 0.14 | | 0.04 | 0.454 |  | ++ | +++ | ++ |  |
|  | | Serine+Hex_2_ | C15H29NO14 | [M−H]^-^ | 446.1516 | 1.12 | | 3.30 | | 1.62 | 0.482 |  | ++++ | ++++ | ++++ |  |
|  | | Proline+Hex_2_ | C17H31NO13 | [M−H]^-^ | 456.1723 | 0.11 | | 0.07 | | 0.03 | 0.543 |  | ++ | +++ | + |  |
|  | | Valine+Hex_2_ | C17H33NO13 | [M−H]^-^ | 458.1880 | 0.21 | | 0.28 | | 0.08 | 0.662 |  | ++ | +++ | ++ |  |
|  | | Threonine+Hex_2_ | C16H31NO14 | [M−H]^-^ | 460.1672 | 0.37 | | 0.95 | | 0.28 | 0.366 |  | +++ | ++++ | ++ |  |
|  | | Cysteine+Hex_2_ | C15H29NO13S | [M−H]^-^ | 462.1288 | ND | | 0.31 | | 0.09 | 0.223 |  | - | ++++ | + | Figure S4-18 |
|  | | Leucine, Isoleucine+Hex_2_ | C18H35NO13 | [M−H]^-^ | 472.2036 | 0.18 | | 0.16 | | 0.05 | 0.627 |  | ++ | +++ | + |  |
|  | | Asparagine+Hex_2_ | C16H30N2O14 | [M−H]^-^ | 473.1625 | 0.14 | | 0.42 | | 0.30 | 0.655 |  | ++ | ++++ | ++ |  |
|  | | Aspartic acid+Hex_2_ | C16H29NO15 | [M−H]^-^ | 474.1465 | 2.48 | | 7.31 | | 2.29 | 0.439 |  | ++++ | ++++ | +++ |  |
|  | | Glutamine+Hex_2_ | C5H11N2O3 | [M−H]^-^ | 487.1781 | 1.73 | | 1.94 | | 1.16 | 0.849 |  | ++ | ++++ | +++ |  |
|  | | Lysine+Hex_2_ | C18H36N2O13 | [M−H]^-^ | 487.2145 | 0.04 | | 0.06 | | 0.01 | 0.638 |  | + | ++ | + |  |
|  | | Glutamic acid+Hex_2_ | C17H31NO15 | [M−H]^-^ | 488.1622 | 8.41 | | 20.25 | | 6.76 | 0.438 |  | ++++ | ++++ | ++++ |  |
|  | | Methionine+Hex_2_ | C17H33NO13S | [M−H]^-^ | 490.1601 | ND | | ND | | 0.01 | 0.405 |  | - | - | + |  |
|  | | Histidine+Hex_2_ | C18H31N3O13 | [M−H]^-^ | 496.1785 | 0.05 | | 0.04 | | 0.03 | 0.903 |  | + | +++ | + |  |
|  | | Phenylalanine+Hex_2_ | C21H33NO13 | [M−H]^-^ | 506.1880 | 0.07 | | 0.01 | | 0.02 | 0.240 |  | ++ | + | + |  |
|  | | Arginine+Hex_2_ | C18H36N4O13 | [M−H]^-^ | 515.2207 | 0.01 | | ND | | ND | 0.405 |  | + | - | - |  |
|  | | Tyrosine+Hex_2_ | C21H33NO14 | [M−H]^-^ | 522.1829 | 0.12 | | ND | | 0.06 | 0.640 |  | ++ | - | + |  |
|  | | Glutathione+Hex_2_ | C22H39N3O17S | [M−H]^-^ | 648.1928 | ND | | 0.28 | | 0.08 | 0.431 |  | - | +++ | + |  |
|  | | Malic acid+Hex_2_ | C16H28O16 | [M−H]^-^ | 475.1305 | 4.39 | | 4.57 | | 2.05 | 0.681 |  | ++++ | ++++ | +++ |  |
|  | | Ascorbic acid+Hex_2_ | C18H30O17 | [M−H]^-^ | 517.1410 | 0.14 | | 3.55 | | 0.78 | 0.135 |  | ++ | ++++ | +++ |  |
|  | * All the theoretical values are quoted from Metlin (http://metlin.scripps.edu/index.php).  † Values were calculated as a percentage to the base peak. Different letters indicate a significant difference (Tukey HSD test, *p* < 0.05)  ‡ Signal detected (+); Signal not detected (-). Frequencies of detection (x) in 4-5 individual measurements are indicated as: ++++; 75%< x < 100%, +++; 50%< x < 75%, ++; 25%< x < 50%, +; 0%< x <25%.  ND indicates not detected. | | | | | | | | | | | | | | | |

**Fig. S5**

**
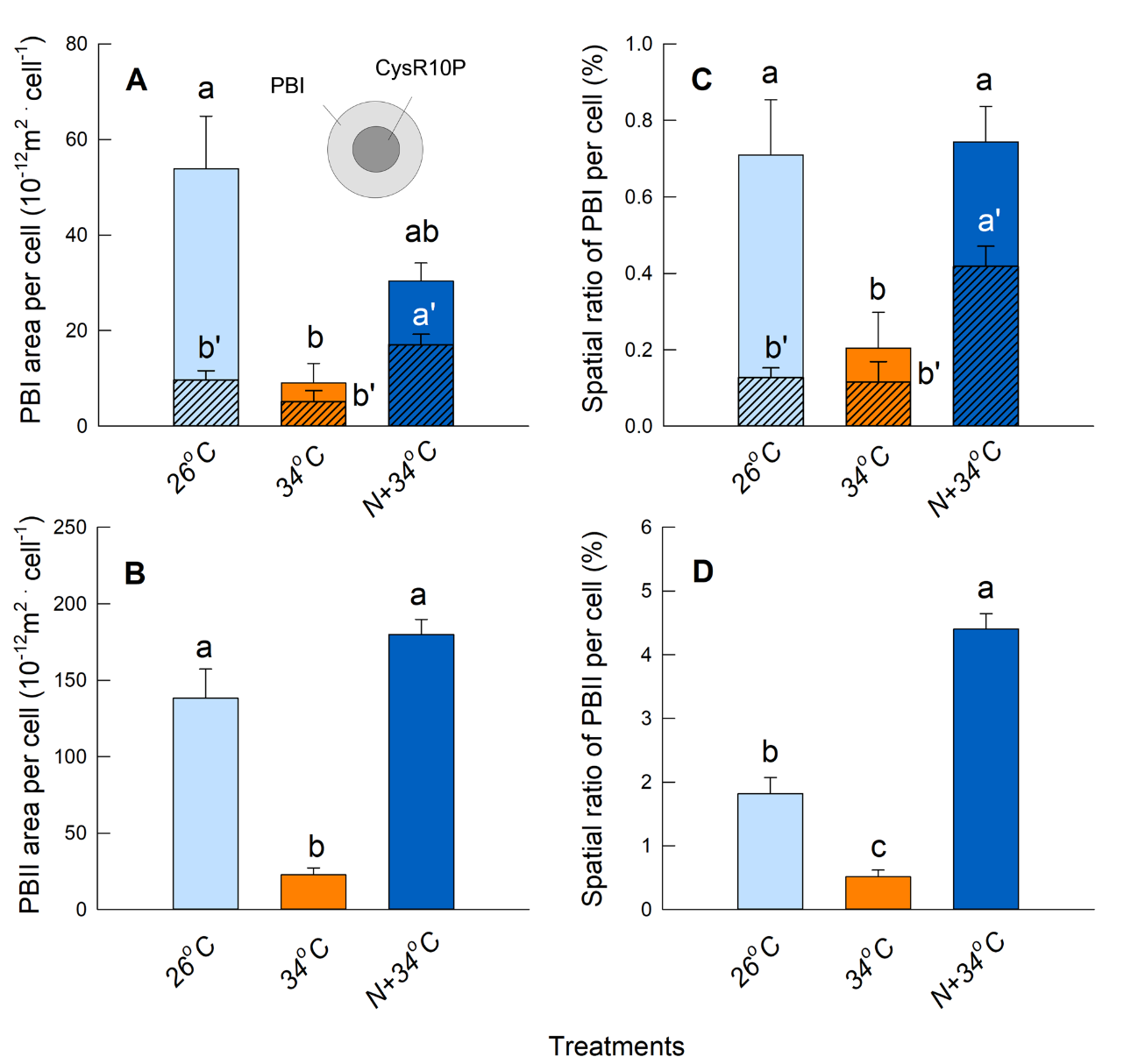
**

**Fig. S5** Areas and spatial ratios of PBIs and PBIIs in outer-endosperm cells in the kernels at 40 DAH. Calculated area of PBIs (A) and PBIIs (B) per outer-endosperm cell, and the spatial ratio of PBIs (C) and PBIIs (D) per calculated cell area. In A and B, each parameter was calculated by multiplying the number and averaged area of each PB (Fig. 4 C-F). The data indicate the mean±SEs of 9-11 cells from three kernels. In A and C, each hatched bar embedded in the bar showing the area of PBIs indicates the area of cysteine-rich 10 kDa layer (see inset in A). Different letters represent significant differences among treatments (Tukey–Kramer test, *p* < 0.05) within the area of both PBs (letter only) and area of Cys-10-kDa layer of PBI (’).
