# Supplementary material for: Multiple strategies for heat adaptation to prevent chalkiness in the rice endosperm
Source: J Exp Bot. 2018 Dec 3;70(4):1299–311. doi: 10.1093/jxb/ery427 (PMC6382329; doi:10.1093/jxb/ery427)
Supplement: Supplementary Figures S4 [file ery427_suppl_supplementary_figure_s4.pptx]

## Slide 1
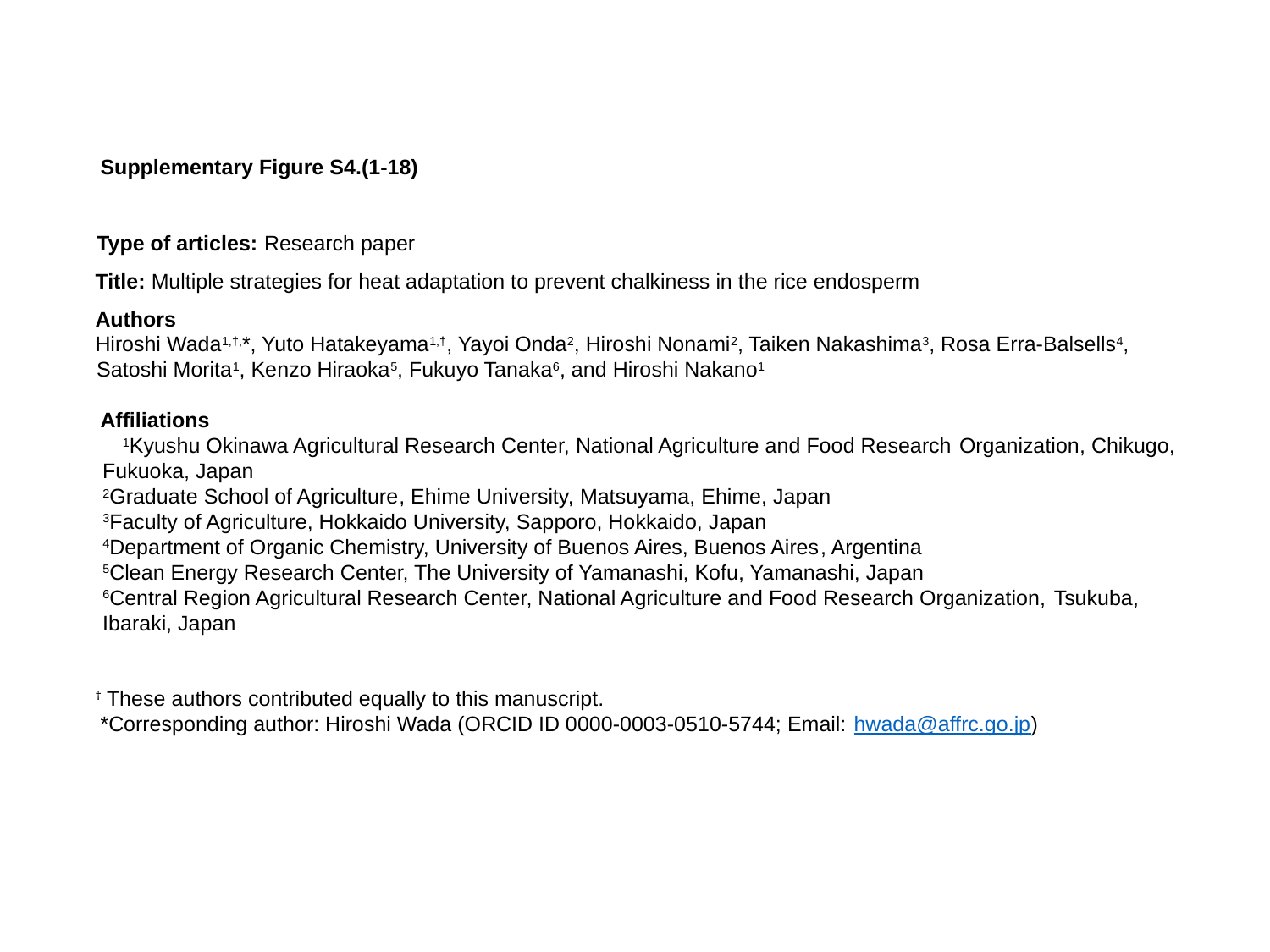

# Supplementary Figure S4.(1-18) Type of articles: Research paper
Title: Multiple strategies for heat adaptation to prevent chalkiness in the rice endosperm
Authors
Hiroshi Wada1,†,*, Yuto Hatakeyama1,†, Yayoi Onda2, Hiroshi Nonami2, Taiken Nakashima3, Rosa Erra-Balsells4,
 Satoshi Morita1, Kenzo Hiraoka5, Fukuyo Tanaka6, and Hiroshi Nakano1
　　　Affiliations
　　　　　　　1Kyushu Okinawa Agricultural Research Center, National Agriculture and Food Research Organization, Chikugo,
 Fukuoka, Japan
 2Graduate School of Agriculture, Ehime University, Matsuyama, Ehime, Japan
 3Faculty of Agriculture, Hokkaido University, Sapporo, Hokkaido, Japan
 4Department of Organic Chemistry, University of Buenos Aires, Buenos Aires, Argentina
 5Clean Energy Research Center, The University of Yamanashi, Kofu, Yamanashi, Japan
 6Central Region Agricultural Research Center, National Agriculture and Food Research Organization, Tsukuba,
 Ibaraki, Japan
　　　†These authors contributed equally to this manuscript.
　　　*Corresponding author: Hiroshi Wada (ORCID ID 0000-0003-0510-5744; Email: hwada@affrc.go.jp)

## Slide 2
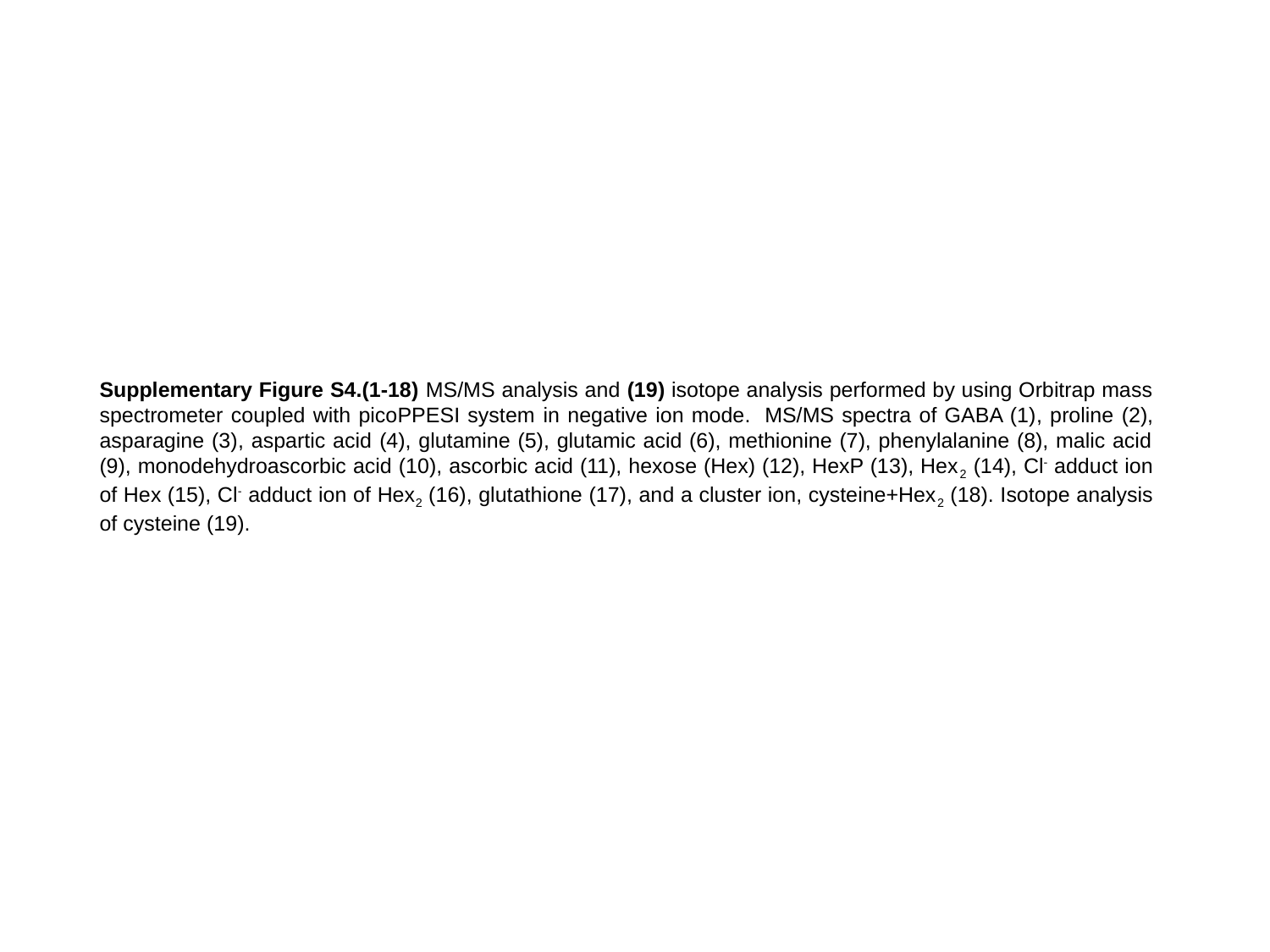

Supplementary Figure S4.(1-18) MS/MS analysis and (19) isotope analysis performed by using Orbitrap mass spectrometer coupled with picoPPESI system in negative ion mode.  MS/MS spectra of GABA (1), proline (2), asparagine (3), aspartic acid (4), glutamine (5), glutamic acid (6), methionine (7), phenylalanine (8), malic acid (9), monodehydroascorbic acid (10), ascorbic acid (11), hexose (Hex) (12), HexP (13), Hex2 (14), Cl- adduct ion of Hex (15), Cl- adduct ion of Hex2 (16), glutathione (17), and a cluster ion, cysteine+Hex2 (18). Isotope analysis of cysteine (19).

## Slide 3
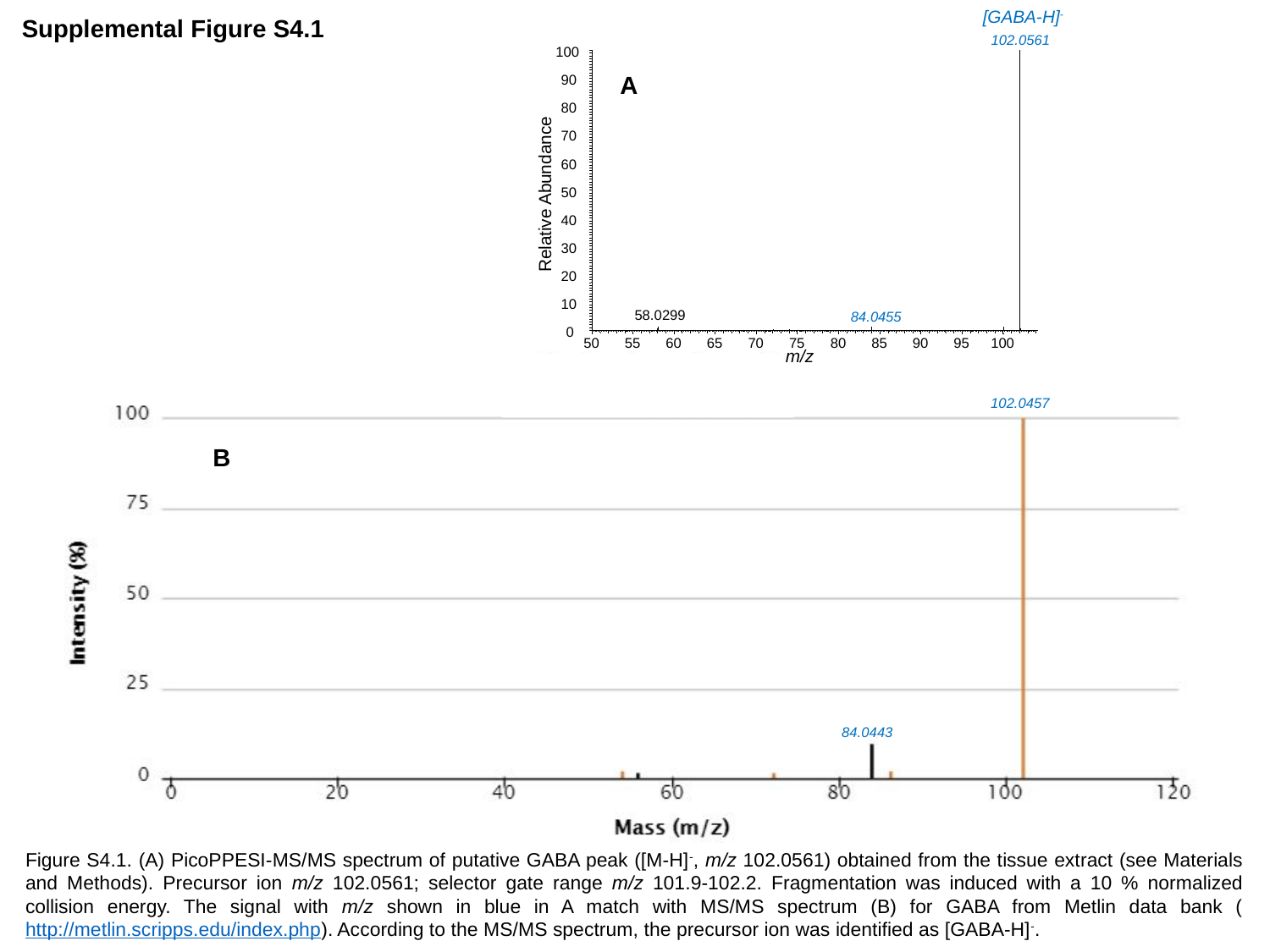

[GABA-H]-
102.0561
100
50
40
30
20
10
0
50
55
60
65
70
75
80
85
90
95
100
m/z
90
80
70
Relative Abundance
60
58.0299
84.0455
Supplemental Figure S4.1
A
102.0457
B
84.0443
Figure S4.1. (A) PicoPPESI-MS/MS spectrum of putative GABA peak ([M-H]-, m/z 102.0561) obtained from the tissue extract (see Materials and Methods). Precursor ion m/z 102.0561; selector gate range m/z 101.9-102.2. Fragmentation was induced with a 10 % normalized collision energy. The signal with m/z shown in blue in A match with MS/MS spectrum (B) for GABA from Metlin data bank (http://metlin.scripps.edu/index.php). According to the MS/MS spectrum, the precursor ion was identified as [GABA-H]-.

## Slide 4
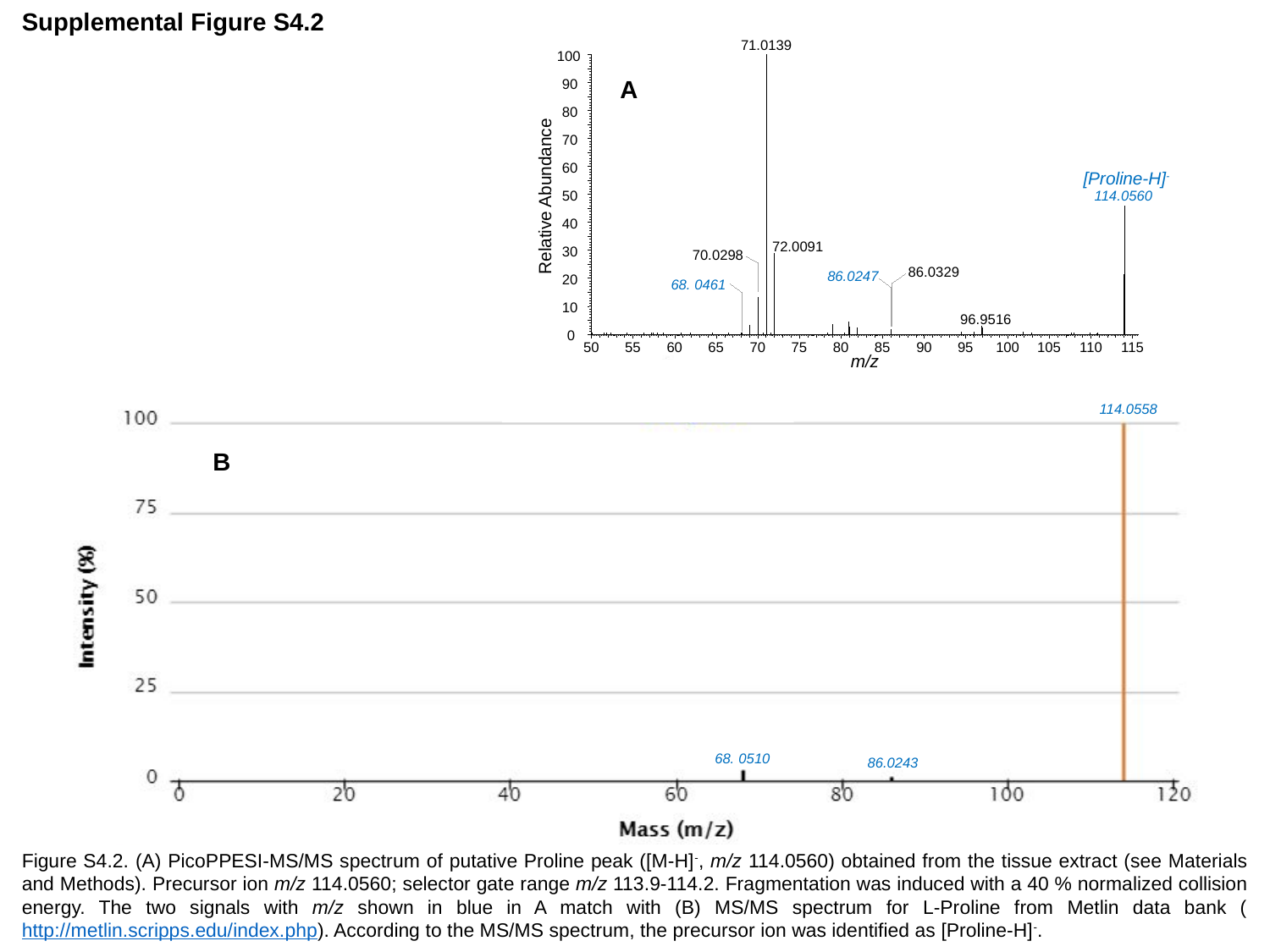

Supplemental Figure S4.2
71.0139
100
50
55
60
65
70
75
80
85
90
95
100
105
110
115
m/z
90
80
70
Relative Abundance
60
[Proline-H]-
50
114.0560
40
72.0091
30
70.0298
86.0329
86.0247
20
68. 0461
10
96.9516
0
A
114.0558
B
68. 0510
86.0243
Figure S4.2. (A) PicoPPESI-MS/MS spectrum of putative Proline peak ([M-H]-, m/z 114.0560) obtained from the tissue extract (see Materials and Methods). Precursor ion m/z 114.0560; selector gate range m/z 113.9-114.2. Fragmentation was induced with a 40 % normalized collision energy. The two signals with m/z shown in blue in A match with (B) MS/MS spectrum for L-Proline from Metlin data bank (http://metlin.scripps.edu/index.php). According to the MS/MS spectrum, the precursor ion was identified as [Proline-H]-.

## Slide 5
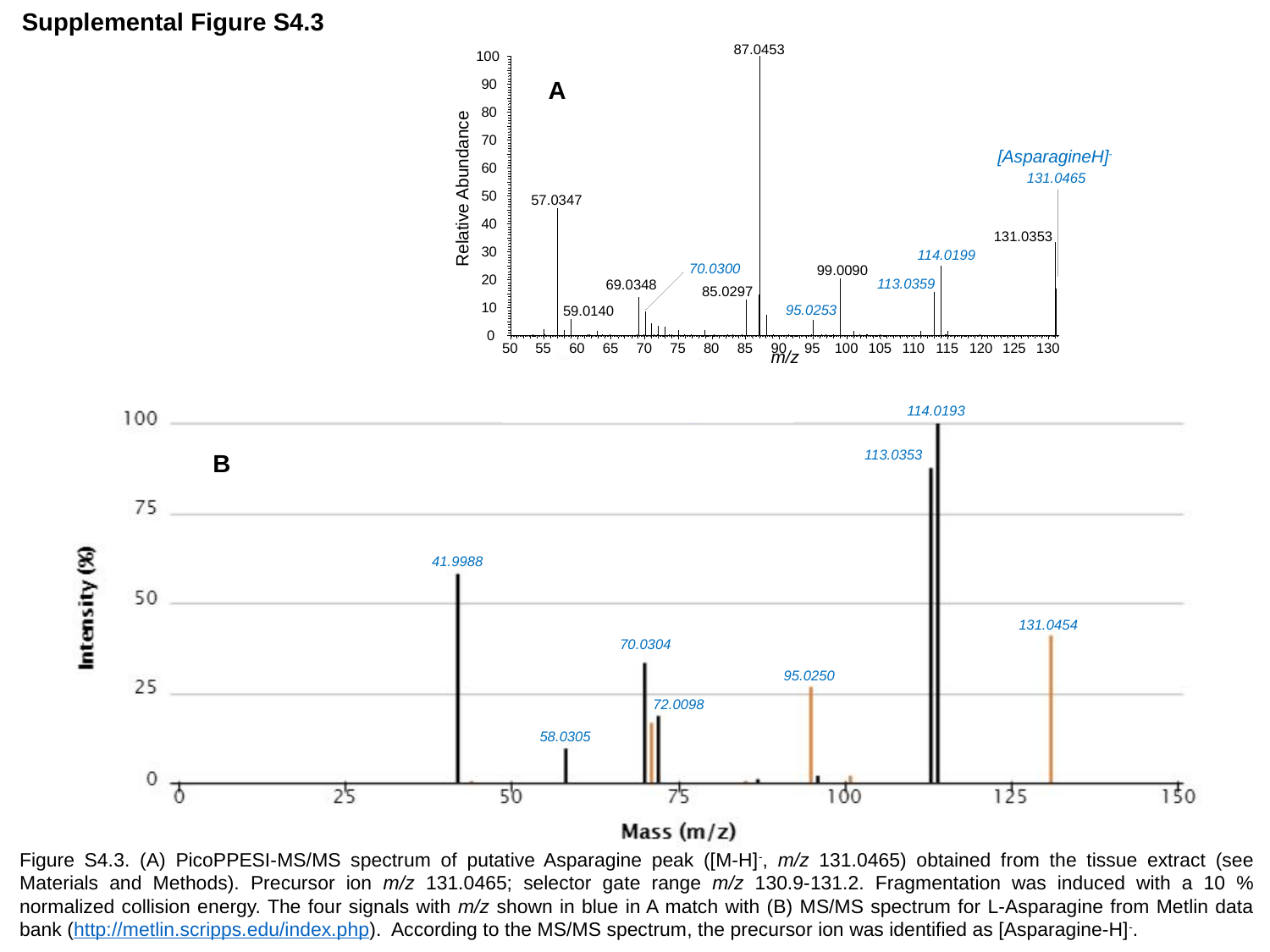

Supplemental Figure S4.3
87.0453
100
90
80
70
Relative Abundance
60
131.0465
50
57.0347
40
131.0353
30
114.0199
70.0300
99.0090
20
113.0359
69.0348
85.0297
10
95.0253
59.0140
0
50
55
60
65
70
75
80
85
90
95
100
105
110
115
120
125
130
m/z
A
114.0193
B
113.0353
41.9988
131.0454
70.0304
95.0250
72.0098
58.0305
Figure S4.3. (A) PicoPPESI-MS/MS spectrum of putative Asparagine peak ([M-H]-, m/z 131.0465) obtained from the tissue extract (see Materials and Methods). Precursor ion m/z 131.0465; selector gate range m/z 130.9-131.2. Fragmentation was induced with a 10 % normalized collision energy. The four signals with m/z shown in blue in A match with (B) MS/MS spectrum for L-Asparagine from Metlin data bank (http://metlin.scripps.edu/index.php). According to the MS/MS spectrum, the precursor ion was identified as [Asparagine-H]-.

## Slide 6
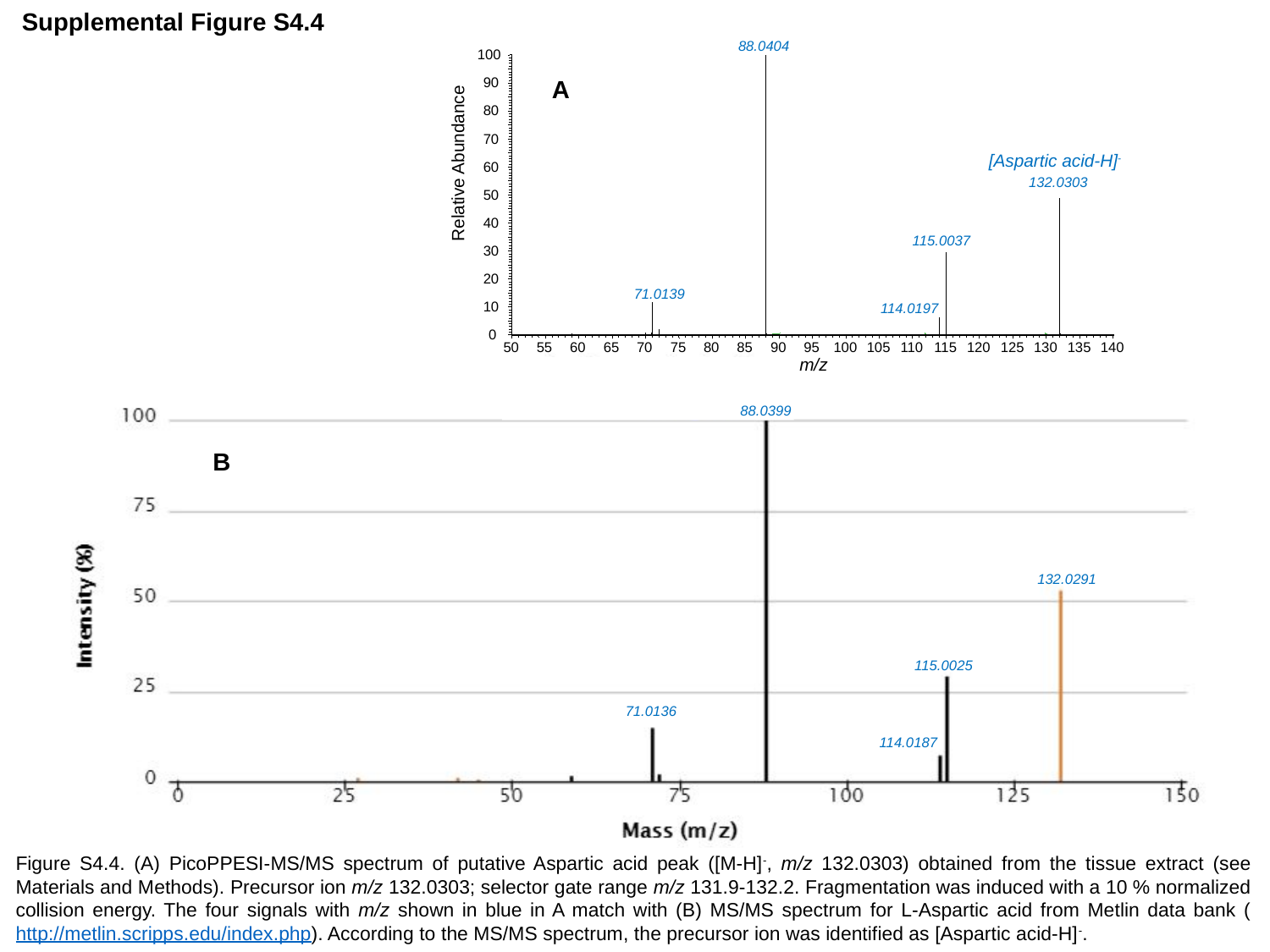

Supplemental Figure S4.4
88.0404
100
90
80
70
Relative Abundance
[Aspartic acid-H]-
60
132.0303
50
40
115.0037
30
20
71.0139
10
114.0197
0
50
55
60
65
70
75
80
85
90
95
100
105
110
115
120
125
130
135
140
m/z
A
88.0399
B
132.0291
115.0025
71.0136
114.0187
Figure S4.4. (A) PicoPPESI-MS/MS spectrum of putative Aspartic acid peak ([M-H]-, m/z 132.0303) obtained from the tissue extract (see Materials and Methods). Precursor ion m/z 132.0303; selector gate range m/z 131.9-132.2. Fragmentation was induced with a 10 % normalized collision energy. The four signals with m/z shown in blue in A match with (B) MS/MS spectrum for L-Aspartic acid from Metlin data bank (http://metlin.scripps.edu/index.php). According to the MS/MS spectrum, the precursor ion was identified as [Aspartic acid-H]-.

## Slide 7
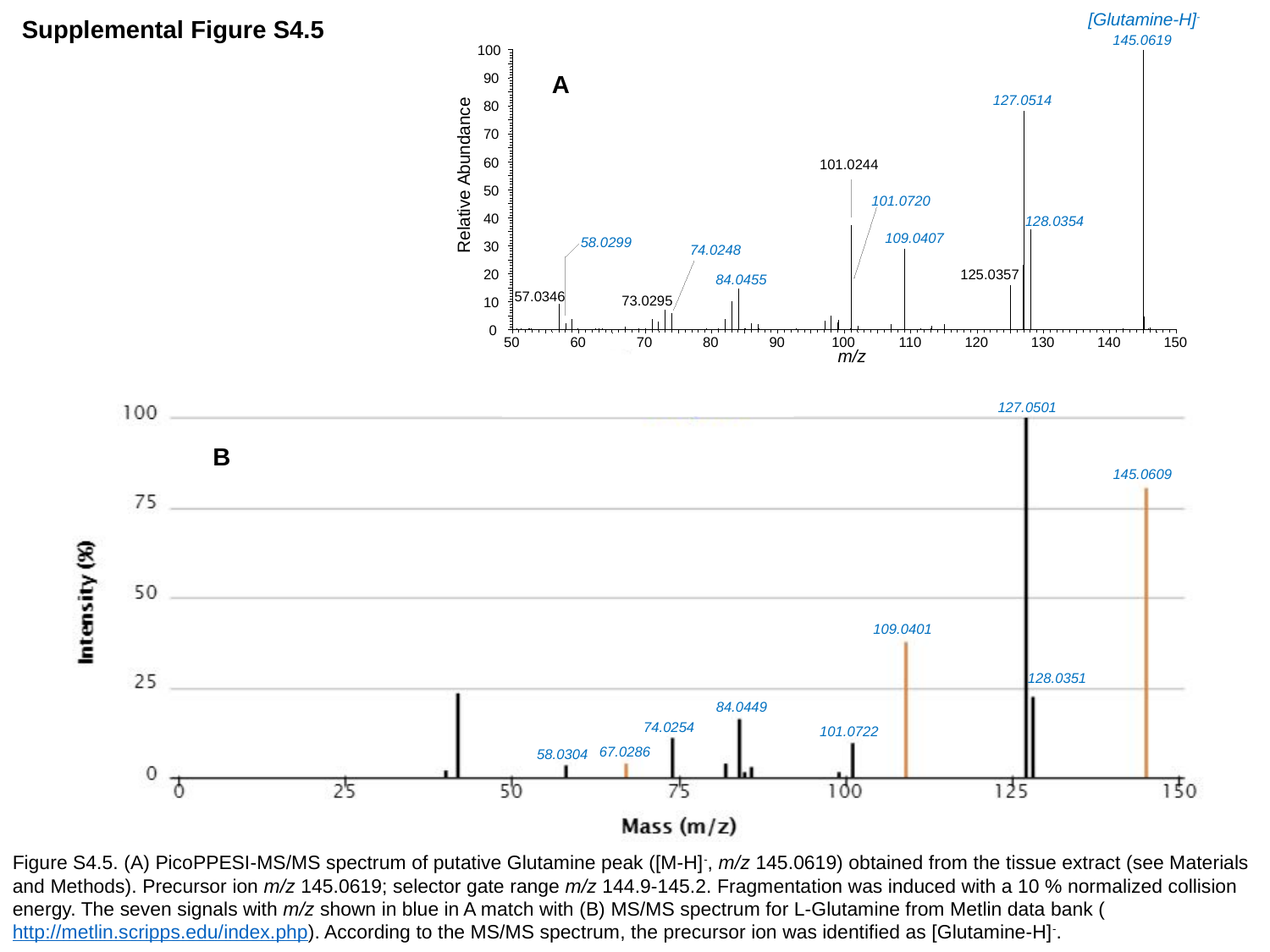

[Glutamine-H]-
145.0619
100
90
127.0514
80
70
Relative Abundance
60
101.0244
50
101.0720
40
128.0354
109.0407
58.0299
30
74.0248
20
125.0357
84.0455
57.0346
73.0295
10
0
50
60
70
80
90
100
110
120
130
140
150
m/z
Supplemental Figure S4.5
A
127.0501
B
145.0609
109.0401
128.0351
84.0449
74.0254
101.0722
67.0286
58.0304
Figure S4.5. (A) PicoPPESI-MS/MS spectrum of putative Glutamine peak ([M-H]-, m/z 145.0619) obtained from the tissue extract (see Materials and Methods). Precursor ion m/z 145.0619; selector gate range m/z 144.9-145.2. Fragmentation was induced with a 10 % normalized collision energy. The seven signals with m/z shown in blue in A match with (B) MS/MS spectrum for L-Glutamine from Metlin data bank (http://metlin.scripps.edu/index.php). According to the MS/MS spectrum, the precursor ion was identified as [Glutamine-H]-.

## Slide 8
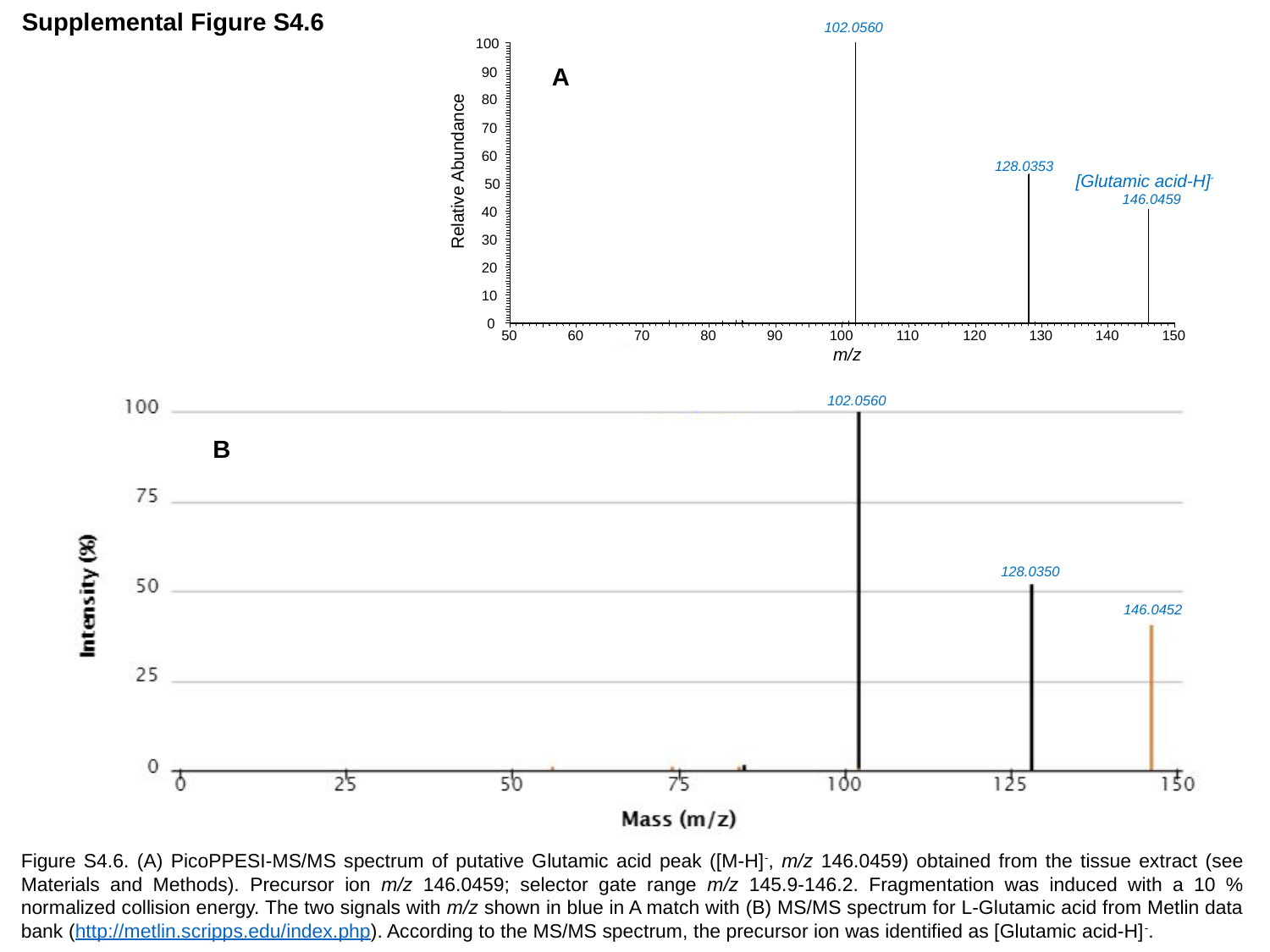

Supplemental Figure S4.6
102.0560
100
90
80
70
Relative Abundance
60
128.0353
[Glutamic acid-H]-
50
146.0459
40
30
20
10
0
50
60
70
80
90
100
110
120
130
140
150
m/z
A
102.0560
B
128.0350
146.0452
Figure S4.6. (A) PicoPPESI-MS/MS spectrum of putative Glutamic acid peak ([M-H]-, m/z 146.0459) obtained from the tissue extract (see Materials and Methods). Precursor ion m/z 146.0459; selector gate range m/z 145.9-146.2. Fragmentation was induced with a 10 % normalized collision energy. The two signals with m/z shown in blue in A match with (B) MS/MS spectrum for L-Glutamic acid from Metlin data bank (http://metlin.scripps.edu/index.php). According to the MS/MS spectrum, the precursor ion was identified as [Glutamic acid-H]-.

## Slide 9
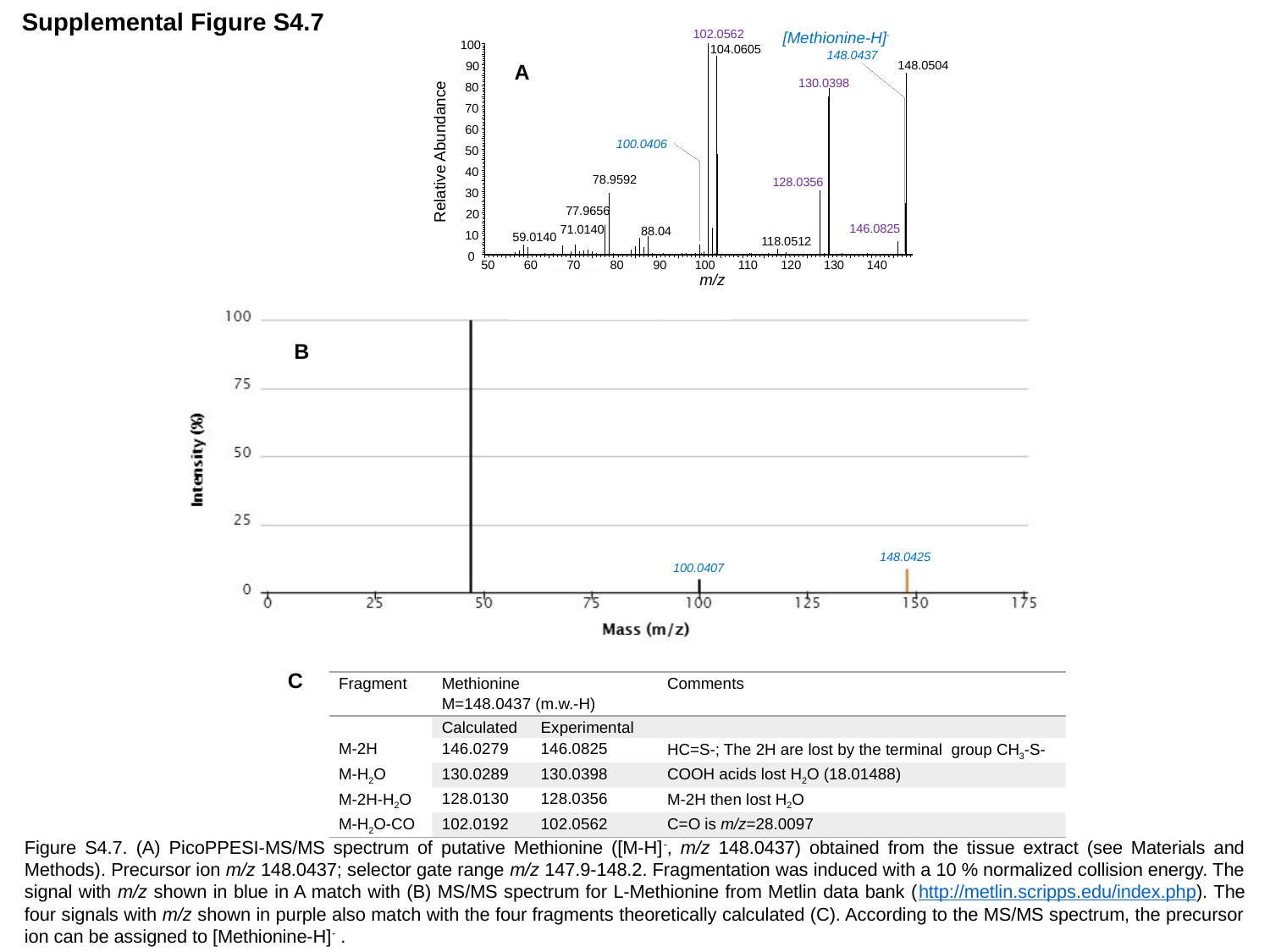

Supplemental Figure S4.7
[Methionine-H]-
102.0562
100
104.0605
148.0437
148.0504
90
130.0398
80
70
Relative Abundance
60
100.0406
50
40
78.9592
128.0356
30
77.9656
20
146.0825
71.0140
88.04
10
59.0140
118.0512
0
50
60
70
80
90
100
110
120
130
140
m/z
A
B
148.0425
100.0407
C
| Fragment | Methionine M=148.0437 (m.w.-H) | | Comments |
| --- | --- | --- | --- |
| | Calculated | Experimental | |
| M-2H | 146.0279 | 146.0825 | HC=S-; The 2H are lost by the terminal group CH3-S- |
| M-H2O | 130.0289 | 130.0398 | COOH acids lost H2O (18.01488) |
| M-2H-H2O | 128.0130 | 128.0356 | M-2H then lost H2O |
| M-H2O-CO | 102.0192 | 102.0562 | C=O is m/z=28.0097 |
Figure S4.7. (A) PicoPPESI-MS/MS spectrum of putative Methionine ([M-H]-, m/z 148.0437) obtained from the tissue extract (see Materials and Methods). Precursor ion m/z 148.0437; selector gate range m/z 147.9-148.2. Fragmentation was induced with a 10 % normalized collision energy. The signal with m/z shown in blue in A match with (B) MS/MS spectrum for L-Methionine from Metlin data bank (http://metlin.scripps.edu/index.php). The four signals with m/z shown in purple also match with the four fragments theoretically calculated (C). According to the MS/MS spectrum, the precursor ion can be assigned to [Methionine-H]- .

## Slide 10
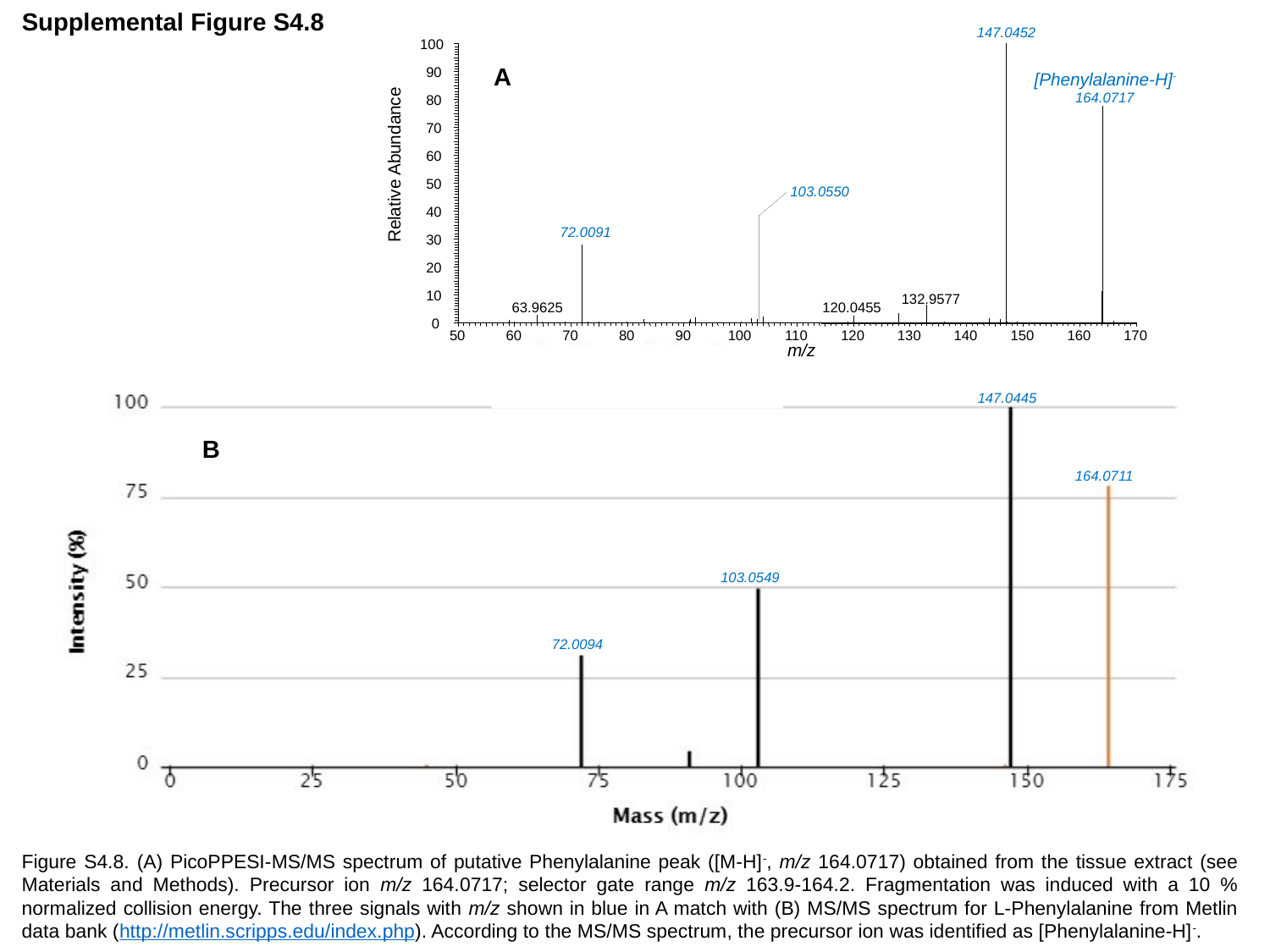

Supplemental Figure S4.8
147.0452
100
[Phenylalanine-H]-
90
164.0717
80
70
Relative Abundance
60
50
103.0550
40
72.0091
30
20
10
132.9577
120.0455
63.9625
0
50
60
70
80
90
100
110
120
130
140
150
160
170
m/z
A
147.0445
B
164.0711
103.0549
72.0094
Figure S4.8. (A) PicoPPESI-MS/MS spectrum of putative Phenylalanine peak ([M-H]-, m/z 164.0717) obtained from the tissue extract (see Materials and Methods). Precursor ion m/z 164.0717; selector gate range m/z 163.9-164.2. Fragmentation was induced with a 10 % normalized collision energy. The three signals with m/z shown in blue in A match with (B) MS/MS spectrum for L-Phenylalanine from Metlin data bank (http://metlin.scripps.edu/index.php). According to the MS/MS spectrum, the precursor ion was identified as [Phenylalanine-H]-.

## Slide 11
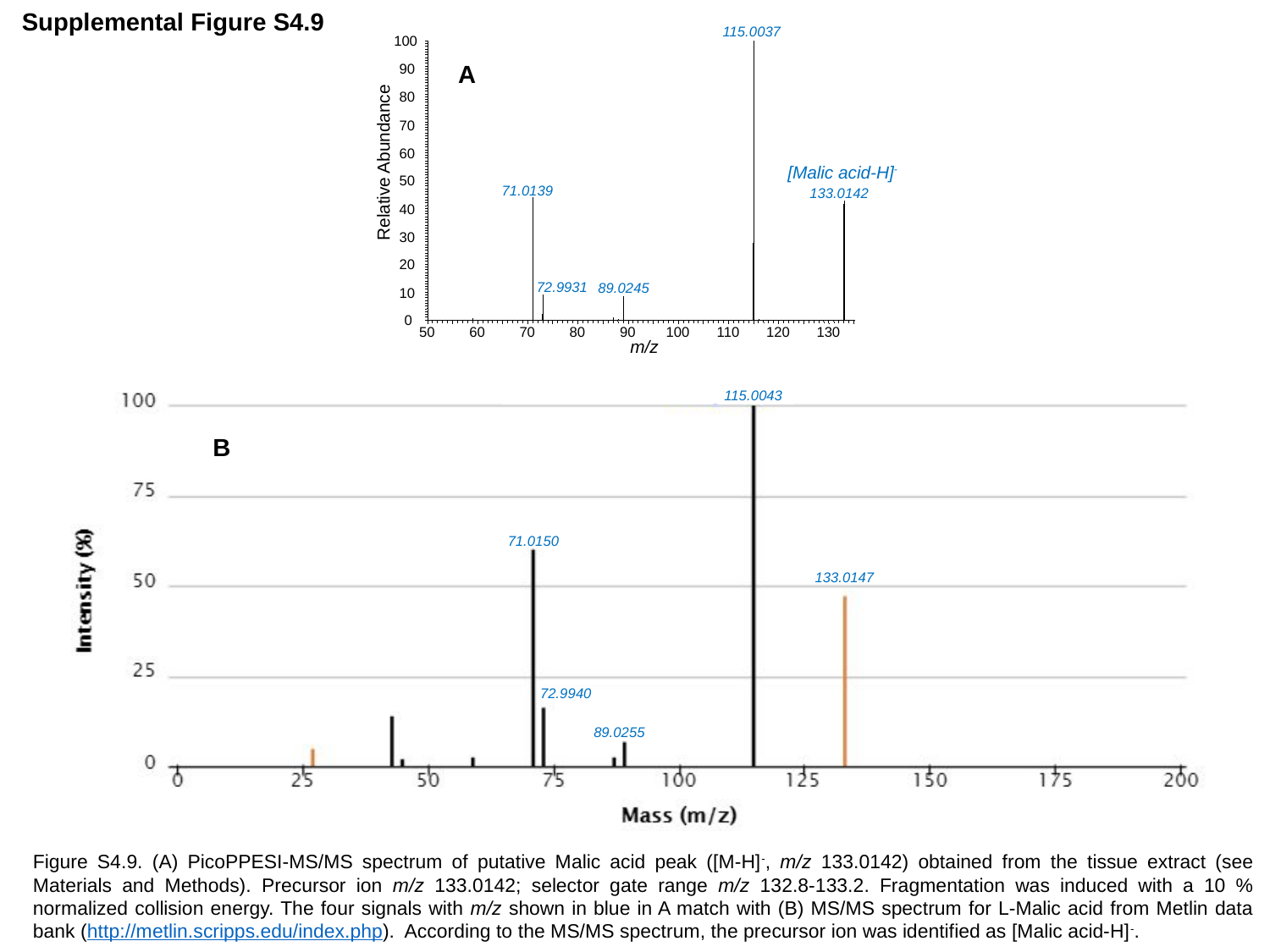

Supplemental Figure S4.9
115.0037
100
90
80
70
Relative Abundance
60
[Malic acid-H]-
50
71.0139
133.0142
40
30
20
72.9931
89.0245
10
0
50
60
70
80
90
100
110
120
130
m/z
A
115.0043
B
71.0150
133.0147
72.9940
89.0255
Figure S4.9. (A) PicoPPESI-MS/MS spectrum of putative Malic acid peak ([M-H]-, m/z 133.0142) obtained from the tissue extract (see Materials and Methods). Precursor ion m/z 133.0142; selector gate range m/z 132.8-133.2. Fragmentation was induced with a 10 % normalized collision energy. The four signals with m/z shown in blue in A match with (B) MS/MS spectrum for L-Malic acid from Metlin data bank (http://metlin.scripps.edu/index.php). According to the MS/MS spectrum, the precursor ion was identified as [Malic acid-H]-.

## Slide 12
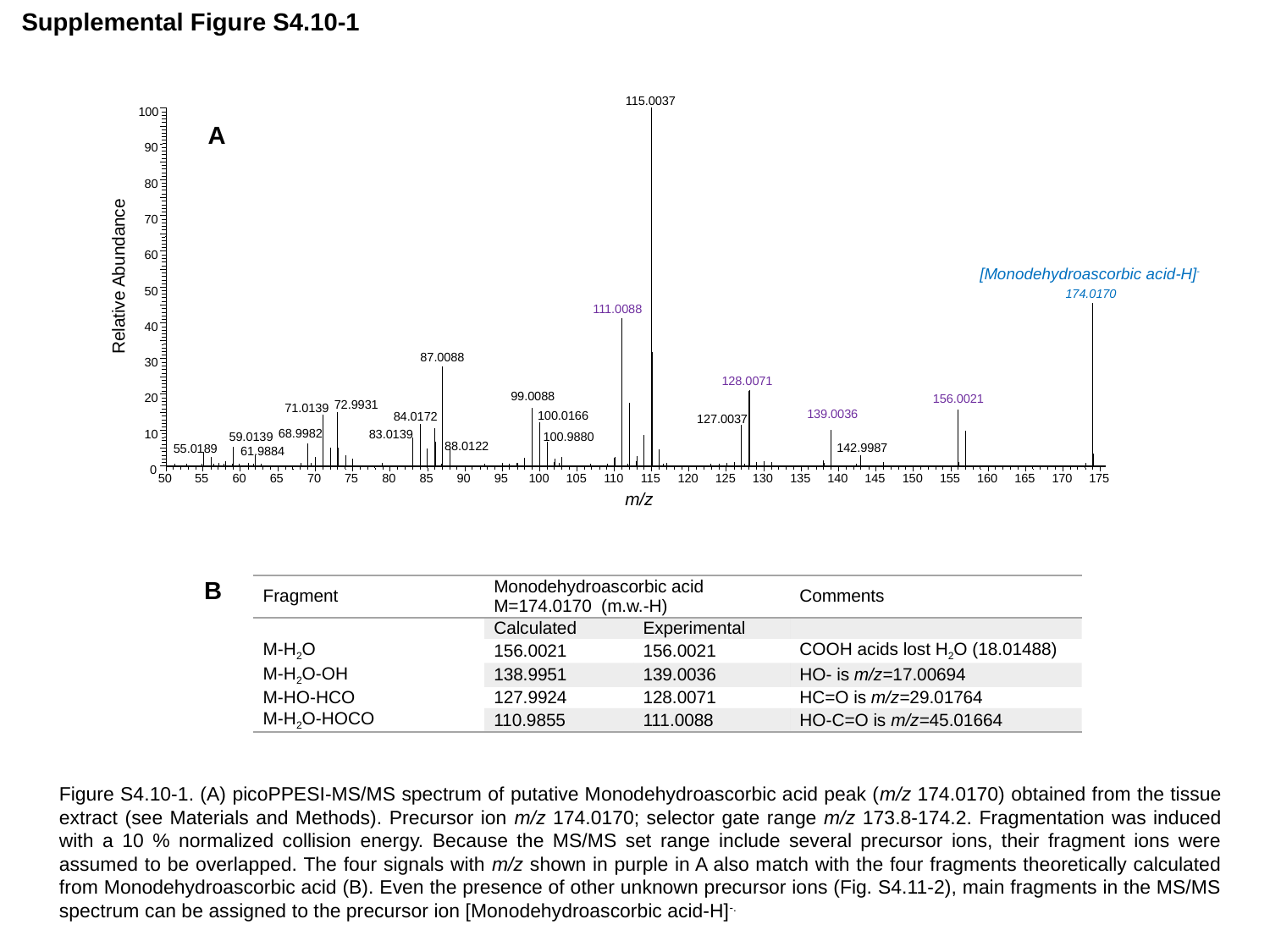

Supplemental Figure S4.10-1
115.0037
100
A
90
80
70
60
[Monodehydroascorbic acid-H]-
Relative Abundance
50
174.0170
111.0088
40
87.0088
30
128.0071
99.0088
20
156.0021
72.9931
71.0139
139.0036
100.0166
84.0172
127.0037
68.9982
10
83.0139
100.9880
59.0139
88.0122
142.9987
55.0189
61.9884
0
50
55
60
65
70
75
80
85
90
95
100
105
110
115
120
125
130
135
140
145
150
155
160
165
170
175
m/z
B
| Fragment | Monodehydroascorbic acid M=174.0170 (m.w.-H) | | Comments |
| --- | --- | --- | --- |
| | Calculated | Experimental | |
| M-H2O | 156.0021 | 156.0021 | COOH acids lost H2O (18.01488) |
| M-H2O-OH | 138.9951 | 139.0036 | HO- is m/z=17.00694 |
| M-HO-HCO | 127.9924 | 128.0071 | HC=O is m/z=29.01764 |
| M-H2O-HOCO | 110.9855 | 111.0088 | HO-C=O is m/z=45.01664 |
Figure S4.10-1. (A) picoPPESI-MS/MS spectrum of putative Monodehydroascorbic acid peak (m/z 174.0170) obtained from the tissue extract (see Materials and Methods). Precursor ion m/z 174.0170; selector gate range m/z 173.8-174.2. Fragmentation was induced with a 10 % normalized collision energy. Because the MS/MS set range include several precursor ions, their fragment ions were assumed to be overlapped. The four signals with m/z shown in purple in A also match with the four fragments theoretically calculated from Monodehydroascorbic acid (B). Even the presence of other unknown precursor ions (Fig. S4.11-2), main fragments in the MS/MS spectrum can be assigned to the precursor ion [Monodehydroascorbic acid-H]-.

## Slide 13
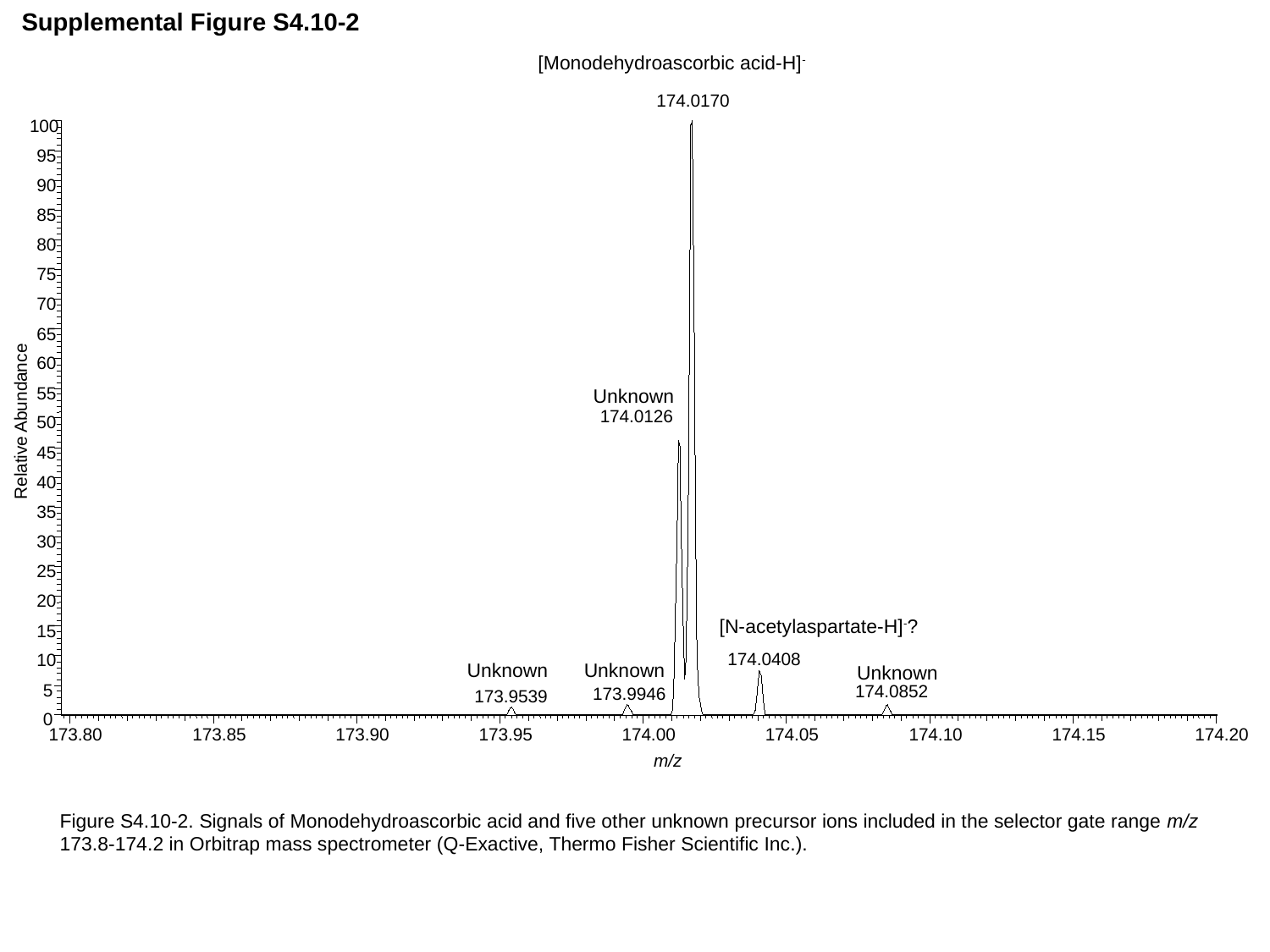

Supplemental Figure S4.10-2
[Monodehydroascorbic acid-H]-
174.0170
100
95
90
85
80
75
70
65
60
55
174.0126
Relative Abundance
50
45
40
35
30
25
20
15
174.0408
10
5
174.0852
173.9946
173.9539
0
173.80
173.85
173.90
173.95
174.00
174.05
174.10
174.15
174.20
m/z
Unknown
[N-acetylaspartate-H]-?
Unknown
Unknown
Unknown
Figure S4.10-2. Signals of Monodehydroascorbic acid and five other unknown precursor ions included in the selector gate range m/z 173.8-174.2 in Orbitrap mass spectrometer (Q-Exactive, Thermo Fisher Scientific Inc.).

## Slide 14
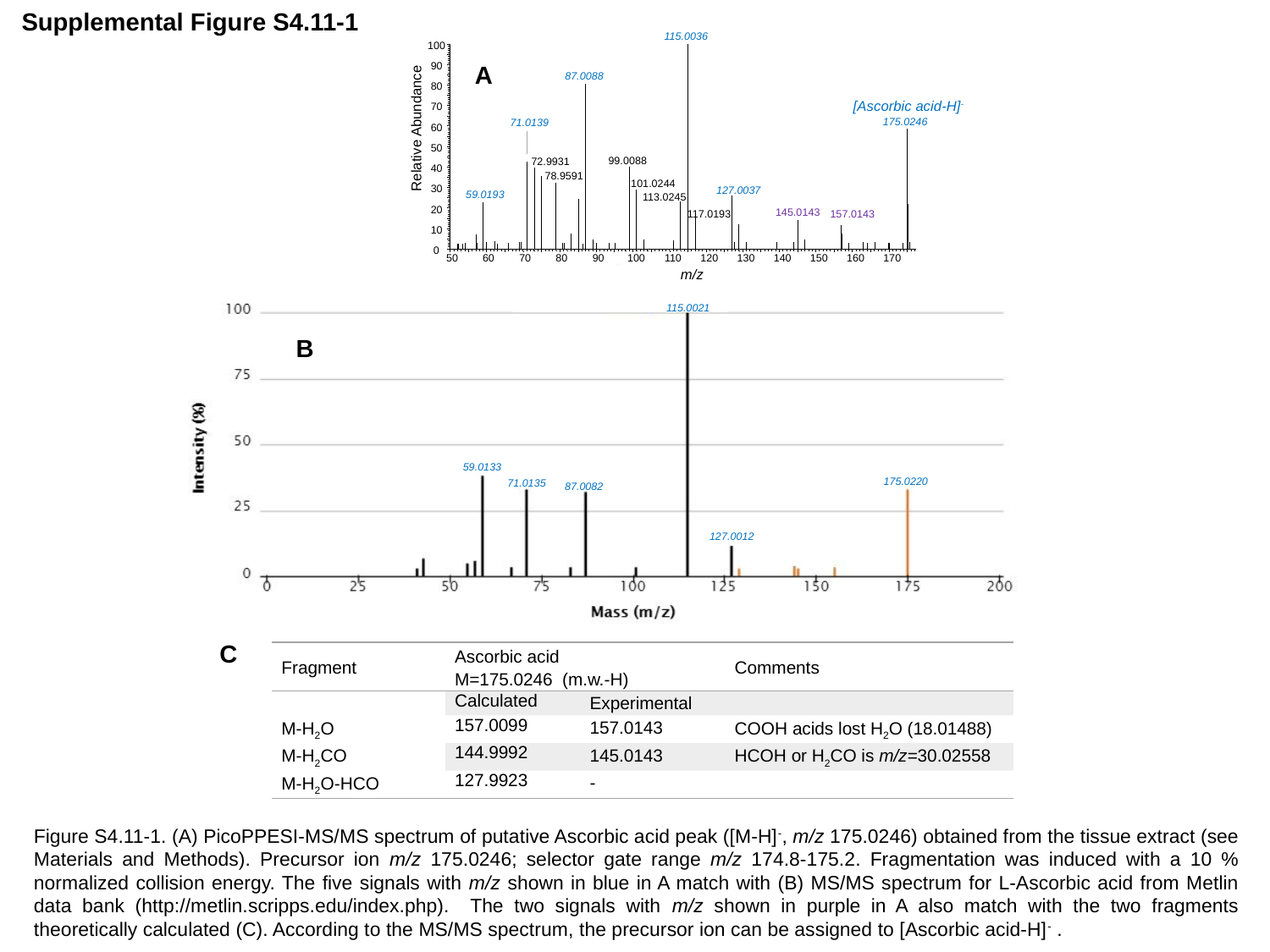

Supplemental Figure S4.11-1
115.0036
100
90
87.0088
80
[Ascorbic acid-H]-
70
Relative Abundance
175.0246
71.0139
60
50
99.0088
72.9931
40
78.9591
101.0244
30
127.0037
59.0193
113.0245
20
145.0143
157.0143
117.0193
10
0
50
60
70
80
90
100
110
120
130
140
150
160
170
m/z
A
115.0021
B
59.0133
175.0220
71.0135
87.0082
127.0012
C
| Fragment | Ascorbic acid M=175.0246 (m.w.-H) | | Comments |
| --- | --- | --- | --- |
| | Calculated | Experimental | |
| M-H2O | 157.0099 | 157.0143 | COOH acids lost H2O (18.01488) |
| M-H2CO | 144.9992 | 145.0143 | HCOH or H2CO is m/z=30.02558 |
| M-H2O-HCO | 127.9923 | - | |
Figure S4.11-1. (A) PicoPPESI-MS/MS spectrum of putative Ascorbic acid peak ([M-H]-, m/z 175.0246) obtained from the tissue extract (see Materials and Methods). Precursor ion m/z 175.0246; selector gate range m/z 174.8-175.2. Fragmentation was induced with a 10 % normalized collision energy. The five signals with m/z shown in blue in A match with (B) MS/MS spectrum for L-Ascorbic acid from Metlin data bank (http://metlin.scripps.edu/index.php). The two signals with m/z shown in purple in A also match with the two fragments theoretically calculated (C). According to the MS/MS spectrum, the precursor ion can be assigned to [Ascorbic acid-H]- .

## Slide 15
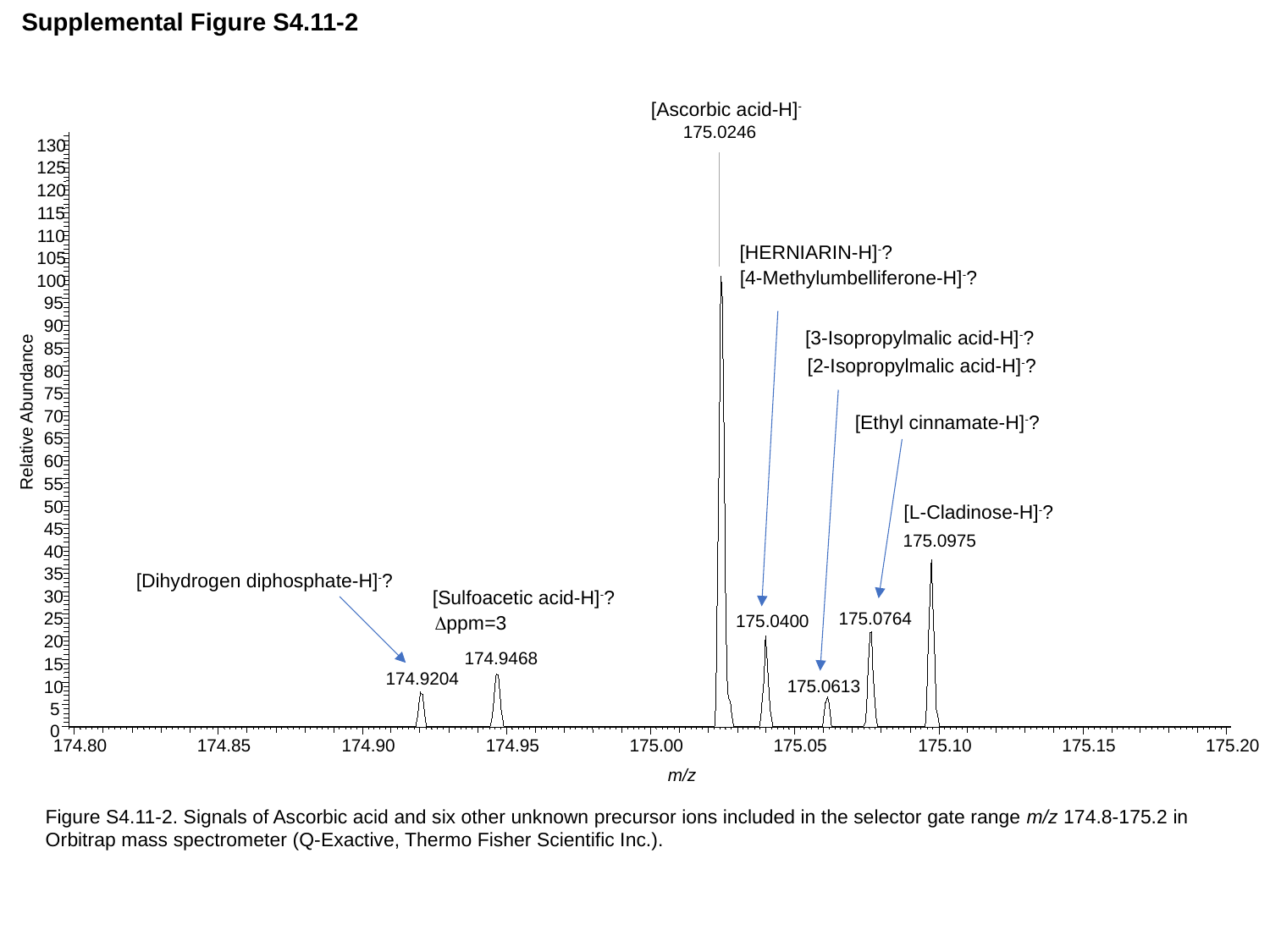

Supplemental Figure S4.11-2
[Ascorbic acid-H]-
175.0246
130
125
120
115
110
105
100
95
90
85
80
75
Relative Abundance
70
65
60
55
50
45
175.0975
40
35
30
Dppm=3
175.0764
25
175.0400
20
174.9468
15
174.9204
175.0613
10
5
0
174.80
174.85
174.90
174.95
175.00
175.05
175.10
175.15
175.20
m/z
[HERNIARIN-H]-?
[4-Methylumbelliferone-H]-?
[3-Isopropylmalic acid-H]-?
[2-Isopropylmalic acid-H]-?
[Ethyl cinnamate-H]-?
[L-Cladinose-H]-?
[Dihydrogen diphosphate-H]-?
[Sulfoacetic acid-H]-?
Figure S4.11-2. Signals of Ascorbic acid and six other unknown precursor ions included in the selector gate range m/z 174.8-175.2 in Orbitrap mass spectrometer (Q-Exactive, Thermo Fisher Scientific Inc.).

## Slide 16
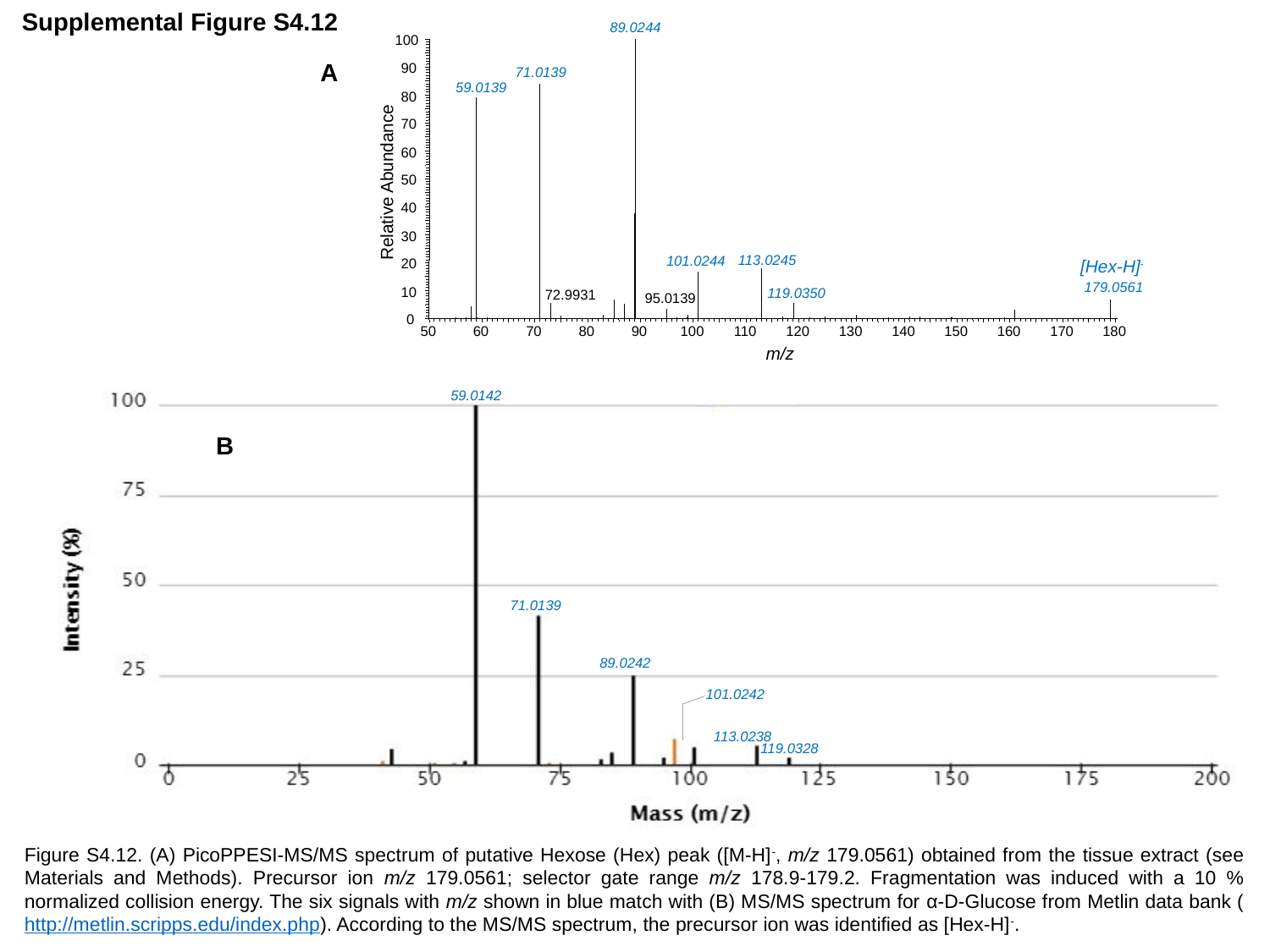

Supplemental Figure S4.12
89.0244
100
90
71.0139
59.0139
80
70
Relative Abundance
60
50
40
30
113.0245
101.0244
20
179.0561
10
119.0350
72.9931
95.0139
0
50
60
70
80
90
100
110
120
130
140
150
160
170
180
m/z
A
[Hex-H]-
59.0142
B
71.0139
89.0242
101.0242
113.0238
119.0328
Figure S4.12. (A) PicoPPESI-MS/MS spectrum of putative Hexose (Hex) peak ([M-H]-, m/z 179.0561) obtained from the tissue extract (see Materials and Methods). Precursor ion m/z 179.0561; selector gate range m/z 178.9-179.2. Fragmentation was induced with a 10 % normalized collision energy. The six signals with m/z shown in blue match with (B) MS/MS spectrum for α-D-Glucose from Metlin data bank (http://metlin.scripps.edu/index.php). According to the MS/MS spectrum, the precursor ion was identified as [Hex-H]-.

## Slide 17
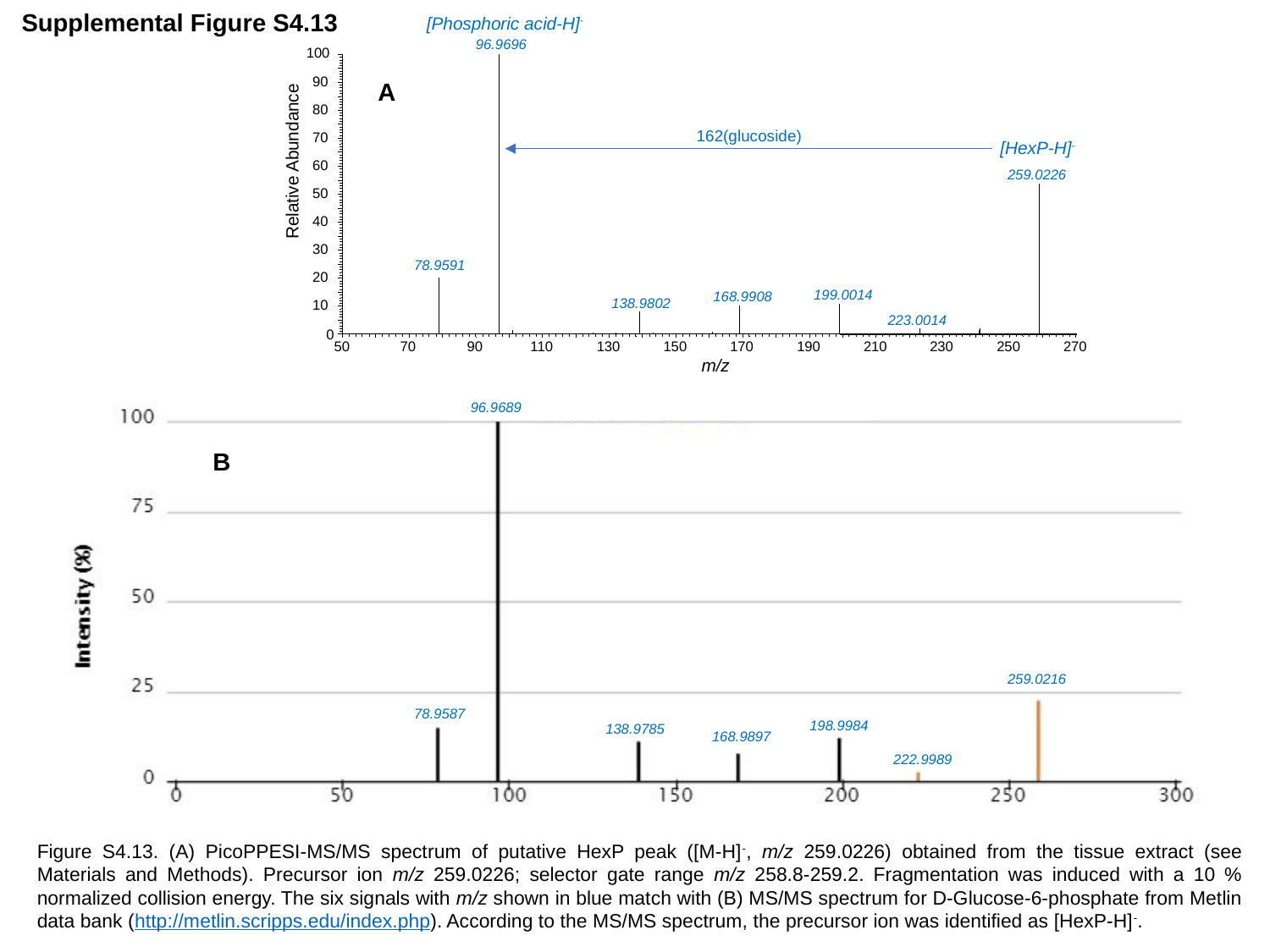

Supplemental Figure S4.13
[Phosphoric acid-H]-
96.9696
100
90
80
70
[HexP-H]-
Relative Abundance
60
259.0226
50
40
30
78.9591
20
199.0014
168.9908
138.9802
10
223.0014
0
50
70
90
110
130
150
170
190
210
230
250
270
m/z
A
96.9689
B
259.0216
78.9587
198.9984
138.9785
168.9897
222.9989
Figure S4.13. (A) PicoPPESI-MS/MS spectrum of putative HexP peak ([M-H]-, m/z 259.0226) obtained from the tissue extract (see Materials and Methods). Precursor ion m/z 259.0226; selector gate range m/z 258.8-259.2. Fragmentation was induced with a 10 % normalized collision energy. The six signals with m/z shown in blue match with (B) MS/MS spectrum for D-Glucose-6-phosphate from Metlin data bank (http://metlin.scripps.edu/index.php). According to the MS/MS spectrum, the precursor ion was identified as [HexP-H]-.

## Slide 18
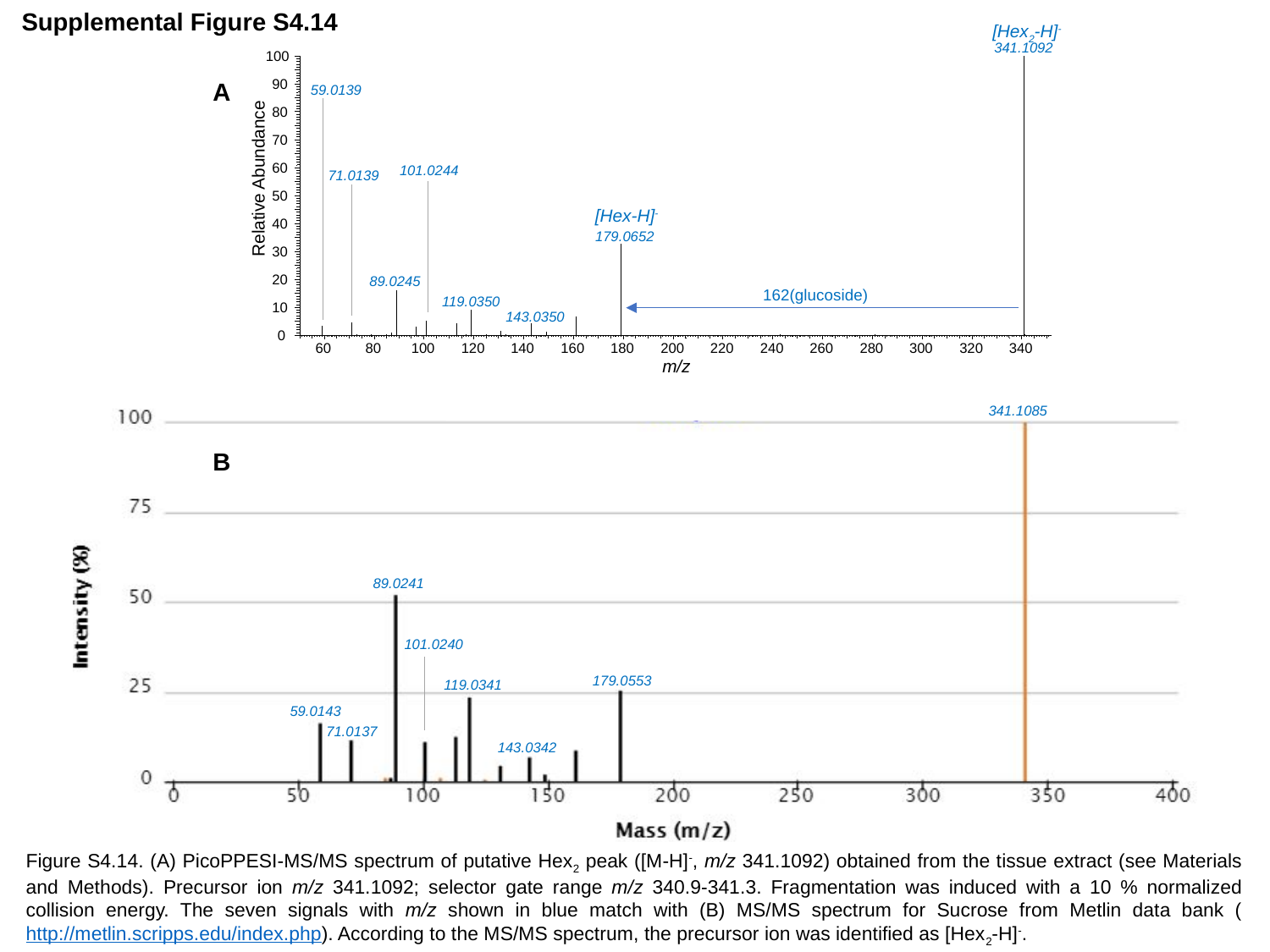

Supplemental Figure S4.14
[Hex2-H]-
341.1092
100
60
80
100
120
140
160
180
200
220
240
260
280
300
320
340
m/z
90
59.0139
80
70
Relative Abundance
60
101.0244
71.0139
50
[Hex-H]-
40
179.0652
30
20
89.0245
119.0350
10
143.0350
0
A
341.1085
B
89.0241
101.0240
179.0553
119.0341
59.0143
71.0137
143.0342
Figure S4.14. (A) PicoPPESI-MS/MS spectrum of putative Hex2 peak ([M-H]-, m/z 341.1092) obtained from the tissue extract (see Materials and Methods). Precursor ion m/z 341.1092; selector gate range m/z 340.9-341.3. Fragmentation was induced with a 10 % normalized collision energy. The seven signals with m/z shown in blue match with (B) MS/MS spectrum for Sucrose from Metlin data bank (http://metlin.scripps.edu/index.php). According to the MS/MS spectrum, the precursor ion was identified as [Hex2-H]-.

## Slide 19
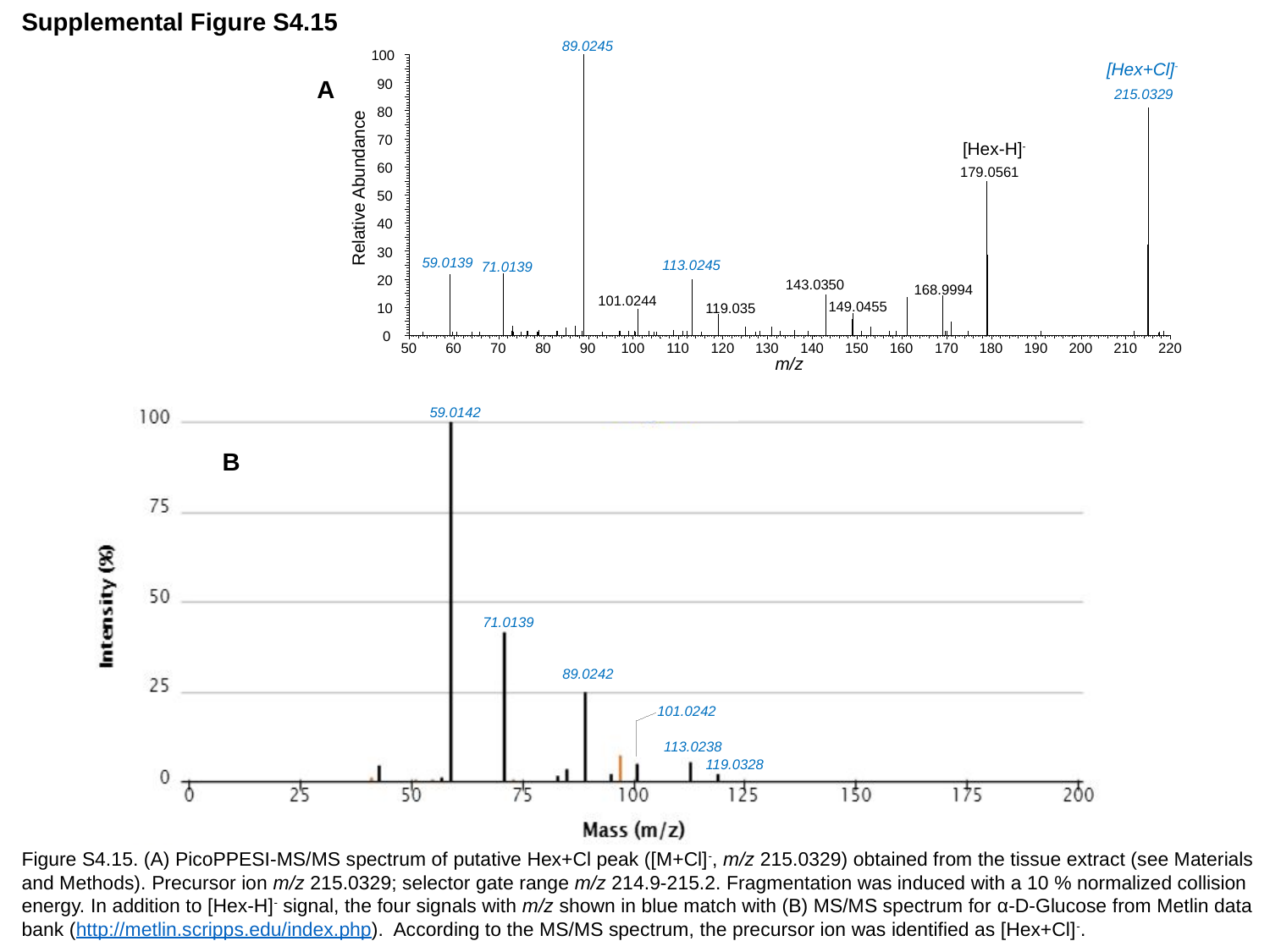

Supplemental Figure S4.15
89.0245
100
50
60
70
80
90
100
110
120
130
140
150
160
170
180
190
200
210
220
m/z
90
215.0329
80
70
Relative Abundance
60
179.0561
50
40
30
59.0139
113.0245
71.0139
20
143.0350
168.9994
101.0244
149.0455
10
119.035
0
[Hex+Cl]-
[Hex-H]-
A
59.0142
B
71.0139
89.0242
101.0242
113.0238
119.0328
Figure S4.15. (A) PicoPPESI-MS/MS spectrum of putative Hex+Cl peak ([M+Cl]-, m/z 215.0329) obtained from the tissue extract (see Materials and Methods). Precursor ion m/z 215.0329; selector gate range m/z 214.9-215.2. Fragmentation was induced with a 10 % normalized collision energy. In addition to [Hex-H]- signal, the four signals with m/z shown in blue match with (B) MS/MS spectrum for α-D-Glucose from Metlin data bank (http://metlin.scripps.edu/index.php). According to the MS/MS spectrum, the precursor ion was identified as [Hex+Cl]-.

## Slide 20
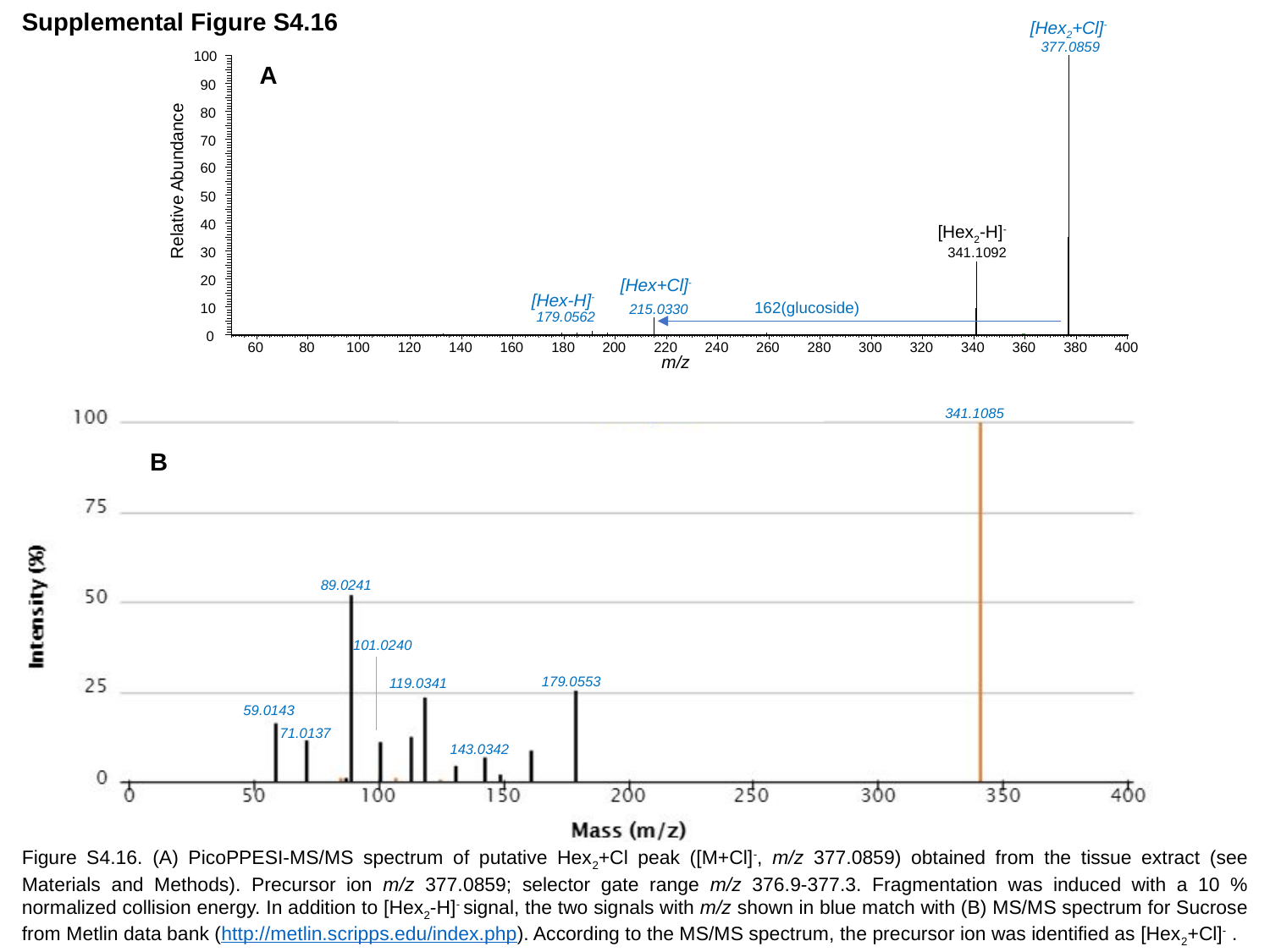

Supplemental Figure S4.16
[Hex2+Cl]-
377.0859
100
60
80
100
120
140
160
180
200
220
240
260
280
300
320
340
360
380
400
m/z
90
80
70
Relative Abundance
60
50
[Hex2-H]-
40
30
341.1092
[Hex+Cl]-
20
[Hex-H]-
10
215.0330
179.0562
0
A
341.1085
B
89.0241
101.0240
179.0553
119.0341
59.0143
71.0137
143.0342
Figure S4.16. (A) PicoPPESI-MS/MS spectrum of putative Hex2+Cl peak ([M+Cl]-, m/z 377.0859) obtained from the tissue extract (see Materials and Methods). Precursor ion m/z 377.0859; selector gate range m/z 376.9-377.3. Fragmentation was induced with a 10 % normalized collision energy. In addition to [Hex2-H]- signal, the two signals with m/z shown in blue match with (B) MS/MS spectrum for Sucrose from Metlin data bank (http://metlin.scripps.edu/index.php). According to the MS/MS spectrum, the precursor ion was identified as [Hex2+Cl]- .

## Slide 21
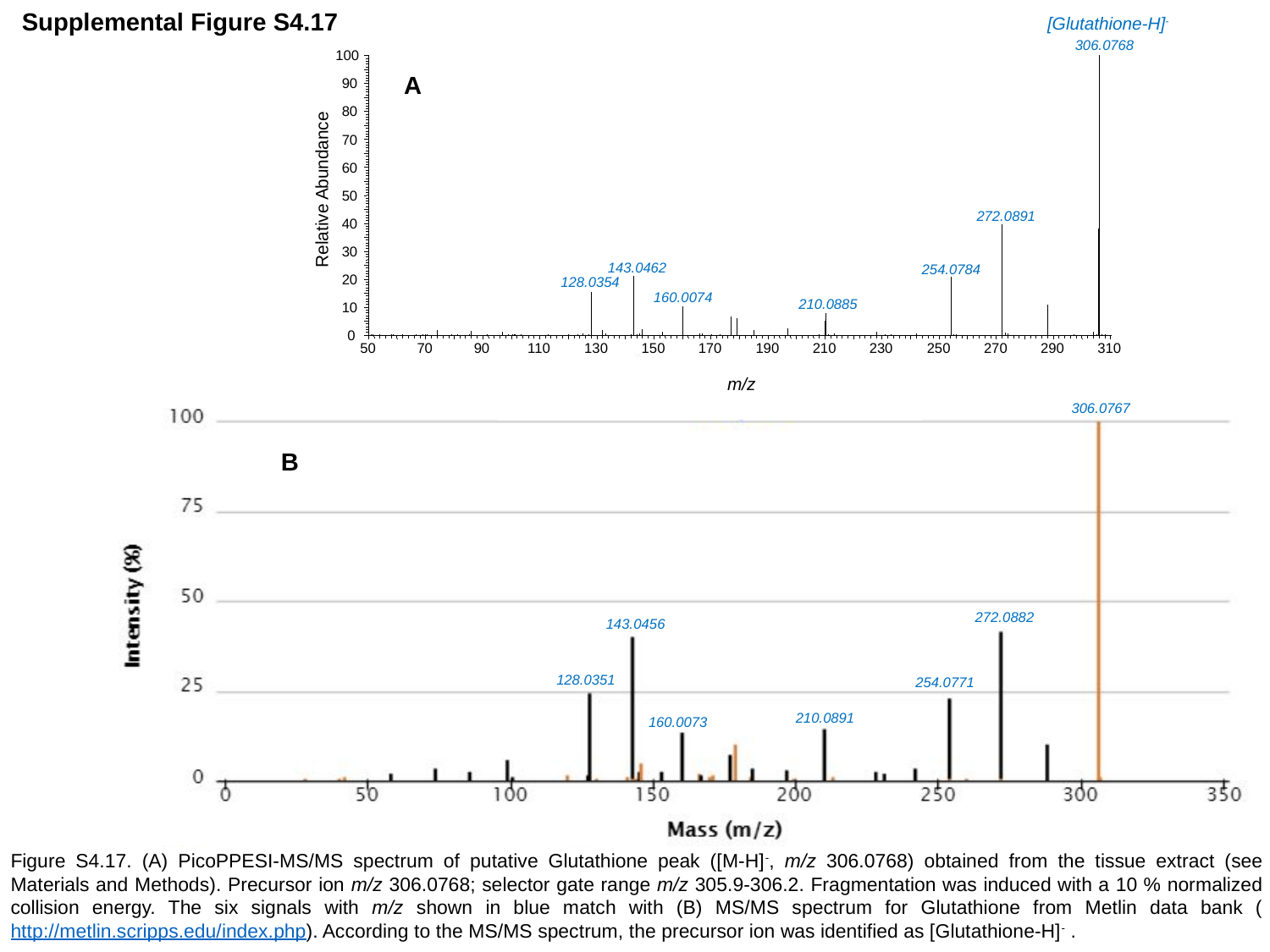

Supplemental Figure S4.17
[Glutathione-H]-
306.0768
100
90
80
70
Relative Abundance
60
50
272.0891
40
30
143.0462
254.0784
20
128.0354
160.0074
210.0885
10
0
50
70
90
110
130
150
170
190
210
230
250
270
290
310
A
m/z
306.0767
B
272.0882
143.0456
128.0351
254.0771
210.0891
160.0073
Figure S4.17. (A) PicoPPESI-MS/MS spectrum of putative Glutathione peak ([M-H]-, m/z 306.0768) obtained from the tissue extract (see Materials and Methods). Precursor ion m/z 306.0768; selector gate range m/z 305.9-306.2. Fragmentation was induced with a 10 % normalized collision energy. The six signals with m/z shown in blue match with (B) MS/MS spectrum for Glutathione from Metlin data bank (http://metlin.scripps.edu/index.php). According to the MS/MS spectrum, the precursor ion was identified as [Glutathione-H]- .

## Slide 22
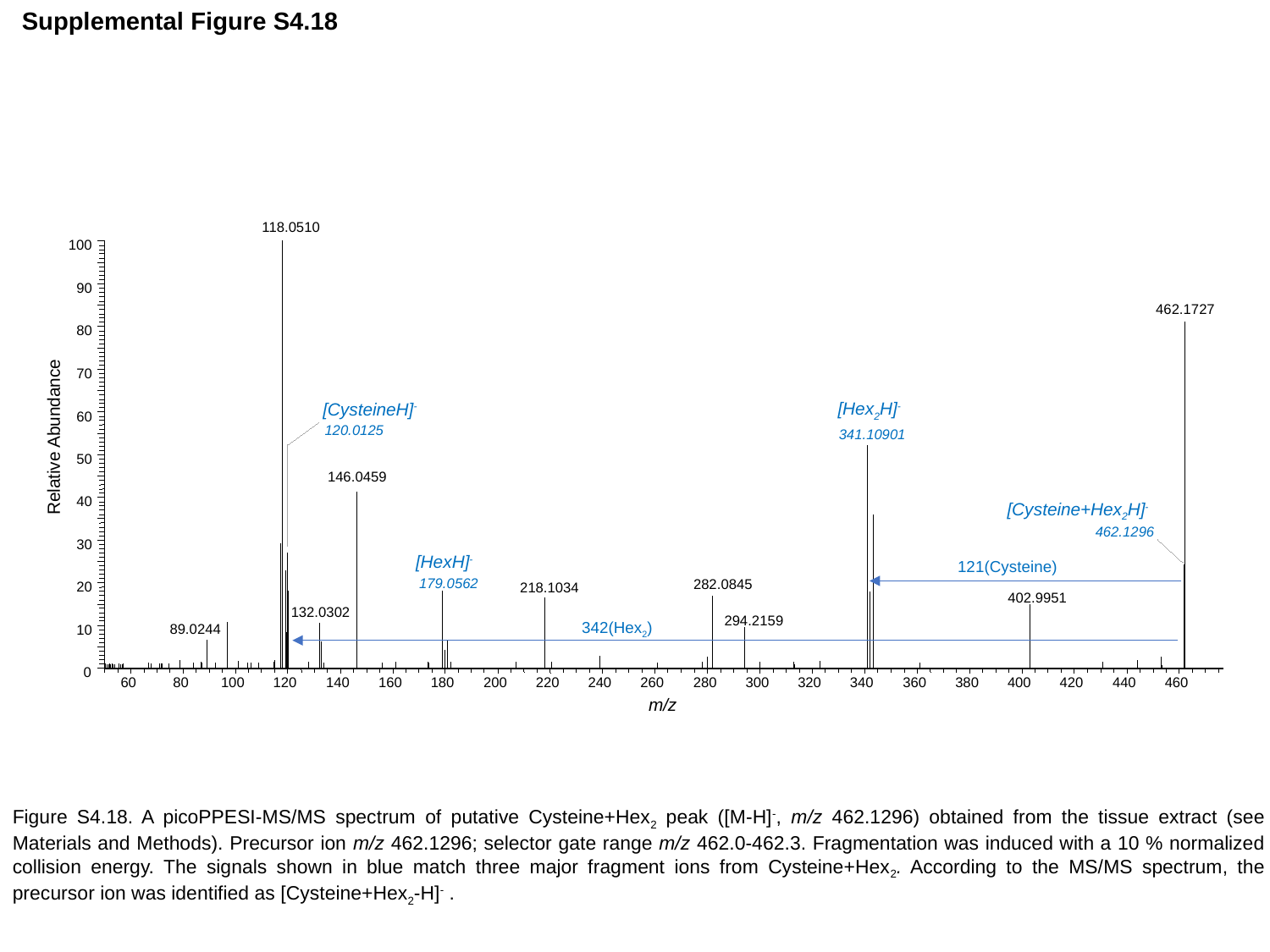

Supplemental Figure S4.18
118.0510
100
90
462.1727
80
70
60
120.0125
Relative Abundance
341.10901
50
146.0459
40
462.1296
30
179.0562
282.0845
20
218.1034
402.9951
132.0302
294.2159
89.0244
10
0
60
80
100
120
140
160
180
200
220
240
260
280
300
320
340
360
380
400
420
440
460
m/z
Figure S4.18. A picoPPESI-MS/MS spectrum of putative Cysteine+Hex2 peak ([M-H]-, m/z 462.1296) obtained from the tissue extract (see Materials and Methods). Precursor ion m/z 462.1296; selector gate range m/z 462.0-462.3. Fragmentation was induced with a 10 % normalized collision energy. The signals shown in blue match three major fragment ions from Cysteine+Hex2. According to the MS/MS spectrum, the precursor ion was identified as [Cysteine+Hex2-H]- .

## Slide 23
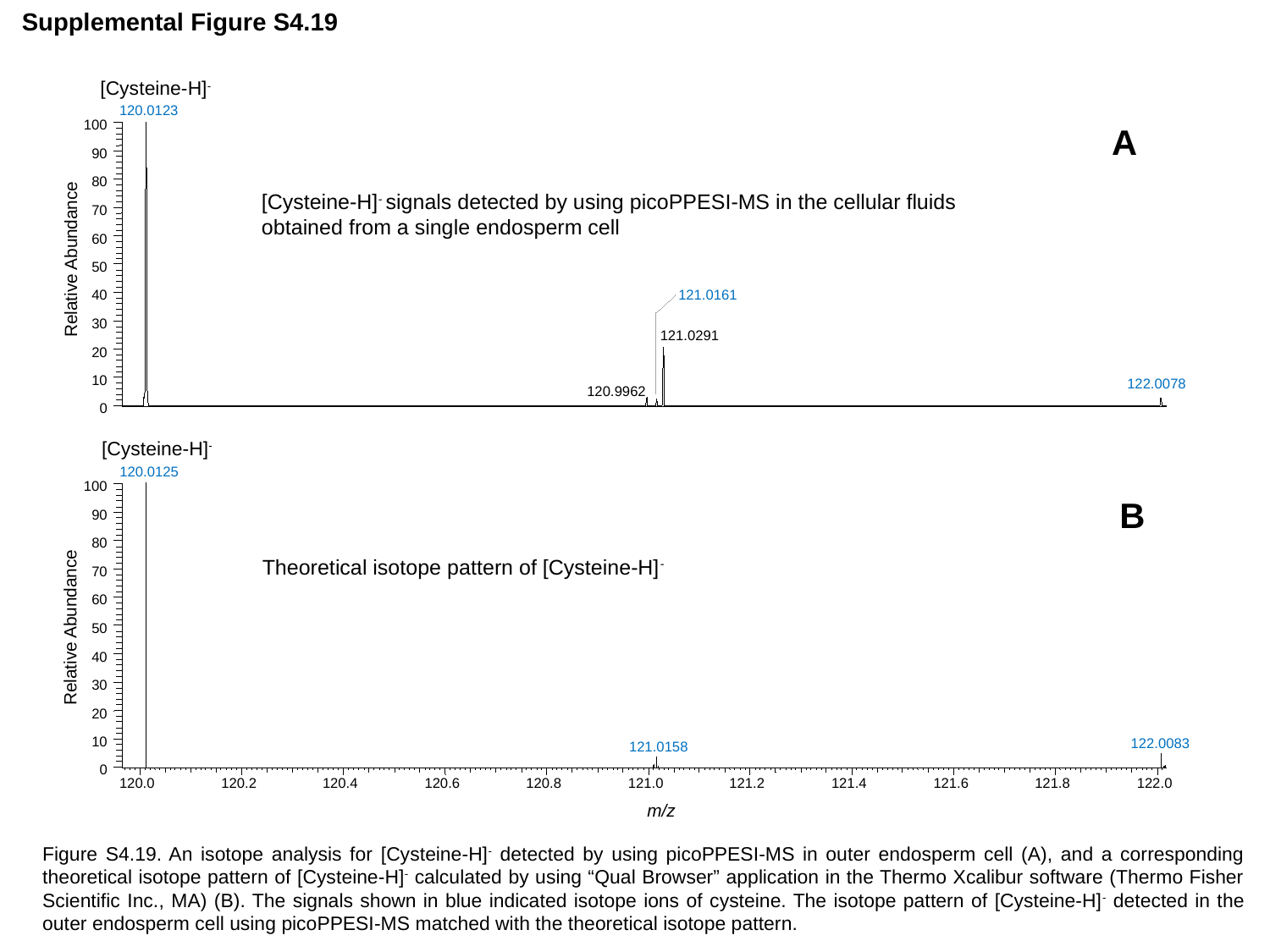

Supplemental Figure S4.19
[Cysteine-H]-
120.0123
100
90
80
70
60
Relative Abundance
50
121.0161
40
30
121.0291
20
10
122.0078
120.9962
0
A
[Cysteine-H]- signals detected by using picoPPESI-MS in the cellular fluids obtained from a single endosperm cell
[Cysteine-H]-
120.0125
100
90
80
70
60
50
40
30
20
10
122.0083
121.0158
0
120.0
120.2
120.4
120.6
120.8
121.0
121.2
121.4
121.6
121.8
122.0
m/z
B
Theoretical isotope pattern of [Cysteine-H]-
Relative Abundance
Figure S4.19. An isotope analysis for [Cysteine-H]- detected by using picoPPESI-MS in outer endosperm cell (A), and a corresponding theoretical isotope pattern of [Cysteine-H]- calculated by using “Qual Browser” application in the Thermo Xcalibur software (Thermo Fisher Scientific Inc., MA) (B). The signals shown in blue indicated isotope ions of cysteine. The isotope pattern of [Cysteine-H]- detected in the outer endosperm cell using picoPPESI-MS matched with the theoretical isotope pattern.
